# Hybridization Approach Applied to Umbelliferon and Vanilloids toward New Inhibitors of Carbonic Anhydrases IX and XII with In Vitro Antiproliferative and Anti-inflammatory Activities

**DOI:** 10.1021/acs.jmedchem.5c02930

**Published:** 2026-01-08

**Authors:** Francesco Melfi, Noemi Mencarelli, Simone Carradori, Marialucia Gallorini, Andrea Angeli, Giulio Poli, Amelia Cataldi, Ilaria D’Agostino, Andrea Di Credico, Angela Di Baldassarre, Tiziano Tuccinardi, Claudiu T. Supuran

**Affiliations:** † Department of Pharmacy, “G. d’Annunzio” University of Chieti-Pescara, via dei Vestini 31, 66100 Chieti, Italy; ‡ NEUROFARBA Department, Pharmaceutical and Nutraceutical Section, 9300 University of Firenze, via Ugo Schiff 6, 50019 Sesto Fiorentino, Firenze, Italy; § Department of Pharmacy, 9310 University of Pisa, Via Bonanno 6, 56126 Pisa, Italy; ∥ Department of Medicine and Aging Sciences, ″G. d’Annunzio″, University of Chieti-Pescara, 66100 Chieti, Italy; ⊥ UdA-TechLab, ″G. d’Annunzio″, University of Chieti-Pescara, 66100 Chieti, Italy

## Abstract

Human carbonic anhydrases (hCAs) IX and XII have emerged as promising therapeutic targets and are overexpressed in hypoxic tumors. Leveraging the chemotype of umbelliferon (**UMB**), as a selective hCAs IX and XII inhibitor, we designed and synthesized several hybrids (**7**–**33**) connecting **UMB** natural scaffold with vanilloids by using methylene spacers or triazole linkers. These hybrids demonstrated nanomolar inhibitory activity against the tumor-associated hCAs IX and XII. Molecular modeling and dynamics simulations revealed stable hydrogen bonding and hydrophobic interactions. In vitro evaluation of human bronchial epithelial (BEAS-2B) and lung adenocarcinoma (A549) cell lines showed selective cytotoxicity against cancer cells. Selected compounds induced G1 cell cycle arrest, reduced expression of the metastasis-associated markers CD9 and epithelial cell adhesion molecule, and exhibited cytoprotective and anti-inflammatory effects in BEAS-2B cells. Collectively, these findings identify **UMB**-vanilloid hybrids as promising candidates for the development of novel therapeutics for nonsmall cell lung cancer.

## Graphical abstract

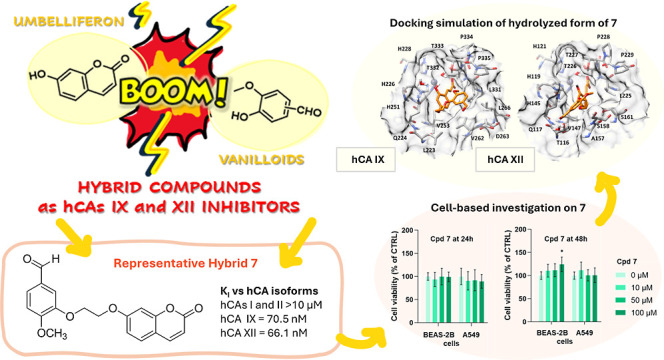

## 1 Introduction

Lung cancer is the most commonly diagnosed cancer and the leading cause of cancer-related death worldwide, accounting for approximately 12.4% of all new tumor cases.^1^ Nonsmall cell lung cancer (NSCLC) represents about 85% of all lung cancer cases,^2^ with lung adenocarcinoma being the most prevalent and lethal subtype, due to its molecular heterogeneity and typically late-stage diagnosis.^3^ Standard treatments, often including pemetrexed alone or in combination with chemotherapy or radiotherapy, are often limited by drug resistance and side effects. Although recent precision medicine introduced targeted treatments for specific genetic mutations such as EGFR, ALK, and ROS1, significantly improved outcomes for certain genetic subsets, a large proportion of patients still lack effective therapeutic options.^4^

In this scenario, a deeper understanding of the molecular mechanisms behind NSCLC development and progression and the identification of new therapeutic targets remain crucial and challenging. Among these, human (h) carbonic anhydrases (CAs, EC: 4.2.1.1) IX and XII have emerged. CAs are a superfamily of metalloenzymes, responsible for the reversible hydration of carbon dioxide (CO_2_) to bicarbonate (HCO_3_^–^) and protons (H^+^) and involved in many physiopathological pathways, including homeostasis, pH balance, and CO_2_ fixation.^5,6^ Isoforms IX and XII are transmembrane isoenzymes, overexpressed in various solid tumors under hypoxic conditions, including lung cancer,^7–9^ that are involved in regulating pH and sustaining the acidic tumor microenvironment, thereby promoting lung cancer progression, metastasis, and therapy resistance.^10–13^ High expression of hCAsIX and XII has been linked to worse survival in lung cancer patients, with the isoform IX being associated with poor prognosis and aggressive NSCLC phenotypes.^14^ Additionally, hCA XII supports the function of P-glycoprotein (Pgp), which is a key efflux pump responsible for drug resistance. By maintaining Pgp activity, hCA XII enhances the clearance of chemotherapeutic agents, promoting cancer cell survival and resistance to treatment.^15^

Besides CAs, specific surface proteins were found to contribute to NSCLC progression. In particular, the epithelial cell adhesion molecule (EpCAM) is frequently overexpressed in epithelial tumors and promotes proliferation, migration, and the epithelial-to-mesenchymal transition (EMT). CD9, a member of the tetraspanin family, is involved in cell adhesion, signaling, and the regulation of exosome release, and it shows context-dependent roles in cancer, with its downregulation being associated with increased cell motility, high aggressivity, and poor prognosis in NSCLC patients.^16,17^

The molecular alterations are sustained by a pro-inflammatory tumor microenvironment, aligned with the complex transition from inflammation to tumorigenesis. While it is well established that environmental stimuli can trigger inflammation, the oncogenic changes driven by chronic inflammation within the tissue microenvironment are less well understood. In particular, the mechanisms by which these changes initiate and promote pro-tumorigenic processes remain to be fully elucidated.^18^ Chronic inflammation of the bronchial epithelial cells, which represents the first line of defense in the respiratory tract, can initiate and sustain inflammatory responses upon exposure to environmental insults such as pathogens, pollutants, or cigarette smoke, and fostered by the transcription factor NF-kB signaling, lead to the release of pro-inflammatory cytokines and chemokines, including interleukins IL-6, IL-8, and Tumor-Necrosis Factor-α (TNF-α), which recruit and activate immune cells, further amplifying the inflammatory milieu.^19^

In this complex landscape, Nature has long served as a rich source of pharmacologically active compounds, with several plant-derived metabolites (e.g., coumarins, phenolic compounds) showing relevant anti-inflammatory and anticancer properties, revealing a high potential for application in the treatment of human diseases, as also largely exploited by traditional herbal medicine.^20–22^ Also, several natural compounds have gained attention as multitatget anticancer agents due to their structural diversity and ability to modulate key tumor-related patways, Among these, 7-hydroxycoumarin, commonly known as umbelliferon (**UMB**, **1**, Figure 1), is a coumarin derivative distributed in numerous plant families such as Umbelliferae, Fabaceae, and Rutaceae.^23,24^

**Figure 1:**
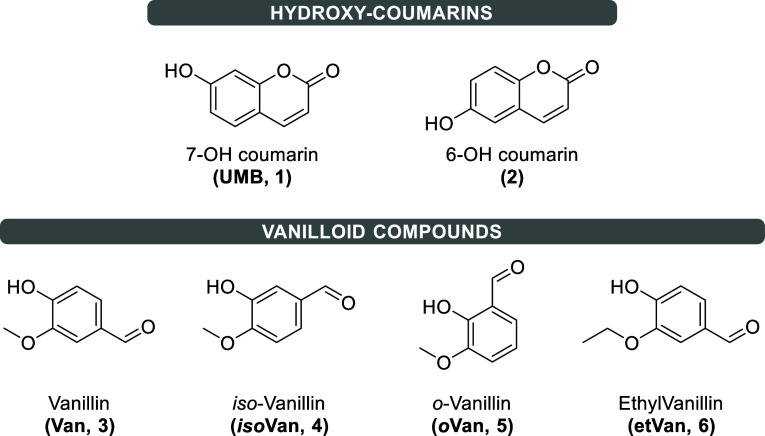
Structures of coumarins (7- and 6-hydroxy derivatives, **UMB** **1**, and **2**) along with vanilloids (**3–6**).

**UMB** and its isomer 6-hydroxycoumarin (**2**, Figure 1) have a multifaceted biological profile, including antioxidant, anti-inflammatory, and anticancer properties, which are exerted through multiple mechanisms, ranging from oxidative stress modulation to apoptosis regulation.^23,25,26^ Of particular interest, certain coumarins like **1** and **2** were found to act as suicide inhibitors of CAs due to their esterase activity (as reported for most representative α-, η-, and ι-CA classes to date).^27–29^ Specifically, the compact nucleus in 2*H*-chromene-2-one binds near the entrance of the CA catalytic site and undergoes lactone hydrolysis, which frees the hydroxycinnamic acid product, the “opened” form, that remains bound to the enzyme zinc-bound water molecule, leading to the catalytic activity inhibition due to steric hindrance.^30–32^

Building on this mechanism, several **UMB** derivatives have been synthesized with the aim of improving CA inhibition and anticancer activity.^33–37^ For instance, monoterpene–coumarin hybrids designed using the tail approach demonstrated selective inhibition of tumor-associated CA isoforms and cytotoxicity against cancer cells.^38^ This strategy involves decorating the coumarin scaffold with functional tails to enhance the target engagement and bioactivity. Inspired by these findings, we propose to investigate the interconnection between chronic inflammation and tumorigenesis, especially in lung cancer, by designing and studying the effect of a series of hybrid derivatives of **UMB**, selected as hCAs IX and XII-inhibiting pharmacophore, and another natural compounds scaffold, that of vanilloids (**3–6**, Figure 1).

Like other natural phenolic compounds,^33,39,40^ vanilloids are also endowed with antioxidant and anti-inflammatory activity. The parent natural compound, Vanillin (**Van**, Figure 1), is reported to possess strong antitumor properties, likely due to its activity on membrane-bound receptors such as the transient receptor potential vanilloid type 1 (TRPV1) receptor as well as intracellular targets including MARK4, CAMK4, and CK2, which are implicated in cancer cell proliferation, apoptosis, and migration. Also, it was shown to modulate cellular redox balance and DNA repair pathways.^41^ In this context, we designed and synthesized a series of **UMB**–vanilloid conjugates through linear aliphatic spacers or copper-catalyzed azide–alkyne cycloaddition (CuAAC)-derived triazoles, aiming to combine the CA inhibitory properties of the coumarin core with the bioactivity of vanilloids. Our strategy was expected to enhance the pharmacological profile of the parent natural compounds and allow for the modular introduction of structural diversity to optimize target engagement in NSCLC. Aliphatic linkers were selected to explore conformational space and modulate the length between the two main scaffolds, whereas triazoles can be useful to improve aqueous solubility and metabolic stability.

## 2 Results and Discussion

### 2.1 Design and Synthesis of the Derivatives Library

A wide library of hybrids was designed as hCAs IX and XII inhibitors and synthesized (Figure 2). The derivatives are composed of the **UMB** scaffold and vanilloid moiety, linked together through methylene spacers, in series *A* (**7–24**), or click chemistry-generated 1,2,3-triazoles, in series *B* (**25**–**33**).

**Figure 2:**
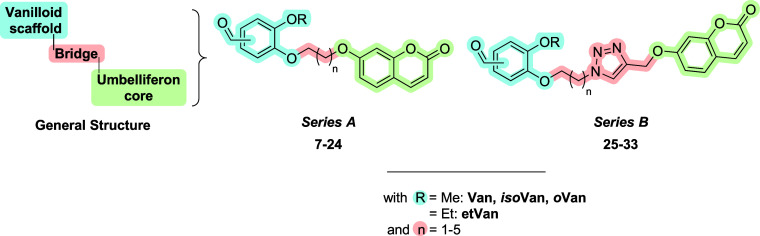
General structures of the derivatives library, series *A* (**7–24**) and *B* (**25–33**), reported in this work.

In detail, series *A* derivatives (**7**–**24**) were obtained by performing two subsequent microwave-assisted nucleophilic substitutions in the presence of potassium carbonate in acetonitrile, as illustrated in Scheme 1. Moreover, the proper vanilloid (**3–6**) was first reacted with the suitable alkyl dihalide **a–e** to afford intermediates **34–37**, which undergo reaction with **UMB** (**1**). As for series *B* derivatives, intermediates **34–37** were converted into the corresponding azide (**38–41**) and were reacted with propargyl umbelliferon (**42**), obtained by reacting **UMB** (**1**) with propargyl bromide (Scheme 1). The latter reacted with the proper azide (**38–41**) via Huisgen CuAAC reaction, affording the 1,4-disubstituted triazole derivatives (**25**–**33**).

**Scheme 1:**
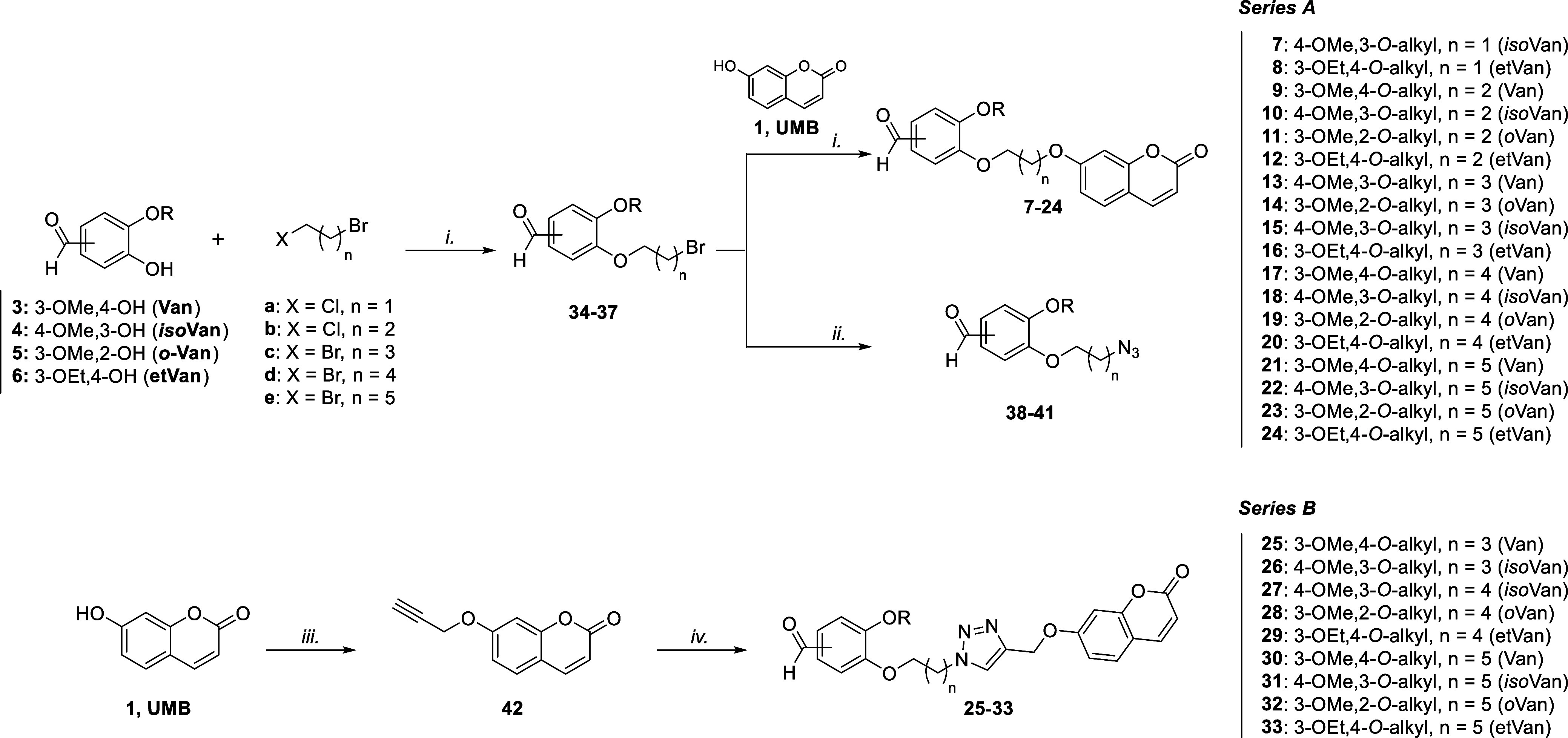
Synthesis of Compounds **7–33** ^a^

a Reagents and conditions: (i) K_2_CO_3_, ACN, microwave, 100–120 °C, 2–3 h; (ii) NaN_3_, DMF, r.t., 2 h; (iii) propargyl bromide, K_2_CO_3_, ACN, reflux, 12 h; (iv) suitable **38-41**, CuSO_4_·5H_2_O, sodium ascorbate, H_2_O/*t*BuOH 1:1, r.t., overnight.

### 2.2 Carbonic Anhydrase Inhibition Assay

Hybrid compounds **7–33** were profiled in vitro through the stopped-flow CO_2_ hydration assay^42^ for their ability to modulate the hypoxic-tumor-related hCAs IX and XII, along with the abundantly expressed isoforms I and II. The obtained inhibition constant (*K*_I_) values are reported in Table 1 and compared to the reference compounds **UMB** (**1**), vanilloids (**3–6**), the **UMB** intermediate **42**, and the pan-CA inhibitor acetazolamide (**AAZ**).

**Table 1:**
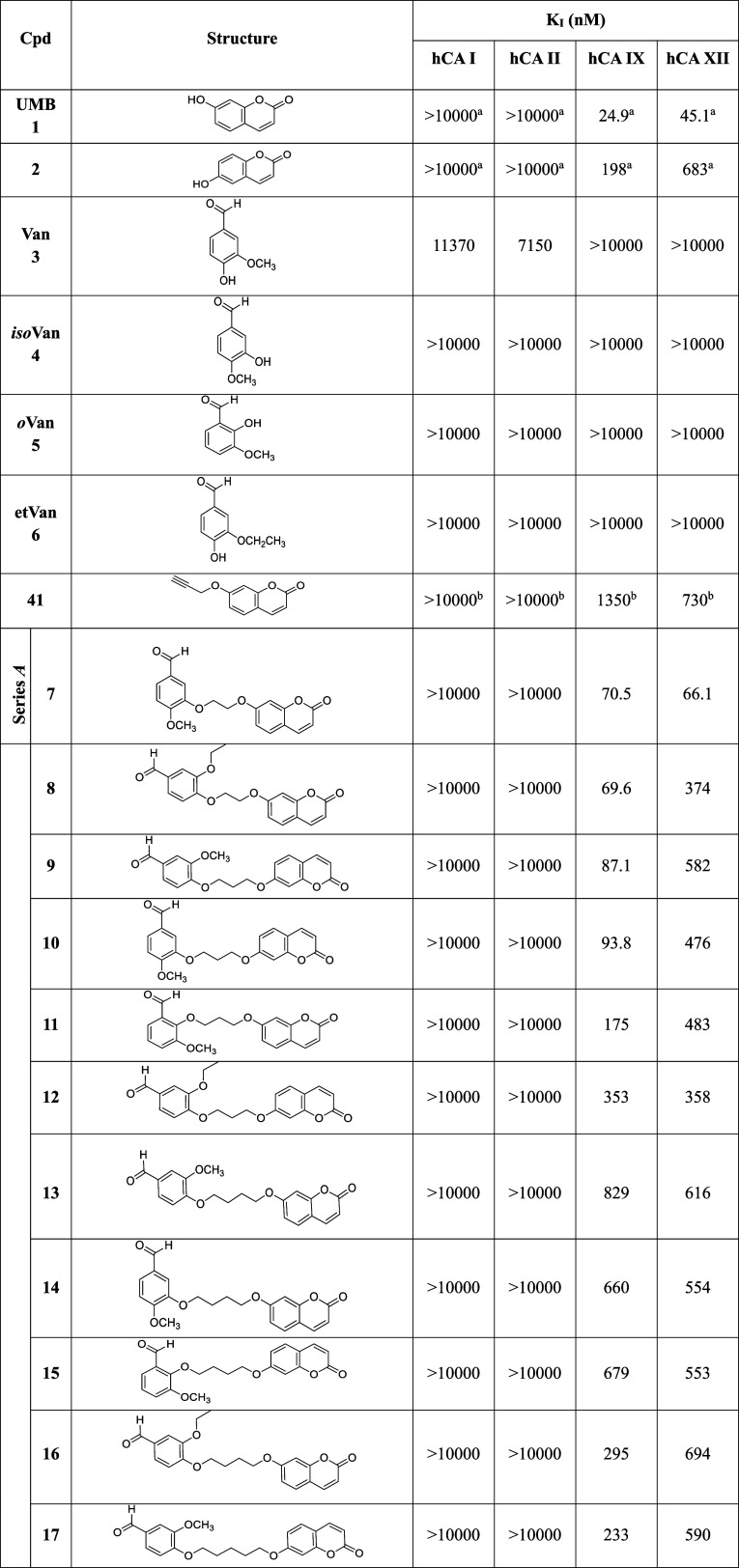

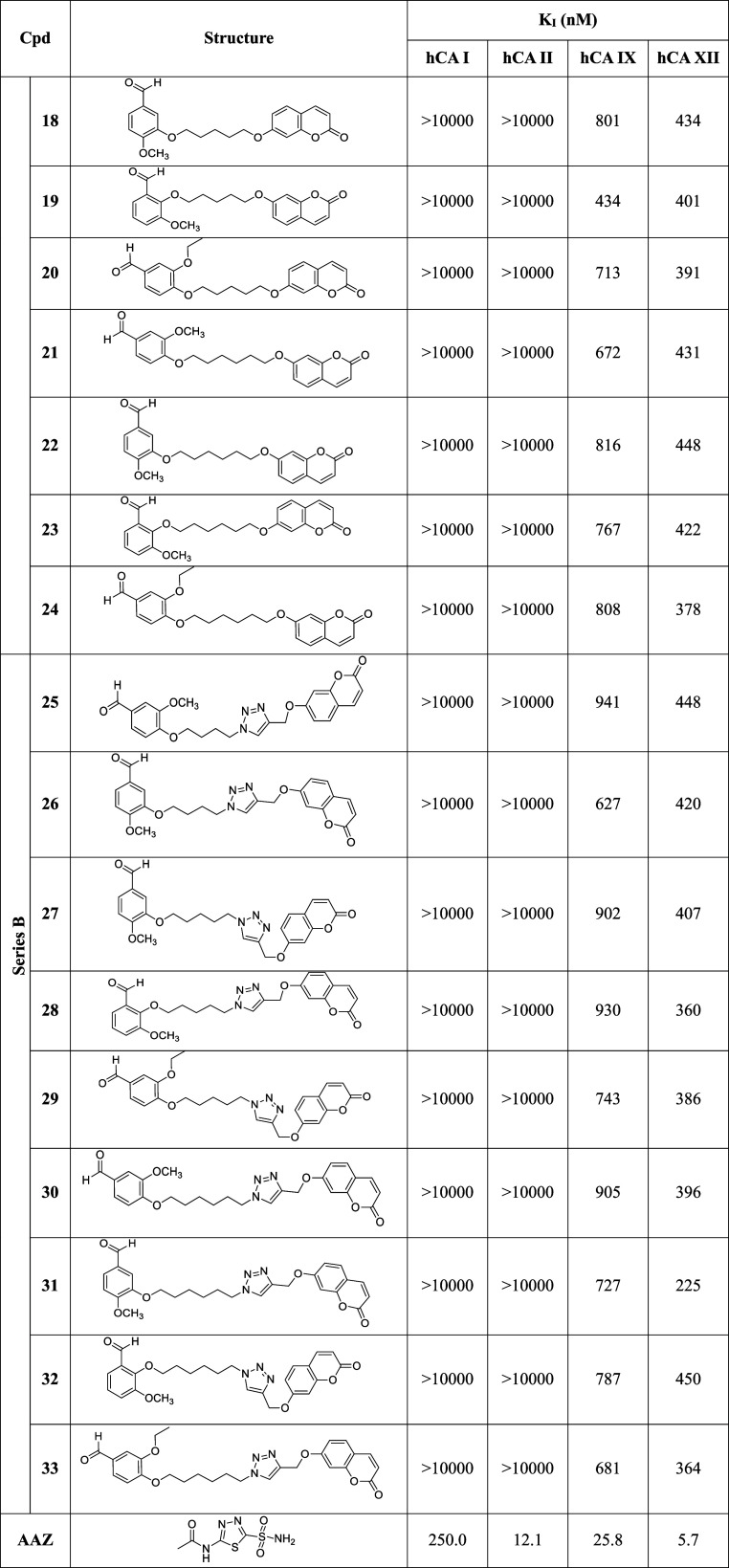
Inhibitory Data for Parent Phenolic Compounds (**1**, **2**, and **3**–**6**), Intermediate **42**, and Final Compounds **7**–**33** on a Panel of hCA Isoforms

a *K*_I_ values are reported as the mean from three different assays. Errors were in the range of ±5–10% of the reported values (data not shown).^a–b^ Data already reported by (a) Maresca and Supuran, 2010,^43^ and (b) Berrino et al., 2023.^44^

As can be observed in Table 1, all the hybrids (**7**–**33**) showed promising *K*_I_ values against the two cancer-related isoforms, ranging from 69.6 to 941 nM for hCA IX and 66.1 to 694 nM for isozyme XII (Table 1). Moreover, they demonstrated high selectivity versus hCAs IX and XI over hCAs I and II, being inactive at the highest concentration tested (10,000 nM).

- Interestingly, **UMB** (**1**) displayed a potent inhibitory activity and selectivity toward hCAs IX and XII, with K_I_ values of 24.9 and 45.1 nM, respectively, whereas its regioisomer **2** was less active.^43^
- Moreover, parent vanilloids **3–6** did not exhibit inhibitory activity against all four isoenzymes, with **Van** (**3**) showing weak activity on hCAs I and II, with K_I_ values of 11.4 and 7.15 μM, respectively. These data were also in accordance with phenolic compounds, usually recognized as weak inhibitors of CAs.
- Lastly, the propargylated **UMB** intermediate (**42**) was found to be totally inactive on isoenzymes I and II but showed interesting activity toward the cancer-related isoforms, with *K*_I_ values slightly higher than hybrids **7**–**24**, especially against hCA IX (*K*_I_ = 1350 nM), thereby presenting a good selectivity over the physiologically relevant isoforms I and II.^44^

By analyzing data for series *A* compounds, robust structure–activity relationship (SAR) considerations could be performed:

- Regarding hCA IX, the most potent compounds were **7**, **8**, **9**, and **10**, exhibiting *K*_I_ values ranging from 69.6 to 93.8 nM. These results underscore the importance of the spacer between **UMB** and the vanilloid nucleus in the activity, as a shorter linker correlates with a stronger inhibitory activity. In contrast, compounds **11** and **12**, featuring a 3-methylene linker and bearing ***o*** **Van** or **etVan** nucleus, respectively, showed reduced potency against hCA IX, with K_I_ values of 174.5 and 353 nM, respectively. Interestingly, these findings highlight the critical role of the vanilloid portion selection in designing effective hCA inhibitors. Compounds with longer spacers (4-, 5-, or 6-methylene units), as in hybrids **13**–**15** and **18**–**24**, showed *K*_I_ values of 434–829 nM, except **16** and **17**, which exhibited higher potency, with *K*_I_ values of 295 and 233 nM, respectively.
- Focusing our attention on hCA XII, compound **7** showed strong inhibition with a *K*_I_ of 66.1 nM, being the *best-in-class* of the series. Conversely, compounds **8**–**24** were less potent (*K*_I_ values of 358–694 nM). In this case, the distance between the two main moieties does not seem to significantly impact the inhibitory activity of the series A derivatives. Interestingly, compounds **16** and **17**, which showed potent inhibition of hCA IX, exhibited weak activity against hCA XII, with *K*_I_ values of 694 and 590 nM, respectively, thereby making them the least effective compounds overall against this isoform. These findings underscore the distinct structural requirements for these hybrids to inhibit the two tumor-associated hCA isoforms.

The introduction of the triazole ring in the spacer in the series *B* compounds (**25**–**33**) increased the distance between the **UMB** and vanilloid moieties, resulting in slightly different results for hCAs IX and hCA XII.

- In particular, series *B* compounds displayed a reduced inhibitory potency, with *K*_I_ values ranging from 627 to 941 nM against hCA IX.
- Better results were observed against hCA XII, with the compounds showing *K*_I_ values ranging from 225 to 450 nM. Notably, isomers **31** and **32** exhibited distinct inhibitory profiles: the ***iso*** **Van** derivative (**31**) was significantly more potent than the ***o*** **Van 32**, with half of the *K*_I_ value. This suggests that the position of the aldehyde and methoxy groups plays a key role in modulating hCA XII inhibition.

### 2.3 Molecular Modeling Studies

To evaluate the ligand–protein interactions that could rationalize the selectivity of the coumarin derivatives, molecular modeling analyses based on docking and molecular dynamics (MD) simulations were carried out. Compound **7** was selected as a representative ligand of the series due to its high inhibitory activity against both hCA IX and XII isoforms. Previous studies demonstrated that upon binding to the catalytic site of hCAs, coumarin-based ligands are subjected to hydrolysis at the level of their bicyclic core, which converts them into the corresponding 2-hydroxy-cinnamic acids. Nevertheless, the hydrolyzed compounds are still able to interact with the catalytic pocket of the enzymes, indeed representing the actual hCA inhibitors.^28,30^ Based on these considerations, molecular modeling studies were used to investigate the potential binding mode into the studied hCA isoforms of the 2-hydroxy-cinnamic acid obtained upon hydrolysis of compound **7**. The ligand was docked into the binding site of hCA I, II, IX, and XII using GOLD software with the PLP scoring function. Subsequently, MD simulations were performed to account for protein flexibility and consider the role of the solvent in the enzyme–ligand interactions (see Materials and Methods for details). Figure S1 shows the energy-minimized structures of the ligand–protein complexes obtained after the equilibration of the solvated systems, performed as a preliminary step in the MD simulations. As observed in Figure S1, the hydrolyzed form of compound **7** appeared to be able to bind to the catalytic site of all four hCA isoforms, forming water-mediated interactions between its carboxylic group and the prosthetic zinc ion of the enzymes. However, during the production stage of the MD simulation, performed for evaluating the stability of the equilibrated ligand–protein complexes, the interactions of **7** with the catalytic pocket of hCA I and II were progressively lost, and the ligand completely left the binding site of the two proteins within the first 50 ns of MD, as demonstrated by the analysis of the root-mean-square deviation (RMSD) of the position of the ligand during the simulation with respect to its initial coordinates (Figure S2). These results, suggesting that the ligand cannot properly interact with the catalytic site of hCAs I and II, are consistent with its lack of activity against hCAs I and II (Table 1). On the contrary, the binding disposition of **7** into the catalytic pocket of hCAs IX and XII was found to be stably maintained throughout 100 ns of simulations (Figure S2), in agreement with its activity and selectivity for the two isoforms (Table 1). With the aim of better evaluating and refining the binding mode of the inhibitor into the catalytic site of hCAs IX and XII, an additional 200 ns of MD simulation was performed for the two corresponding ligand–protein complexes (**7**-hCA IX and **7**-hCA XII). The RMSD analysis of the disposition of the ligand during the whole simulation time (300 ns) confirmed the stability of the ligand–protein interactions observed in the two complexes, although the compound showed considerable adjustments of its binding conformation during the MD, compared to the initial conformation, with an average RMSD of about 4 Å (Figure S3). Interestingly, after about 120 ns of MD, the binding conformation of the ligand into hCA IX partially rearranged (as shown by the increase in the average RMSD to 5.7 Å) to significantly converge into the binding mode observed in complex with hCA XII, and the optimized conformation was then maintained for the remaining simulation time (Figure S3). Figure 3 shows the minimized average structure of hCAs XI and XII in complex with the hydrolyzed form of compound **7** obtained from the second half of the extended MD simulation.

**Figure 3:**
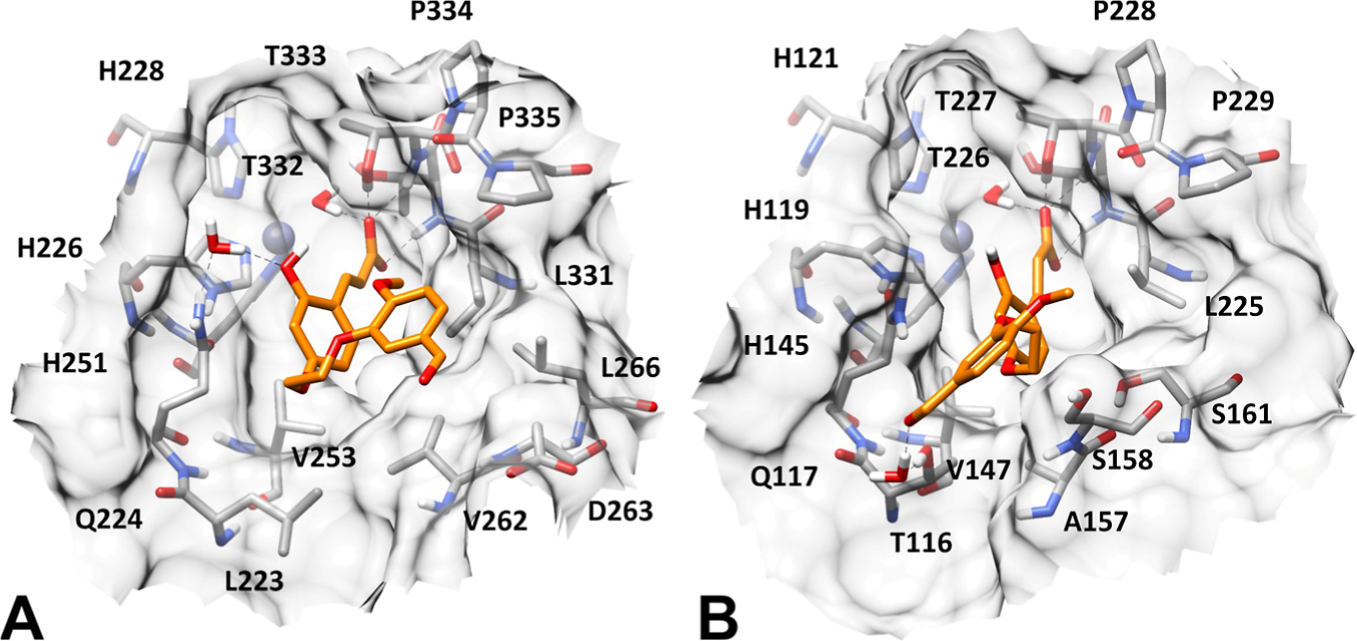
Minimized average structure of compound **7** in its hydrolyzed form (orange) in complex with hCA IX (**A**) and hCA XII (**B**). In all panels, the surface of the protein binding site is shown in transparency.

As anticipated, the binding modes of the ligand into the two hCA isoforms appear similar, at least at the level of the 2-hydroxy-cinnamic acid moiety. In fact, the ligand is anchored to both enzymes’ catalytic pockets thanks to its carboxylic group, which forms three different H-bonds with the two threonine residues placed in proximity of the prosthetic zinc ion. In particular, two H-bonds are established with the backbone nitrogen and the hydroxyl group of T333 in hCA IX (Figure 3A) and the homologue T227 in hCA XII (Figure 3B), while a third H-bond is formed with the backbone nitrogen of T332 in hCA IX (Figure 3A) and the homologue T226 in hCA XII (Figure 3B). Moreover, the ligand shows water-mediated interactions with the hydroxyl group of T332/T226 and the catalytic ion of both hCA isoforms. Finally, even the main hydrophobic interactions formed by the 2-hydroxy-cinnamic portion of the inhibitor in hCAs IX and XII are comparable, being represented by lipophilic contacts with V235 and L331 in hCA IX (Figure 3A), and with homologue residues V147 and L225 in hCA XII (Figure 3B). In addition to these ligand–protein interactions, shared by the two complexes, in hCA IX, the hydrolyzed form of compound **7** shows a further water-mediated H-bond with the side chain of Q224, established by its phenolic group, as well as hydrophobic interactions with V262 and L266 formed by its vanilloid moiety (Figure 3A). The lack of such interactions in hCA XII is well compensated by the presence of a water-bridged H-bond with T116 and van der Waals interactions with A157, S158, and S161, which are both formed by the ligand’s vanilloid core (Figure 3B). Overall, the similar binding modes predicted for the compound in hCA IX and XII are consistent with the similar inhibitory activities determined for **7** against the two enzymes. Moreover, the presence of a water-mediated H-bond between the terminal aldehyde group of the ligand and T116 might justify the drop of hCA XII inhibitory activity observed when moving such a group from the *meta* to *para* position with respect to the alkoxy linker, as in compound **8**, and/or when adding a further methylene group to the linker connecting the vanilloid moiety with the coumarin core, as in compounds **9** and **10** (Table 1). In addition, the evidence that compound **8** maintains the same hCA IX inhibitory activity observed for **7**, and that **9** and **10** only show a minor potency reduction against this isoform (Table 1), is in agreement with the interactions predicted for the vanilloid moiety of compound **7** within hCA IX, consisting only in van der Waals interactions that are less easily perturbed by the structural changes present in compounds **8–10** compared to **7**.

Finally, the analysis of the above-described ligand–protein complexes, *i.e.,* **7**-hCA IX and **7**-hCA XII, in comparison with the structures of hCA I and hCA II binding sites, suggested a rationale for justifying why the ligand would not be able to properly interact with the catalytic pockets of these latter isoforms. As shown in Figure S4A,B, in hCA I, the carboxylic group of the ligand cannot form the two anchoring H-bonds established with T333 in hCA IX (Figure 3A) and the homologue T227 in hCA XII (Figure 3B), due to the presence of a histidine (H201 in hCA I) in place of the threonine residues. Moreover, the presence in hCA I of four nonconserved residues (*i.e.,* F92, L132, A133, and A136) in the region of the binding pocket interacting with the vanilloid moiety of **7** would not let the ligand to assume a binding mode similar to that predicted into either hCAs XI (Figure 3A) or XII (Figure 3B) and thus to maintain the same water-mediated interactions. Although both the threonine residues anchoring the carboxylic group of **7** to hCA IX and XII binding sites are conserved in hCA II, four nonconserved residues (*i.e.,* I91, F130, G131, and V134) substantially reshape the entrance of the catalytic pocket of this isoform (Figure S4C,D). In particular, the side chain of F130 creates a sort of hill in the middle of the cavity entrance, which would significantly clash with both the vanilloid moiety and the cinnamic ring of **7**, in its binding mode predicted into hCA IX (Figure S4C). For this reason, the ligand could maintain neither a similar binding conformation nor the water-bridged H-bond with Q224 observed in hCA IX (Figure 3A). Similarly, the steric clashes with F130 would hamper the ligand from assuming the binding conformation predicted in hCA XII (Figure S4D); in addition, the presence of I91 in hCA II, replacing T116 of hCA XII, would certainly prevent the formation of the water-mediated H-bond formed by the vanilloid group of **7** in hCA II (Figure 3B). Overall, these considerations may justify the lack of stable interaction between compound **7** and hCAs I/II, and thus, the lack of inhibitory activity of the ligand against the two isoforms.

### 2.4 In Vitro Investigations on Human Cell Lines

#### 2.4.1 Cell Viability Assessment

The effects of **UMB** (**1**), parent vanilloids (**3**–**6**), and the most potent hybrids (**7**, **8**, **9**, **10**, **11**, **26**, and **33**) were assessed on human bronchial epithelial (BEAS-2B) and human lung adenocarcinoma (A549) cell lines by measuring their viability in response to increasing concentrations of compounds up to 72 h via the MTT assay (Figure 4).

**Figure 4:**
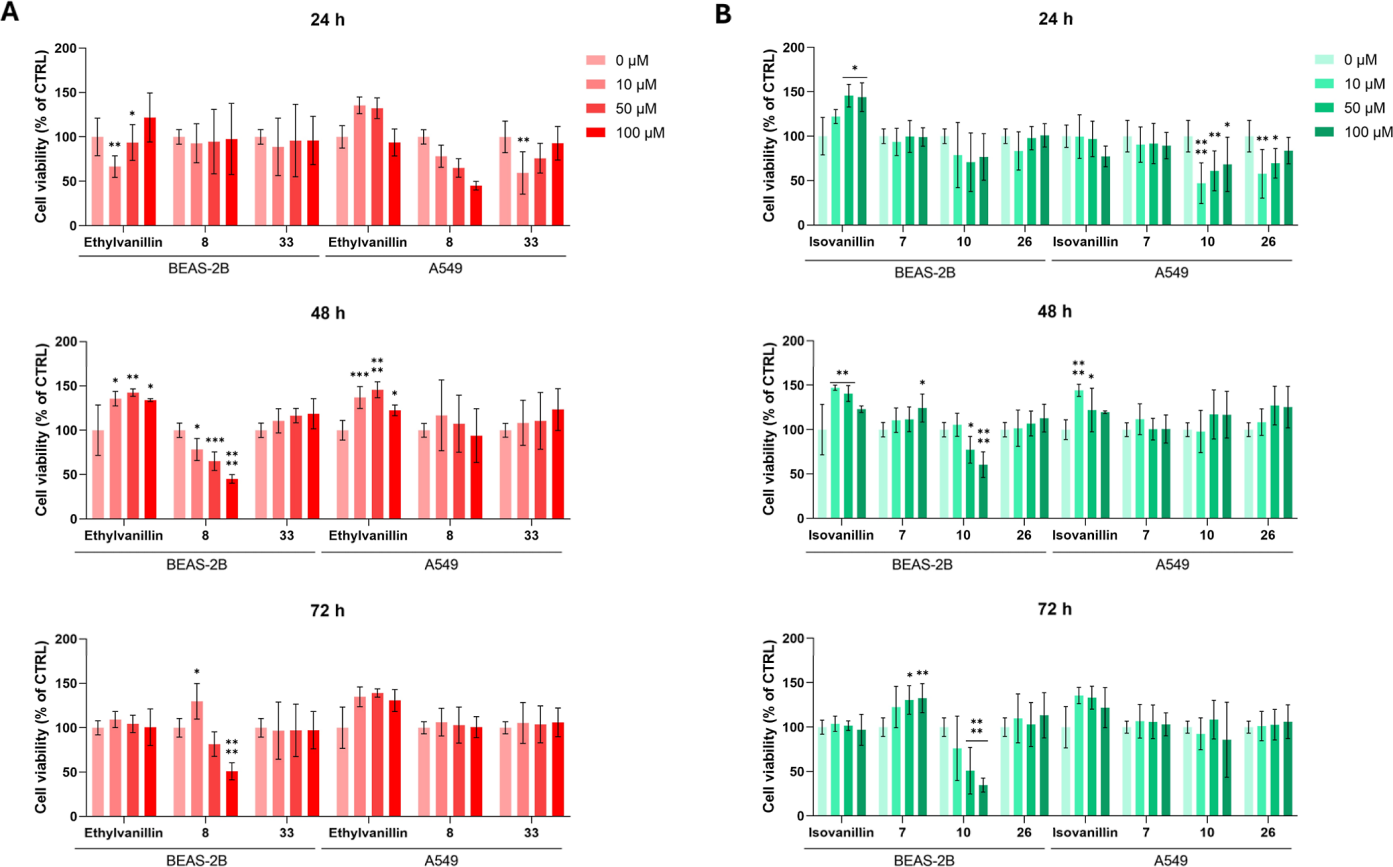
Cell viability of BEAS-2B and A549 cells exposed to increasing concentrations (0–100 μM) of **etVan** and **etVan**-derived compounds (A), ***iso*** **Van** and ***iso*** **Van**-derived compounds (B), for 24, 48, and 72 h. The bar graphs represent cell viability percentages. The untreated control (CTRL = 0 μM) is set as 100%. Data are presented as means ± standard deviations obtained from two independent experiments in triplicate (*n* = 6). * = *p* < 0.01, ** = *p* < 0.001, *** = *p* < 0.0001, **** = *p* < 0.00001 comparing treated to the untreated control.

Interestingly, **etVan** (**6**) significantly reduces BEAS-2B cell viability within the first 24 h in an inverse dose-dependent manner, while it promotes cell growth at later time points in both BEAS-2B and A549 cells (Figure 4A). These data were compared to those obtained by testing two **etVan**-bearing hybrids from the series *A* (**8**) and *B* (**33**), showing distinct activity profiles. Compound **8** induces a minor decrease in BEAS-2B cell vitality at the earliest exposure time. Next, the activity becomes dose-dependent, BEAS-2B cell vitality being significantly reduced to 50% at the highest concentration (100 μM) tested compared to that of the untreated control. In parallel, **8** impairs A549 cell growth exclusively within the first 24 h of exposure. Otherwise, compound **33** has no relevant effects on the viability of BEAS-2B cells. However, it decreases A549 cell survival in an inverse dose-dependent manner, specifically in the first 24 h after exposure.

The focused **etVan** subseries tested reveals intriguing and divergent biological effects, particularly in relation to time- and cell-type-specific responses. While the parent **etVan** showed time-dependent dual activity, its **UMB**-hybridization products **8** and **33** exhibited distinct and more selective cytotoxic profiles against the cancer cell line, highlighting their promising role as anticancer agents.

As regards ***iso*** **Van** subseries, the parent compound was tested along with the alkyl-linker-based hybrids **7** and **10** and the triazole derivative **26**. BEAS-2B cell viability rises dose-dependently up to 24 h following ***iso*** **Van** (**4**) treatment. After that, it decreases dose-dependently, although it is still higher than the control sample (Figure 4B). In contrast, A549 cells grow significantly after 48 h of exposure. Compound **7** decreases both BEAS-2B and A549 cell viability exclusively within 24 h of exposure. Compound **10** significantly impairs BEAS-2B cell survival after 48 h in a dose-dependent manner. Conversely, in A549 cells, it demonstrates an inverse dose-dependent activity exclusively within the first 24 h, as well as compound **26**, showing selectivity for A549 cells. As regards the ***iso*** **Van** subseries, the parent ***iso*** **Van** (**4**) mainly promotes cell viability, whereas hybrid **7** has early effects on cell viability in both cell lines, and compounds **10** and **26** are selectively cytotoxic toward BEAS-2B and A549 cells, respectively.

As regards **Van** compounds, we found that **Van** stimulates the metabolic activity of BEAS-2B and A549 cells at all time points, except for nonpathological cells at 72 h (Figure 5A). Similarly, **Van**-hybrid **9** significantly increases BEAS-2B cell viability after 48 h of exposure but has no relevant activity on A549 cells, except at 24 h, where it slightly reduces cell survival. Also, we compared the *o* **Van** parent compound to its hybrid **11**. BEAS-2B cell vitality is significantly enhanced within the first 24 h of exposure to ***o*** **Van**, followed by a time- and dose-dependent reduction in cell viability. In contrast, ***o*** **Van** consistently enhances the A549 cell viability across all time points analyzed (Figure 5B). Similarly, compound **11** impairs BEAS-2B cell viability in a time- and dose-dependent manner at 48 and 72 h, while in A549 cells, it reduces viability only at 24 h. Interestingly, **UMB** (**1**) appears to be active at 24 h, decreasing the viability of both BEAS-2B and A549 cells, except at 50 μM on BEAS-2B cells (Figure 5C). However, after 48 and 72 h, cell viability is slightly higher than or lower than control levels.

**Figure 5:**
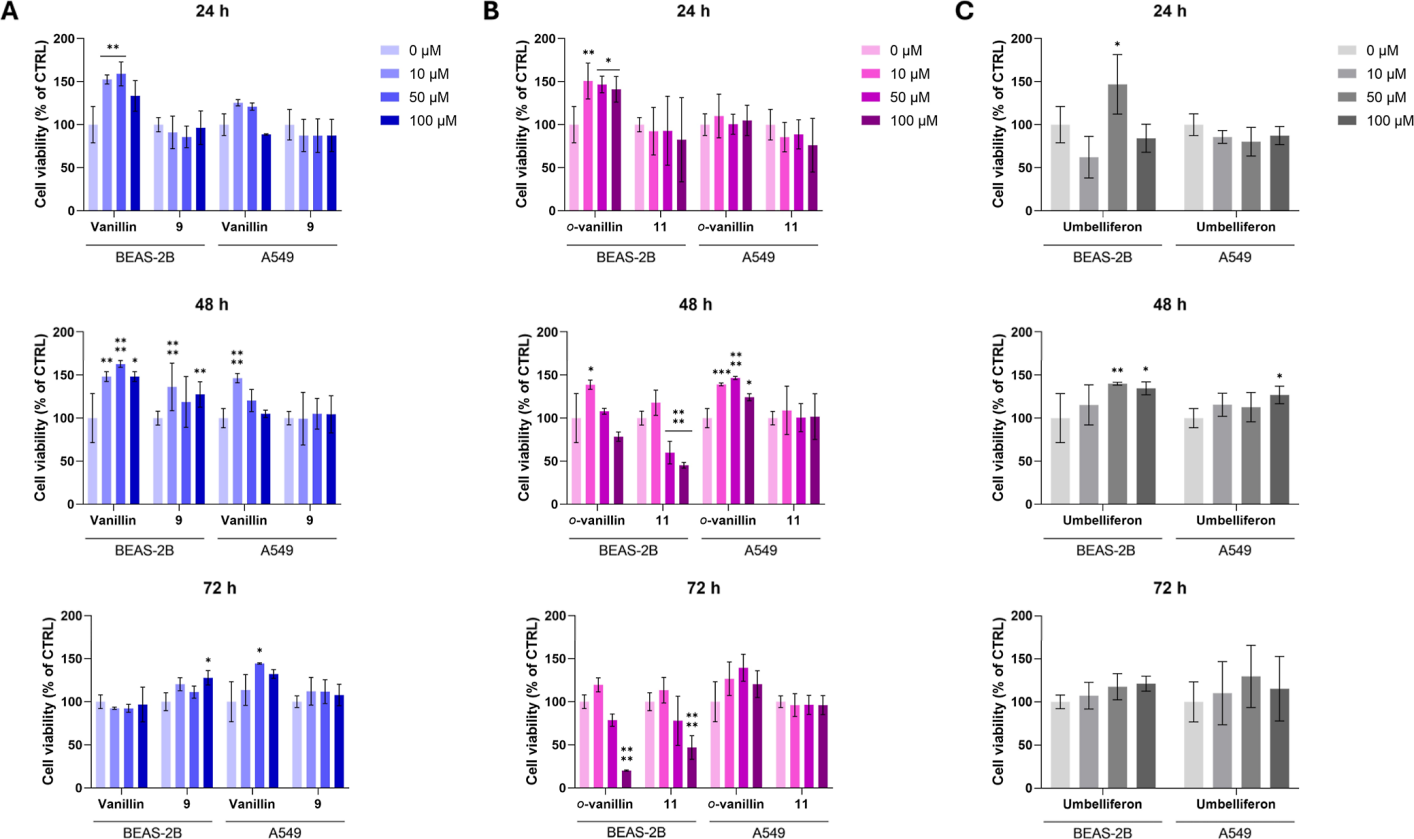
Cell viability of BEAS-2B and A549 cells exposed to increasing concentrations (0–100 μM) of **Van** and **Van**-derived compounds (**A**), ***o*** **Van** and ***o*** **Van**-derived compounds (**B**), and **UMB** for 24, 48, and 72 h (**C**). The bar graphs represent cell viability percentages. The untreated control (CTRL = 0 μM) is set as 100%. Data are presented as means ± standard deviations obtained from two independent experiments in triplicate (*n* = 6). * = *p* < 0.01, ** = *p* < 0.001, *** = *p* < 0.0001, **** = *p* < 0.00001 comparing treated to the untreated control.

In summary, the hybridization of **UMB** with vanilloids seemed to significantly modulate the individual biological activities of parent compounds, often enhancing selectivity on A549 cells over that of BEAS-2B cells. We found that the parent compound **UMB** generally promotes cell viability in both cell lines, often exhibiting dual time-dependent effects, whereas the tested hybrids determined more specific and selective effective profiles, particularly against the A549 cancer cell line. Thus, the structural modification performed was able to boost the effect of the hydroxylated compounds **UMB** and vanilloids to more potent hCA inhibitors, highlighting their potential as promising candidates for therapeutic development in hypoxic tumors.

#### 2.4.2 Cytotoxicity Occurrence in A549 Cells Exposed to Selected Compounds (LDH Assay)

Then, compounds **9**, **26**, and **33** were selected because of their biocompatibility on BEAS-2B and effectiveness on A549, especially within the first 24 h. To assess whether the decrease in viability might be a consequence of cytotoxicity occurrence in the presence of selected compounds, the amount of lactate dehydrogenase (LDH) released by A549 cells was measured in response to increasing concentrations of compounds after a 24 h exposure (Figure 6). Hybrids **26** and **33** at lower concentrations (10 μM) are the most effective in causing cytotoxicity in an inverse dose-dependent manner, in alignment with data on cell viability. The release of LDH into the culture medium is nearly doubled compared to that of the control sample, with 1.71- and 1.80-fold increases for **26** and **33**, respectively. In contrast, compound **9** exhibits greater cytotoxicity at higher concentrations. However, none of the LDH increases registered are statistically significant. Hence, cell cycle analyses were further performed in the same experimental conditions to investigate if the decrease of cell viability in the presence of **9**, **26,** and **33** might be caused by an arrest of cell proliferation due to a cell block in one of the cell cycle checkpoints.

**Figure 6:**
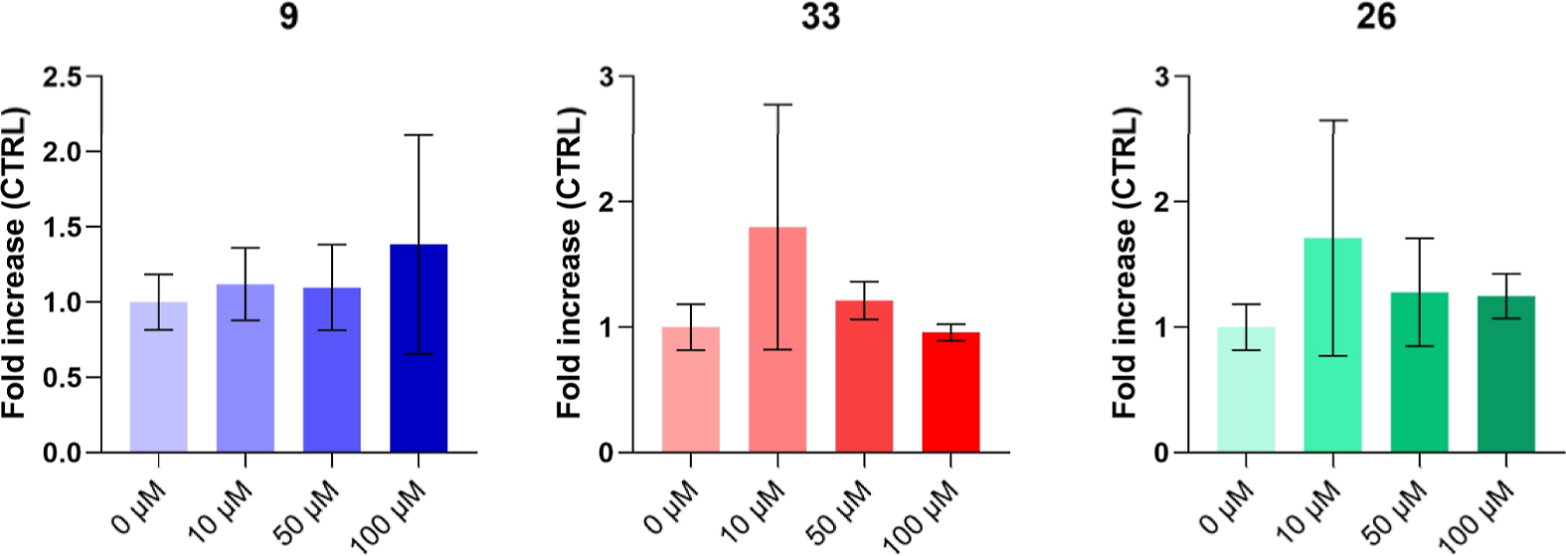
Cytotoxicity occurrence in A549 cells exposed to increasing concentrations (0–100 μM) of selected compounds (**9**, **26**, and **33**) after 24 h. The bar graphs show the amount of lactate dehydrogenase (LDH) released from treated A549 cells as the fold increase over that secreted by untreated cells (CTRL = 0 μM) after 24 h of exposure. Bars show mean values ± standard deviations summarized from individual values in independent experiments (*n* = 4).

#### 2.4.3 Cell Cycle Analyses

To establish whether the selected compounds **9**, **26**, and **33** could induce cell cycle arrest at specific checkpoints and decrease A549 cell proliferation, cell percentages found in G1, S, and G2 phases (Figure 7) were analyzed after 6 and 24 h from treatment. In Figure 7, the bars related to the control sample display a typical cell cycle profile, with proliferative and active cells at 6 h (G1 phase = 51.95%; S phase = 30.56%; G2/M phase = 17.49%) followed by an increase in cells in the S phase (37.00%) and a decrease in cells in the G2 phase (11.16%) at 24 h. The G1 phase does not vary. Treatment with compound **9** leads to a significant increase in G1 cells (54.25% at 50 μM) and a minor decrease in G2 cells (14.04% at 50 μM) after 6 h, followed by a moderate reduction in G1 cells after 24 h. Exposure to compound **33** at higher concentrations (50 and 100 μM) results in a significantly higher percentage of G1 cells compared with the control (54.80%), with a decrease in G2 cells (13.44% at 100 μM). The G1 cell percentage falls after 24 h, showing a dose-dependent behavior along with a rise in S-phase cells. Compound **26** causes a remarkable delay in the cell cycle in the G1 phase (56.17% at 100 μM) along with a decrease in G2 cells after 6 h. Subsequently, at 24 h, the G1 cell percentage decreases, while the S phase cell percentage increases.

**Figure 7:**
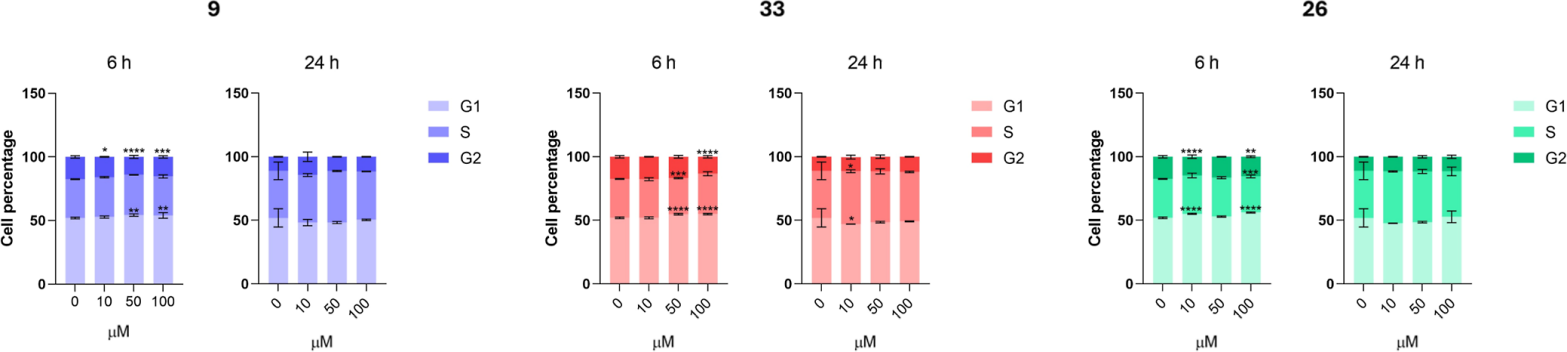
Cell cycle analysis in A549 cells exposed to increasing concentrations (0–100 μM) of selected compounds (**9**, **33**, and **26**) after 6 and 24 h. Data are presented as means ± standard deviations from three independent experiments (*n* = 3). Bars highlight cell percentages in the various phases of the cell cycle (G1, S, and G2) of A549. * = *p* < 0.01, ** = *p* < 0.001, *** = *p* < 0.0001, and **** = *p* < 0.00001 comparing treated to the untreated control.

The observed changes in cell cycle progression upon treatment with hybrids **9**, **26**, and **33** clearly highlight the compounds’ ability to affect cell proliferation by inducing cell cycle arrest, particularly in the G1 phase. Indeed, such an increase in G1 cells can indicate a delay in the cycle progression, while a decrease in G2 cells can correspond to an inhibition of mitosis.^45^

#### 2.4.4 Influence on CD9 and EPCAM Expression

The effect of compounds **9**, **33**, and **26** at increasing concentrations (10, 50, and 100 μM) on A549 cells was evaluated after 24 h of exposure by measuring the expression of cell surface markers CD9 and epithelial cell adhesion molecule (EPCAM) using specific fluorescently labeled antibodies and DAPI staining. Briefly, CD9 is a tetraspanin mainly involved in cell migration and metastasis.^46^ In parallel, the transmembrane glycoprotein EPCAM is involved in cell adhesion and is a well-known cell biomarker of lung cancer.^47^

The fluorescence microscopy images shown in Figure 8A,C indicate that all three compounds produced a qualitatively similar pattern in the expression of the analyzed markers. No appreciable differences were observed between the compounds themselves; however, the concentration had a significant impact on the variability of the CD9 fluorescence intensity, suggesting a dose-dependent response for this marker. Interestingly, and keeping in consideration the role of CD9 in reducing migration of A549 cells, compound **33** revealed the strongest effect, significantly decreasing the CD9 fluorescence intensity at 100 μM compared to control and 10 μM. Compound **26** significantly decreases CD9 expression at 100 μM compared to 10 μM (Figure 8B). Similarly, the compounds reveal the same effect on the EPCAM fluorescence intensity, although the *post hoc* analysis failed to find significance for multiple comparisons.

**Figure 8:**
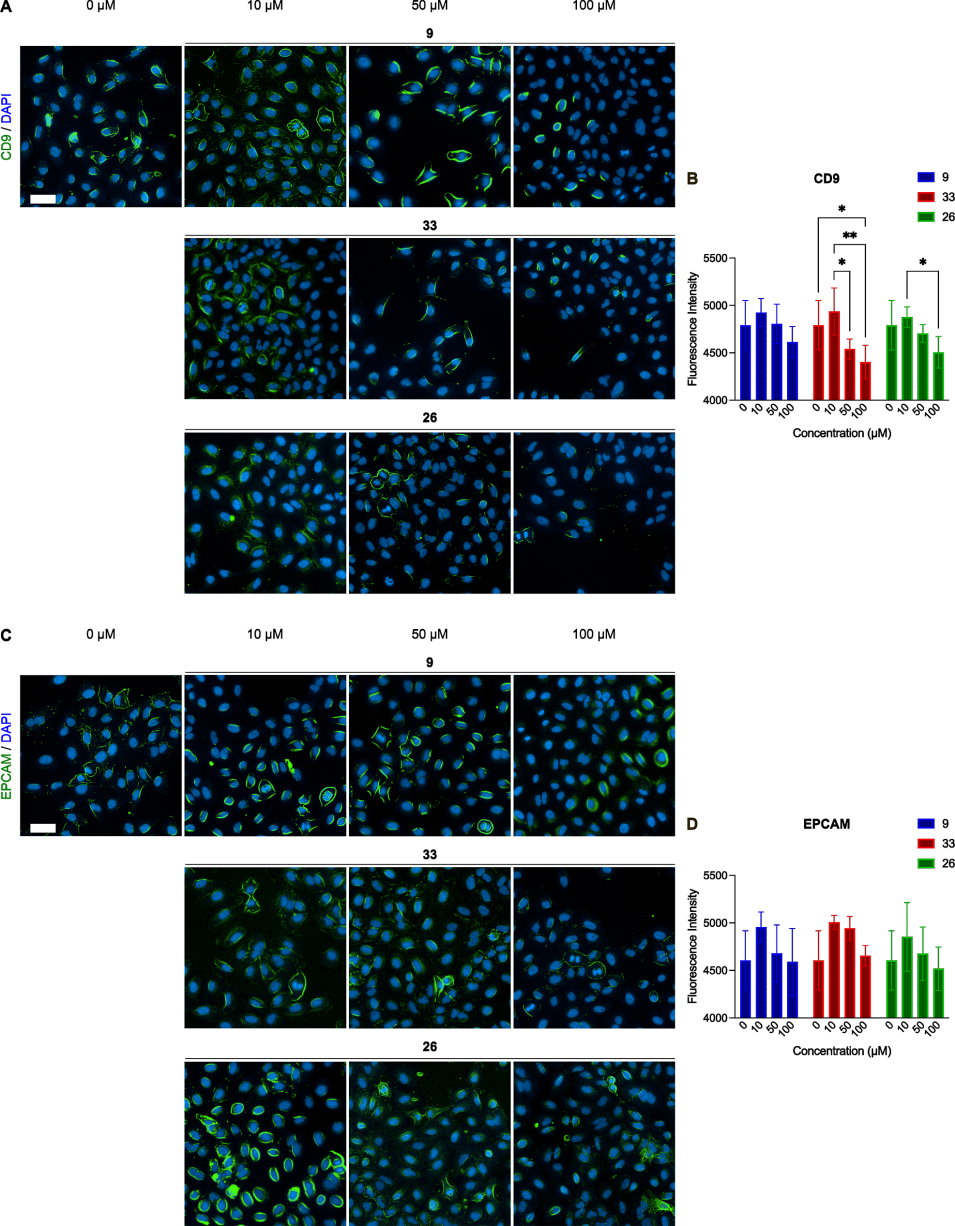
(A,C) Representative images of CD9 and EPCAM expression following the different treatments with selected compounds (**9**, **33**, and **26**). (B) Histograms and statistical analysis results of CD9 fluorescence intensity. (D) Histograms and statistical analysis results of EPCAM fluorescence intensity. Values are expressed as mean and standard deviation (*n* = 4); * = *p* < 0.05, ** = *p* < 0.01. Scale bar 100 μm, captured at 20×.

#### 2.4.5 Anti-inflammatory Response in BEAS-2B Cells

Exposure of BEAS-2B cells to the conditioned medium from lipopolysaccharide (LPS)-stimulated macrophages led to a time-dependent decrease in viability (Figure 9). Notably, a significant reduction, approximately 50% compared to the control, was observed after 48 h of treatment. In contrast, no significant changes in the metabolic activity were detected within the first 24 h of exposure. While conditioned, treating cells with selected compounds **9**, **33**, and **26** effectively counteract the conditioned medium-induced reduction in viability. Notably, compounds can restore viability to levels comparable to those of untreated controls. Among them, compounds **9** and **33** demonstrate higher potency than **26**, exhibiting significant efficacy even at lower concentrations (10 and 50 μM).

**Figure 9:**
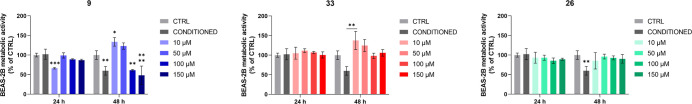
Cell viability of conditioned BEAS-2B cells exposed to increasing concentrations (0–150 μM) of selected compounds (**9**, **33**, and **26**) for 24 and 48 h. The bar graphs represent cell viability percentages. The untreated control (CTRL = 0 μM) is set as 100%. Data are presented as means ± standard deviations obtained from one experiment in triplicate (*n* = 3). * = *p* < 0.01, ** = *p* < 0.001, *** = *p* < 0.0001, **** = *p* < 0.00001 comparing treated to the untreated control.

Next, the LDH assay was performed on the supernatants collected from BEAS-2B cells exposed to the conditioned medium and to the compounds (Figure 10A). As expected, a significant increase in LDH release is observed after 48 h of treatment. Consistent with the MTT assay results, all the compounds reduce the LDH release in a dose-dependent manner, effectively counteracting the cytotoxic effects of the conditioned medium. Microscopic analysis of BEAS-2B cells exposed to the conditioned medium revealed marked morphological alterations in accordance with an inflammatory and cytotoxic response (as indicated by the red arrows; Figure 10B). Indeed, cells appear more round-shaped and with enhanced cytoplasmic elongations, whereas they display the typical polygonal morphology of epithelial cells in the untreated control. After 48 h of treatment, cells exhibited a loss of their typical epithelial morphology, characterized by a uniform monolayer of polygonal cells with well-defined borders and intact cell–cell junctions. It was observed that there was a reduction in cell density and loss of intercellular contacts. Treatment with selected compounds **9**, **33**, and **26** was effective in restoring the disrupted epithelial integrity induced by the conditioned medium, with a full recovery of normal cell shape and adhesion. Interestingly, these findings seem to suggest that the tested compounds **9**, **33**, and **26** could exert a cytoprotective effect against inflammation-induced cellular damage in BEAS-2B cells, and, in particular, **9** and **33** demonstrate anti-inflammatory properties at lower concentrations.

**Figure 10:**
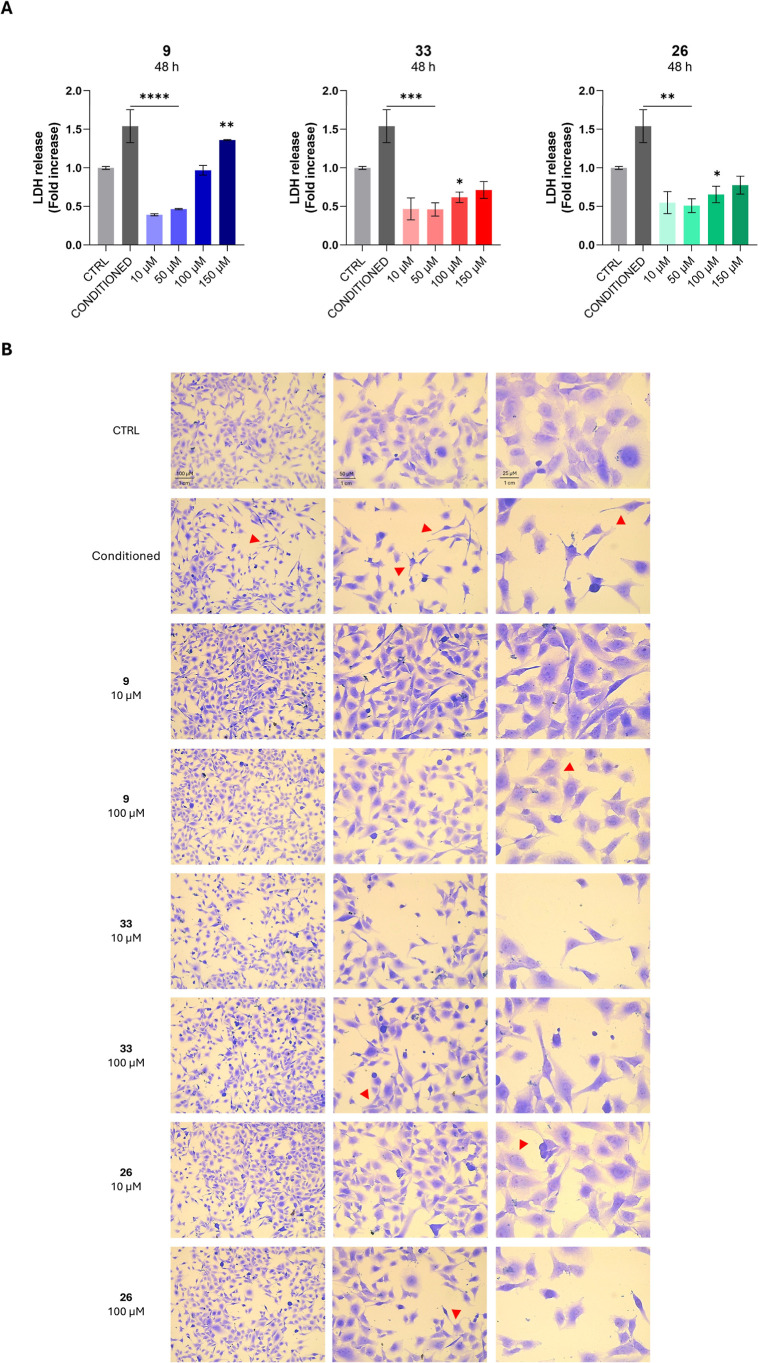
Cytotoxicity occurrence in BEAS-2B cells exposed to increasing concentrations (0–150 μM) of selected compounds (**9**, **33**, and **26**) after 48 h (**A**). The bar graphs show the amount of lactate dehydrogenase (LDH) released from treated BEAS-2B cells as the fold increase on untreated cells (CTRL = 0 μM) after 48 h of exposure. Bars show mean values ± standard deviations summarized from individual values in independent experiments (*n* = 4). Microscopic images of crystal violet-stained BEAS-2B cells exposed to the conditioned medium after 48 h, captured at 10×, 20×, and 40× magnifications (**B**).

## 3 Conclusions

In summary, we developed a series of **UMB**-vanilloid hybrids as selective inhibitors of the tumor-associated hCAs IX and XII. Most derivatives displayed potent and isoform-specific inhibitory profiles and demonstrated significant anticancer and anti-inflammatory activities in vitro. Interestingly, they were able to induce G1 arrest, downregulate metastasis-related markers, and protect nonmalignant bronchial epithelial cells from inflammatory stress. Their dual role as anticancer and cytoprotective agents underscores the potential of this hybridization approach, enabling the generation of multifunctional scaffolds for NSCLC therapy and laying the foundation for further.

## 4 Experimental Section

### 4.1 Synthesis of the Derivatives Library

#### 4.1.1 General Chemistry

All commercially available chemicals and solvents were used as purchased. Chromatographic separations were performed on columns packed with silica gel (230–400 mesh, for the flash technique). Reaction monitoring was performed through thin-layer chromatography (TLC) by using 0.2 mm-thick silica gel–aluminum-backed plates (60 F254). TLC spot visualization was performed under short and long wavelengths (254 and 365 nm, respectively) of ultraviolet irradiation and stained with ninhydrin or basic permanganate. ^1^H and ^13^C NMR spectra were recorded on a Varian Oxford 300 MHz NMR operating at 300 and 75 MHz, respectively. The NMR spectra for the newly synthesized compounds are reported in the Supporting Information, in Figures S5–S58. Spectra are reported in parts per million (δ scale) and internally referenced to the CDCl_3_ or DMSO-*d*_6_ signal, at δ 7.26 and 2.50 ppm, respectively. Data are shown as follows: chemical shift, multiplicity (s = singlet, d = doublet, t = triplet, q = quartet, qi = quintet, m = multiplet, br = broad signal, and ap = apparent), integration, and coupling constants (*J*) in Hertz (Hz). Chemical shifts for carbon are reported in parts per million (δ scale) and referenced to the carbon resonances of CDCl_3_ or DMSO-*d*_6_ at δ 77.0 or 39.0 ppm, respectively. The ^1^H NMR spectra confirmed the anticipated number of hydrogens in each compound. Melting points were measured on a Stuart melting point apparatus SMP1 (Fisher Scientific Italia, Segrate (MI), Italy) and are uncorrected (temperatures are reported in °C). Elemental analyses for C, H, and N were recorded on a PerkinElmer 240 B microanalyzer (PerkinElmer Italia, Milan, Italy), and the analytical results are within ±0.4% of the theoretical values for all compounds (purity >95%). Microwave-assisted reactions were performed with Biotage Initiator+ (Biotage Sweden AB, Uppsala, Sweden) in a 10 mL vial suitable for an automatic single-mode reactor (2.45 GHz high-frequency microwaves, power range 0–300 W). The internal vial temperature was controlled by an IR sensor.

#### 4.1.2 Procedure and Characterization Data for Derivatives **7–42**

##### 4.1.2.1 General Procedure for the Synthesis of Compounds **7–24** (Series A)

Bromo intermediates **34–37**. The proper alkyl halide selected from 1-bromo-2-chloroethane, 1-bromo-3-chloropropane, 1,4-dibromobutane, 1,5-dibromopentane, and 1,6-dibromohexane (1.5 equiv) was added to the colorless solution of the suitable vanilloid (**3–6**) (1.0 equiv) in ethanol (0.4 M). Then, an aqueous solution of K_2_CO_3_ (4.0 equiv, 1.0 M) was added to the stirring reaction mixture, and the final homogeneous solution was placed in a microwave reactor and exposed to microwave irradiation at 120 °C for 2–3 h (irradiation power reaches its maximum at the beginning of the reaction; then it decreases to lower and quite constant values). After the reaction had reached completion, the reaction mixture was cooled with pressurized air, poured into cold H_2_O, and extracted with EtOAc three times. The combined organic layers were dried over anhydrous Na_2_SO_4_, filtered, and evaporated under reduced pressure. The reaction crude was then purified through flash column chromatography on silica gel with a suitable mixture of *n*-hexane/EtOAc to afford the intermediates **34–37**. Experimental data were in agreement with the literature.

Alkyl Hybrids **7–24**. The suitable bromo derivative **34–37** (1.5 equiv) was dissolved in DMF (0.4 M). Then, **UMB** (**1**) (1.0 equiv) and K_2_CO_3_ (4.0 equiv) were added to the stirring reaction mixture and placed in a microwave reactor and exposed to microwave irradiation at 100 °C for 2 h. Once the reaction was completed, the mixture was poured into H_2_O and extracted with EtOAc three times (3 × 15 mL). The combined organic layers were dried over Na_2_SO_4_, filtered, and evaporated under reduced pressure to afford the crude products. These were then subjected to column chromatography on silica gel with different *n*-hexane/EtOAc mixtures and recrystallization from ethanol (when solid) to afford compounds **7**–**24**.

4-Methoxy-3-(2-((2-oxo-2*H*-chromen-7-yl)­oxy)­ethoxy)­benzaldehyde **(7)**. White solid, mp 148–150 °C, 43% yield. ^**1**^ **H NMR** (300 MHz, CDCl_3_) δ: 3.96 (s, 3H, OCH_3_), 4.45–4.47 (m, 4H, OCH_2_CH_2_O), 6.26 (d, *J* = 9.3 Hz, 1H, Ar), 6.89–6.94 (m, 2H, Ar), 7.01 (d, *J* = 9.0 Hz, 1H, Ar), 7.38 (d, *J* = 8.4 Hz, 1H, Ar), 7.49–7.51 (m, 2H, Ar), 9.86 (s, 1H, CHO). ^**13**^ **C NMR** (75 MHz, CDCl_3_) δ: 56.2, 67.0, 67.4, 101.8, 110.9, 111.0, 112.9, 113.1, 113.4, 127.5, 128.8, 130.0, 143.4, 148.5, 155.0, 155.8, 161.2, 161.8, 190.7. Anal. Calcd for C_19_H_16_O_6_: C, 67.05; H, 4.74. Found: C, 67.25; H, 4.82.

3-Ethoxy-4-(2-((2-oxo-2*H*-chromen-7-yl)­oxy)­ethoxy)­benzaldehyde **(8)**. White solid, mp 139–141 °C, 67% yield. ^**1**^ **H NMR** (300 MHz, CDCl_3_) δ: 1.44 (t, *J* = 7.0 Hz, 3H, OCH_2_CH_3_), 4.14 (q, *J* = 7.0 Hz, 2H, OCH_2_CH_3_), 4.44–4.50 (m, 4H, OCH_2_CH_2_O), 6.26 (d, *J* = 9.3 Hz, 1H, Ar), 6.89–6.91 (m, 2H, Ar), 7.05 (d, *J* = 7.5 Hz, 1H, Ar), 7.37–7.46 (m, 3H, Ar), 7.63 (d, *J* = 9.3 Hz, 1H, Ar), 9.85 (s, 1H, CHO). ^**13**^ **C NMR** (75 MHz, CDCl_3_) δ: 14.6, 64.6, 67.0, 67.6, 101.8, 111.2, 112.9, 113.0, 113.4, 126.1, 128.8, 130.8, 143.3, 149.5, 153.6, 155.8, 161.0, 161.7, 190.9. Anal. Calcd for C_20_H_18_O_6_: C, 67.79; H, 5.12. Found: C, 67.96; H, 5.20.

3-Methoxy-4-(3-((2-oxo-2*H*-chromen-7-yl)­oxy)­propoxy)­benzaldehyde **(9)**. White solid, mp 135–137 °C, 80% yield. ^**1**^ **H NMR** (300 MHz, CDCl_3_) δ: 2.35–2.43 (m, 2H, OCH_2_ CH_2_CH_2_O), 3.92 (s, 3H, OCH_3_), 4.25 (t, *J* = 5.8 Hz, 2H, OCH_2_CH_2_CH_2_O), 4.31 (t, *J* = 6.1 Hz, 2H, OCH_2_CH_2_ CH_2_O), 6.22 (d, *J* = 9.3 Hz, 1H, Ar), 6.82–6.86 (m, 2H, Ar), 7.00 (d, *J* = 8.4 Hz, 1H, Ar), 7.34–7.45 (m, 3H, Ar), 7.63 (d, *J* = 9.6 Hz, 1H, Ar), 9.84 (s, 1H, CHO). ^**13**^ **C NMR** (75 MHz, CDCl_3_) δ: 28.8, 56.0, 64.8, 65.3, 101.5, 109.4, 111.6, 112.7, 112.8, 113.2, 126.6, 128.8, 130.3, 143.3, 149.9, 153.7, 155.9, 161.1, 161.9, 190.8. Anal. Calcd for C_20_H_18_O_6_: C, 67.79; H, 5.12. Found: C, 67.53; H, 5.01.

4-Methoxy-3-(3-((2-oxo-2*H*-chromen-7-yl)­oxy)­propoxy)­benzaldehyde **(10)**. White solid, mp 141–142 °C, 77% yield. ^**1**^ **H NMR** (300 MHz, CDCl_3_) δ: 2.32–2.40 (m, 2H, OCH_2_ CH_2_CH_2_O), 3.95 (s, 3H, OCH_3_), 4.25 (q, *J* = 7.2 Hz, 4H, OCH_2_CH_2_ CH_2_O), 6.23 (d, *J* = 9.3 Hz, 1H, Ar), 6.84–6.87 (m, 2H, Ar), 6.98 (d, *J* = 8.1 Hz, 1H, Ar), 7.36 (d, *J* = 9.0 Hz, 1H, Ar), 7.45 (d, *J* = 9.3 Hz, 2H, Ar), 7.62 (d, *J* = 9.3 Hz, 1H, Ar), 9.83 (s, 1H, CHO). ^**13**^ **C NMR** (75 MHz, CDCl_3_) δ: 28.8, 56.2, 64.9, 65.2, 101.3, 110.0, 110.5, 110.7, 112.5, 113.1, 113.2, 127.1, 128.7, 130.0, 143.4, 148.8, 154.9, 155.9, 161.2, 162.1, 190.9. Anal. Calcd for C_20_H_18_O_6_: C, 67.79; H, 5.12. Found: C, 67.56; H, 5.19.

3-Methoxy-2-(3-((2-oxo-2*H*-chromen-7-yl)­oxy)­propoxy)­benzaldehyde **(11)**. White solid, mp 94–96 °C, 85% yield. ^**1**^ **H NMR** (300 MHz, CDCl_3_) δ: 2.28–2.26 (m, 2H, OCH_2_ CH_2_CH_2_O), 3.82 (s, 3H, OCH_3_), 4.27–4.32 (m, 4H, OCH_2_CH_2_ CH_2_O), 6.25 (d, *J* = 9.6 Hz, 1H, Ar), 6.85–6.89 (m, 2H, Ar), 7.14 (d, *J* = 4.2 Hz, 2H, Ar), 7.37–7.42 (m, 2H, Ar), 7.64 (d, *J* = 9.3 Hz, 1H, Ar), 10.4 (s, 1H, CHO). ^**13**^ **C NMR** (75 MHz, CDCl_3_) δ: 29.8, 56.0, 65.0, 71.1, 101.5, 112.6, 112.7, 113.2, 118.0, 119.4, 124.3, 128.8, 130.0, 143.4, 151.4, 152.9, 155.9, 161.2, 162.1, 190.0. Anal. Calcd for C_20_H_18_O_6_: C, 67.79; H, 5.12. Found: C, 67.96; H, 5.19.

3-Ethoxy-4-(3-((2-oxo-2*H*-chromen-7-yl)­oxy)­propoxy)­benzaldehyde **(12)**. White solid, mp 153–154 °C, 80% yield. ^**1**^ **H NMR** (300 MHz, CDCl_3_) δ: 1.45 (t, *J* = 7.0 Hz, 3H, OCH_2_ CH_3_), 2.33–2.41 (m, 2H, OCH_2_ CH_2_CH_2_O), 4.13 (q, *J* = 7.0 Hz, 2H, OCH_2_CH_3_), 4.24–4.31 (m, 4H, OCH_2_CH_2_ CH_2_O), 6.23 (d, *J* = 9.3 Hz, 1H, Ar), 6.83–6.86 (m, 2H, Ar), 6.99 (d, *J* = 7.8 Hz, 1H, Ar), 7.34–7.43 (m, 4H, Ar), 7.62 (d, *J* = 9.6 Hz, 1H, Ar), 9.83 (s, 1H, CHO). ^**13**^ **C NMR** (75 MHz, CDCl_3_) δ: 14.7, 28.8, 64.5, 64.8, 65.3, 101.5, 110.8, 112.1, 112.6, 112.8, 113.2, 126.4, 128.8, 130.3, 143.3, 149.3, 154.0, 155.9, 161.1, 162.0, 190.9. Anal. Calcd for C_21_H_20_O_6_: C, 68.47; H, 5.47. Found: C, 68.21; H, 5.40.

3-Methoxy-4-(4-((2-oxo-2H-chromen-7-yl)­oxy)­butoxy)­benzaldehyde **(13)**. White solid, mp 129–130 °C, 89% yield. ^**1**^ **H NMR** (300 MHz, CDCl_3_) δ: 2.02–2.10 (m, 4H, OCH_2_ CH_2_ CH_2_CH_2_O), 3.91 (s, 3H, OCH_3_), 4.12 (t, *J* = 6.0 Hz, 2H, OCH_2_CH_2_CH_2_CH_2_O), 4.19 (t, *J* = 5.8 Hz, 2H, OCH_2_CH_2_CH_2_ CH_2_O), 6.25 (d, *J* = 9.3 Hz, 1H, Ar), 6.79–6.83 (m, 2H, Ar), 6.97 (d, *J* = 8.1 Hz, 1H, Ar), 7.34–7.45 (m, 3H, Ar), 7.63 (d, *J* = 9.6 Hz, 1H), 9.84 (s, 1H, CHO). ^**13**^ **C NMR** (75 MHz, CDCl_3_) δ: 25.6, 25.9, 29.7, 56.0, 68.1, 68.6, 101.3, 109.3, 111.4, 112.5, 112.9, 113.1, 126.7, 128.7, 130.1, 143.4, 149.8, 153.9, 155.9, 161.2, 162.2, 190.9. Anal. Calcd for C_21_H_20_O_6_: C, 68.47; H, 5.47. Found: C, 68.20; H, 5.39.

4-Methoxy-3-(4-((2-oxo-2*H*-chromen-7-yl)­oxy)­butoxy)­benzaldehyde **(14)**. White solid, mp 77–78 °C, 52% yield. ^**1**^ **H NMR** (300 MHz, CDCl_3_) δ: 2.04–2.07 (m, 4H, OCH_2_ CH_2_ CH_2_CH_2_O), 3.94 (s, 3H, OCH_3_), 4.11–4.18 (m, 4H, OCH_2_CH_2_CH_2_ CH_2_O), 6.24 (d, *J* = 9.3 Hz, 1H, Ar), 6.81–6.85 (m, 2H, Ar), 6.97 (d, *J* = 8.1 Hz, 1H, Ar), 7.34–7.47 (m, 3H, Ar), 7.63 (d, *J* = 9.6 Hz, 1H), 9.84 (s, 1H, CHO). ^**13**^ **C NMR** (75 MHz, CDCl_3_) δ: 25.6, 26.0, 56.2, 68.2, 68.5, 101.3, 110.3, 110.2, 110.6, 113.0, 126.9, 128.7, 143.4, 148.9, 190.8. Anal. Calcd for C_21_H_20_O_6_: C, 68.47; H, 5.47. Found: C, 68.28; H, 5.39.

3-Methoxy-2-(4-((2-oxo-2*H*-chromen-7-yl)­oxy)­butoxy)­benzaldehyde **(15)**. White solid, mp 83–85 °C, 45% yield. ^**1**^ **H NMR** (300 MHz, CDCl_3_) δ: 2.01–2.04 (m, 4H, OCH_2_ CH_2_ CH_2_CH_2_O), 3.89 (s, 3H, OCH_3_), 4.11 (t, *J* = 5.9 Hz, 2H, OCH_2_CH_2_CH_2_CH_2_O), 4.20 (t, *J* = 6.2 Hz, 2H, OCH_2_CH_2_CH_2_ CH_2_O), 6.25 (d, *J* = 9.3 Hz, 1H, Ar), 7.13–7.15 (m, 2H, Ar), 7.35–7.43 (m, 2H, Ar), 7.63 (d, *J* = 9.9 Hz, 1H), 10.45 (s, 1H, CHO). ^**13**^ **C NMR** (75 MHz, CDCl_3_) δ: 25.7, 26.7, 56.0, 68.1, 74.3, 101.4, 112.5, 112.9, 113.0, 118.1, 119.3, 124.1, 128.8, 130.0, 143.4, 151.7, 153.0, 155.9, 161.2, 162.2, 190.2. Anal. Calcd for C_21_H_20_O_6_: C, 68.47; H, 5.47. Found: C, 68.60; H, 5.53.

3-Ethoxy-4-(4-((2-oxo-2*H*-chromen-7-yl)­oxy)­butoxy)­benzaldehyde **(16)**. White solid, mp 122–123 °C, 64% yield. ^**1**^ **H NMR** (300 MHz, CDCl_3_) δ: 1.46 (t, *J* = 7.0 Hz, 3H, OCH_2_CH_3_), 2.06–2.13 (m, 4H, OCH_2_ CH_2_ CH_2_CH_2_O), 4.10–4.20 (m, 6H, OCH_2_CH_2_CH_2_ CH_2_O and OCH_2_CH_3_), 6.25 (d, *J* = 9.3 Hz, 1H, Ar), 6.81–6.83 (m, 2H, Ar), 6.97 (d, *J* = 8.1 Hz, 1H, Ar), 7.34–7.44 (m, 3H, Ar), 7.63 (d, *J* = 9.6 Hz, 1H, Ar), 9.84 (s, 1H, CHO). ^**13**^ **C NMR** (75 MHz, CDCl_3_) δ: 14.7, 25.6, 25.7, 64.5, 68.1, 68.6, 101.4, 110.9, 111.8, 112.5, 112.9, 113.1, 126.4, 128.7, 130.2, 143.3, 149.2, 154.2, 155.9, 161.2, 162.2, 190.9. Anal. Calcd for C_22_H_22_O_6_: C, 69.10; H, 5.80. Found: C, 69.01; H, 5.87.

3-Methoxy-4-((5-((2-oxo-2*H*-chromen-7-yl)­oxy)­pentyl)­oxy)­benzaldehyde **(17)**. White solid, mp 103–104 °C, 78% yield. ^**1**^ **H NMR** (300 MHz, CDCl_3_) δ: 1.66–1.72 (m, 2H, OCH_2_CH_2_ CH_2_CH_2_CH_2_O), 1.89–1.99 (m, 4H, OCH_2_ CH_2_CH_2_ CH_2_CH_2_O), 3.91 (s, 3H, OCH_3_), 4.05 (t, *J* = 6.1 Hz, 2H, OCH_2_CH_2_CH_2_CH_2_CH_2_O), 4.14 (t, *J* = 6.4 Hz, 2H, OCH_2_CH_2_CH_2_CH_2_ CH_2_O), 6.23 (d, *J* = 9.3 Hz, 1H, Ar), 6.78–6.83 (m, 2H, Ar), 6.97 (d, *J* = 8.4 Hz, 1H, Ar), 7.34 (d, *J* = 8.1 Hz, 1H, Ar), 7.40–7.45 (m, 2H, Ar), 7.63 (d, *J* = 9.6 Hz, 1H, Ar), 9.84 (s, 1H, CHO). ^**13**^ **C NMR** (75 MHz, CDCl_3_) δ: 22.6, 28.6, 28.7, 56.0, 68.3, 68.8, 101.3, 109.3, 111.4, 112.5, 112.9, 113.0, 126.7, 128.7, 130.0, 143.4, 149.8, 154.0, 155.9, 161.2, 162.3, 190.9. Anal. Calcd for C_22_H_22_O_6_: C, 69.10; H, 5.80. Found: C, 69.27; H, 5.89.

4-Methoxy-3-((5-((2-oxo-2*H*-chromen-7-yl)­oxy)­pentyl)­oxy)­benzaldehyde **(18)**. White solid, mp 138–139 °C, 79% yield. ^**1**^ **H NMR** (300 MHz, CDCl_3_) δ: 1.68–1.71 (m, 2H, OCH_2_CH_2_ CH_2_CH_2_CH_2_O), 1.88–1.98 (m, 4H, OCH_2_ CH_2_CH_2_ CH_2_CH_2_O), 3.94 (s, 3H, OCH_3_), 4.05 (t, *J* = 6.1 Hz, 2H, OCH_2_CH_2_CH_2_CH_2_CH_2_O), 4.11 (t, *J* = 6.4 Hz, 2H, OCH_2_CH_2_CH_2_CH_2_ CH_2_O), 6.23 (d, *J* = 9.3 Hz, 1H, Ar), 6.79–6.84 (m, 2H, Ar), 6.98 (d, *J* = 8.1 Hz, 1H, Ar), 7.35 (d, *J* = 8.7 Hz, 1H, Ar), 7.40 (d, *J* = 1.5 Hz, 1H, Ar), 7.44 (dd, *J* = 8.1, 1.8 Hz, 1H, Ar), 7.62 (d, *J* = 9.6 Hz, 1H, Ar), 9.83 (s, 1H, CHO). ^**13**^ **C NMR** (75 MHz, CDCl_3_) δ: 22.6, 28.7, 56.2, 68.3, 68.7, 101.4, 110.3, 110.6, 112.4, 112.9, 113.0, 126.8, 128.7, 130.1, 143.4, 149.0, 154.8, 155.9, 161.2, 162.3, 190.9. Anal. Calcd for C_22_H_22_O_6_: C, 69.10; H, 5.80. Found: C, 68.98; H, 5.73.

3-Methoxy-2-((5-((2-oxo-2*H*-chromen-7-yl)­oxy)­pentyl)­oxy)­benzaldehyde **(19)**. White solid, mp 75–76 °C, 58% yield. ^**1**^ **H NMR** (300 MHz, CDCl_3_) δ: 1.66–1.74 (m, 2H, OCH_2_CH_2_ CH_2_CH_2_CH_2_O), 1.84–1.95 (m, 4H, OCH_2_ CH_2_CH_2_ CH_2_CH_2_O), 3.89 (s, 3H, OCH_3_), 4.05 (t, *J* = 6.4 Hz, 2H, OCH_2_), 4.16 (t, *J* = 6.4 Hz, 2H, OCH_2_), 6.23 (d, *J* = 9.3 Hz, 1H, Ar), 6.80 (ddd, *J* = 8.0, 7.5, 2.4 Hz, 2H, Ar), 7.11–7.15 (m, 2H, Ar), 7.34–7.43 (m, 2H, Ar), 7.63 (d, *J* = 9.6 Hz, 1H, Ar), 10.45 (s, 1H, CHO). ^**13**^ **C NMR** (75 MHz, CDCl_3_) δ: 22.5, 28.8, 29.8, 56.0, 68.4, 74.6, 101.4, 112.5, 112.9, 113.0, 118.1, 119.2, 124.0, 128.7, 130.0, 143.4, 151.9, 153.0, 155.9, 161.2, 162.3, 190.3. Anal. Calcd for C_22_H_22_O_6_: C, 69.10; H, 5.80. Found: C, 69.24; H, 5.91.

3-Ethoxy-4-((5-((2-oxo-2*H*-chromen-7-yl)­oxy)­pentyl)­oxy)­benzaldehyde **(20)**. White solid, mp 88–89 °C, 52% yield. ^**1**^ **H NMR** (300 MHz, CDCl_3_) δ: 1.45 (t, *J* = 6.7 Hz, 3H, OCH_2_ CH_3_), 1.67–1.73 (m, 2H, OCH_2_CH_2_ CH_2_CH_2_CH_2_O), 1.90–1.98 (m, 4H, OCH_2_ CH_2_CH_2_ CH_2_CH_2_O), 4.05 (t, *J* = 6.4 Hz, 2H, OCH_2_CH_3_), 4.10–4.17 (m, 4H, OCH_2_CH_2_CH_2_CH_2_ CH_2_O), 6.24 (d, *J* = 9.3 Hz, 2H, Ar), 6.78–6.83 (m, 2H, Ar), 6.96 (d, *J* = 8.4 Hz, 1H, Ar), 7.34–7.43 (m, 3H, Ar), 7.63 (d, *J* = 9.6 Hz, 1H, Ar), 9.83 (s, 1H, CHO). ^**13**^ **C NMR** (75 MHz, CDCl_3_) δ: 14.7, 22.7, 28.6, 28.7, 64.5, 68.3, 68.9, 101.3, 110.9, 111.8, 112.5, 112.5, 112.9, 113.0, 126.5, 128.7, 130.0, 126.5, 128.7, 130.0, 143.4, 149.2, 154.3, 155.9, 161.2, 162.3, 190.9. Anal. Calcd for C_23_H_24_O_6_: C, 69.68; H, 6.10. Found: C, 69.77; H, 6.18.

3-Methoxy-4-((6-((2-oxo-2*H*-chromen-7-yl)­oxy)­hexyl)­oxy)­benzaldehyde **(21)**. White solid, mp 92–93 °C, 71% yield. ^**1**^ **H NMR** (300 MHz, CDCl_3_) δ: 1.57–1.59 (m, 4H, OCH_2_CH_2_ CH_2_ CH_2_CH_2_CH_2_O), 1.83–1.93 (m, 4H, OCH_2_ CH_2_CH_2_CH_2_ CH_2_CH_2_O), 3.91 (s, 3H, OCH_3_), 4.02 (t, *J* = 6.1 Hz, 2H, OCH_2_CH_2_CH_2_CH_2_CH_2_CH_2_O), 4.12 (t, *J* = 6.7 Hz, 2H, OCH_2_CH_2_CH_2_CH_2_CH_2_ CH_2_O), 6.24 (d, *J* = 9.3 Hz, 1H, Ar), 6.78–6.83 (m, 2H, Ar), 6.96 (d, *J* = 8.4 Hz, 1H, Ar), 7.33–7.44 (m, 3H, Ar), 7.63 (d, *J* = 9.6 Hz, 1H, Ar), 9.84 (s, 1H, CHO). ^**13**^ **C NMR** (75 MHz, CDCl_3_) δ: 25.7, 25.7, 28.9, 56.0, 68.4, 68.9, 101.3, 109.3, 111.4, 112.4, 112.9, 126.8, 128.7, 129.9, 143.4, 149.8, 154.1, 155.9, 161.3, 162.3, 190.9. Anal. Calcd for C_23_H_24_O_6_: C, 69.68; H, 6.10. Found: C, 69.81; H, 6.01.

4-Methoxy-3-((6-((2-oxo-2*H*-chromen-7-yl)­oxy)­hexyl)­oxy)­benzaldehyde **(22)**. White solid, mp 99–101 °C, 89% yield. ^**1**^ **H NMR** (300 MHz, CDCl_3_) δ: 1.56–1.59 (m, 4H, OCH_2_CH_2_ CH_2_CH_2_CH_2_O), 1.83–1.93 (m, 4H, OCH_2_ CH_2_CH_2_CH_2_ CH_2_CH_2_O), 3.94 (s, 3H, OCH_3_), 4.02 (t, *J* = 6.4 Hz, 2H, OCH_2_CH_2_CH_2_CH_2_CH_2_CH_2_O), 4.09 (t, *J* = 6.7 Hz, 2H, OCH_2_CH_2_CH_2_CH_2_CH_2_ CH_2_O), 6.24 (d, *J* = 9.3 Hz, 1H, Ar), 6.79–6.84 (m, 2H, Ar), 6.97 (d, *J* = 8.1 Hz, 1H, Ar), 7.36–7.45 (m, 3H, Ar), 7.63 (d, *J* = 9.3 Hz, 1H, Ar), 9.83 (s, 1H, CHO). ^**13**^ **C NMR** (75 MHz, CDCl_3_) δ: 25.7, 25.7, 28.9, 56.2, 68.4, 68.8, 101.3, 110.0, 110.2, 110.6, 112.4, 113.0, 126.7, 128.7, 130.1, 143.4, 149.1, 154.8, 155.9, 161.2, 162.4, 190.9. Anal. Calcd for C_23_H_24_O_6_: C, 69.68; H, 6.10. Found: C, 69.50; H, 6.02.

3-Methoxy-2-((6-((2-oxo-2*H*-chromen-7-yl)­oxy)­hexyl)­oxy)­benzaldehyde **(23)**. White solid, mp 102–104 °C, 70% yield. ^**1**^ **H NMR** (300 MHz, CDCl_3_) δ: 1.54–1.59 (m, 4H, OCH_2_CH_2_ CH_2_ CH_2_CH_2_CH_2_O), 1.82–1.88 (m, 4H, OCH_2_ CH_2_CH_2_CH_2_ CH_2_CH_2_O), 3.89 (s, 3H, OCH_3_), 4.07 (t, *J* = 6.5 Hz, 2H, OCH_2_CH_2_CH_2_CH_2_CH_2_CH_2_O), 4.13 (t, *J* = 6.5 Hz, 2H, OCH_2_CH_2_CH_2_CH_2_CH_2_ CH_2_O), 6.24 (d, *J* = 9.3 Hz, 1H, Ar), 6.79–6.85 (m, 2H, Ar), 7.11–7.14 (m, 2H, Ar), 7.34–7.43 (m, 2H, Ar), 7.63 (d, *J* = 9.6 Hz, 1H, Ar), 10.45 (s, 1H, CHO). ^**13**^ **C NMR** (75 MHz, CDCl_3_) δ: 25.7, 25.8, 28.9, 30.0, 56.0, 68.4, 74.8, 101.3, 112.4, 112.9, 118.1, 119.1, 124.0, 128.7, 130.0, 143.5, 152.0, 153.1, 155.9, 161.3, 162.3, 190.3. Anal. Calcd for C_23_H_24_O_6_: C, 69.68; H, 6.10. Found: C, 69.59; H, 6.03.

3-Ethoxy-4-((6-((2-oxo-2*H*-chromen-7-yl)­oxy)­hexyl)­oxy)­benzaldehyde **(24)**. White solid, mp 107–109 °C, 89% yield. ^**1**^ **H NMR** (300 MHz, CDCl_3_) δ: 1.46 (t, *J* = 7.1 Hz, 3H, OCH_2_CH_3_), 1.56–1.60 (m, 4H, OCH_2_CH_2_ CH_2_ CH_2_CH_2_CH_2_O), 1.84–1.92 (m, 4H, OCH_2_ CH_2_CH_2_CH_2_ CH_2_CH_2_O), 4.02 (t, *J* = 6.5 Hz, 2H, OCH_2_CH_3_), 4.09–4.14 (m, 4H, OCH_2_CH_2_CH_2_CH_2_CH_2_ CH_2_O), 6.24 (d, *J* = 9.3 Hz, 1H, Ar), 6.79–6.83 (m, 2H, Ar), 6.96 (d, *J* = 8.4 Hz, 1H, Ar), 7.34–7.43 (m, 3H, Ar), 7.64 (d, *J* = 9.6 Hz, 1H, Ar), 9.83 (s, 1H, CHO). ^**13**^ **C NMR** (75 MHz, CDCl_3_) δ: 14.7, 25.7, 28.8, 28.9, 64.5, 68.4, 68.9, 101.3, 110.8, 111.7, 112.4, 113.0, 126.6, 128.7, 129.9, 143.3, 149.1, 154.4, 191.0. Anal. Calcd for C_24_H_26_O_6_: C, 70.23; H, 6.38. Found: C, 70.29; H, 6.41.

##### 4.1.2.2 General Procedure for the Synthesis of Compounds **25–33** (Series B)

Azido intermediates (**38–41**). The proper bromo derivative **34–37** (1.0 equiv) was dissolved in DMF (1.0 M), and NaN_3_ (1.2 equiv) was added. The reaction mixture was allowed to stir for 2 h at room temperature. After completion, the reaction mixture was quenched with H_2_O and extracted with EtOAc three times. The combined organic layers were dried over anhydrous Na_2_SO_4_, filtered, and evaporated under reduced pressure. Compounds were obtained with no further purification and were directly used in the next step.

Propargylated umbelliferon (**42**). To **UMB** (**1**) (1.0 equiv) in ACN (0.4 M), propargyl bromide (80 wt % in toluene) (1.5 equiv) and K_2_CO_3_ (4.0 equiv) were added, and the reaction mixture was allowed to reflux for 12 h. After completion, the reaction mixture was poured into cold H_2_O and extracted with EtOAc three times. The combined organic layers were dried over anhydrous Na_2_SO_4_, filtered, and evaporated under reduced pressure. The crude reaction mixture was then purified through flash column chromatography on silica gel by eluting with an 8:2 *n*-hexane/EtOAc mixture. Characterization data are in accordance with previous literature.^44^

Triazole hybrids **25–33**. The suitable azido derivative **38–41** (1.2 equiv), propargylated **UMB 42** (1.0 equiv), CuSO_4_ 5H_2_O (0.01 equiv), and sodium ascorbate (0.1 equiv) were dissolved in a mixture of 1:1 H_2_O and *t*BuOH (0.25 M) and stirred at room temperature overnight. After completion, the reaction mixture was poured into cold H_2_O and extracted with EtOAc three times. The combined organic layers were dried over anhydrous Na_2_SO_4_, filtered, and evaporated under reduced pressure. The crude reaction mixture was then purified through flash column chromatography on silica gel with suitable *n*-hexane/EtOAc mixtures and, in some cases, recrystallized from ethanol to afford final compounds **25**–**33**.

3-Methoxy-4-(4-(4-(((2-oxo-2*H*-chromen-7-yl)­oxy)­methyl)-1*H*-1,2,3-triazol-1-yl)­butoxy)­benzaldehyde **(25)**. White solid, mp 53–55 °C, 81% yield. ^**1**^ **H NMR** (300 MHz, DMSO) δ: 1.68–1.75 (m, 2H, OCH_2_ CH_2_CH_2_CH_2_N), 1.93–2.02 (m, 2H, OCH_2_CH_2_ CH_2_CH_2_N), 3.81 (s, 3H, OCH_3_), 4.09 (t, *J* = 6.2 Hz, 2H, OCH_2_CH_2_CH_2_CH_2_N), 4.55 (t, *J* = 6.9 Hz, 2H, OCH_2_CH_2_CH_2_ CH_2_N), 5.25 (s, 2H, CH_2_–Triazole), 6.27 (d, *J* = 9.3 Hz, 1H, Ar), 7.00 (dd, *J* = 9.0, 2.4 Hz, 1H, Ar), 7.12–7.15 (m, 2H, Ar), 7.37 (s, 1H, Ar), 7.51 (dd, *J* = 8.1, 2.4 Hz, 1H, Ar), 7.62 (d, *J* = 8.7 Hz, 1H, Ar), 7.97 (d, *J* = 9.3 Hz, 1H, Ar), 8.29 (s, 1H, Triazole), 9.81 (s, 1H, CHO). ^**13**^ **C NMR** (75 MHz, DMSO) δ: 25.9, 27.0, 49.6, 62.2, 68.4, 102.1, 112.6, 113.0, 113.4, 125.3, 125.3, 126.4, 130.0, 142.4, 144.7, 153.9, 161.6, 191.8. Anal. Calcd for C_24_H_23_N_3_O_6_: C, 64.10; H, 5.16; N, 9.35. Found: C, 64.27; H, 5.22; N, 9.43.

4-Methoxy-3-(4-(4-(((2-oxo-2*H*-chromen-7-yl)­oxy)­methyl)-1*H*-1,2,3-triazol-1-yl)­butoxy)­benzaldehyde **(26)**. White solid, mp 133–135 °C, 71% yield. ^**1**^ **H NMR** (300 MHz, CDCl_3_) δ: 1.87 (quint, *J* = 6.2 Hz, 2H, OCH_2_ CH_2_CH_2_CH_2_N), 2.17 (quint, *J* = 7.1 Hz, 2H, OCH_2_CH_2_ CH_2_CH_2_N), 3.94 (s, 3H, OCH_3_), 4.11 (t, *J* = 6.1 Hz, 2H, OCH_2_CH_2_CH_2_CH_2_N), 4.53 (t, *J* = 7.0 Hz, 2H, OCH_2_CH_2_CH_2_ CH_2_N), 5.24 (s, 2H, CH_2_–Triazole), 6.24 (d, *J* = 9.3 Hz, 1H, Ar), 6.92–6.99 (m, 3H, Ar), 7.36–7.40 (m, 2H, Ar), 7.46 (dd, *J* = 8.2, 1.8 Hz, 1H, Ar), 7.63 (d, *J* = 10.2 Hz, 1H, Ar), 7.82 (s, 1H, Triazole), 9.82 (s, 1H, CHO). ^**13**^ **C NMR** (75 MHz, CDCl_3_) δ: 25.7, 27.6, 50.0, 56.1, 62.4, 68.4, 102.2, 110.3, 110.7, 112.7, 113.0, 113.5, 123.3, 127.0, 128.9, 130.1, 142.8, 143.3, 148.8, 154.7, 155.7, 161.0, 161.4, 190.8. Anal. Calcd for C_24_H_23_N_3_O_6_: C, 64.10; H, 5.16; N, 9.35. Found: C, 64.00; H, 5.11; N, 9.24.

4-Methoxy-3-((5-(4-(((2-oxo-2*H*-chromen-7-yl)­oxy)­methyl)-1*H*-1,2,3-triazol-1-yl)­pentyl)­oxy)­benzaldehyde **(27)**. Yellow solid, mp 107–108 °C, 70% yield. ^**1**^ **H NMR** (300 MHz, DMSO) δ: 1.32–1.42 (m, 2H, OCH_2_CH_2_ CH_2_CH_2_CH_2_N), 1.75 (quint, *J* = 6.2 Hz, 2H, OCH_2_ CH_2_CH_2_CH_2_CH_2_N), 1.86 (quint, *J* = 7.0 Hz, 2H, OCH_2_CH_2_CH_2_CH_2_ CH_2_N), 3.83 (s, 3H, OCH_3_), 3.98 (t, *J* = 6.4 Hz, 2H, OCH_2_CH_2_CH_2_CH_2_CH_2_N), 4.39 (t, *J* = 7.0 Hz, 2H, OCH_2_CH_2_CH_2_CH_2_ CH_2_N), 5.24 (s, 2H, CH_2_–Triazole), 6.28 (d, *J* = 9.3 Hz, 2H, Ar), 7.00 (dd, *J* = 8.8, 2.4 Hz, 1H, Ar), 7.13–7.16 (m, 2H, Ar), 7.35 (d, *J* = 1.8 Hz, 1H, Ar), 7.53 (dd, *J* = 8.1, 1.8 Hz, 1H, Ar), 7.61 (d, *J* = 8.7 Hz, 1H, Ar), 7.97 (d, *J* = 9.3 Hz, 1H, Ar), 8.29 (s, 1H, Triazole), 9.80 (s, 1H, CHO). ^**13**^ **C NMR** (75 MHz, DMSO) δ: 28.7, 56.2, 64.8, 65.2, 101.3, 110.0, 110.4, 110.7, 112.5, 113.0, 113.2, 143.4, 148.8, 154.8, 155.8, 161.2, 162.0, 190.8. Anal. Calcd for C_25_H_25_N_3_O_6_: C, 64.79; H, 5.44; N, 9.07. Found: C, 64.61; H, 5.50; N, 9.15.

3-Methoxy-2-((5-(4-(((2-oxo-2*H*-chromen-7-yl)­oxy)­methyl)-1*H*-1,2,3-triazol-1-yl)­pentyl)­oxy)­benzaldehyde **(28)**. Colorless oil, 82% yield. ^**1**^ **H NMR** (300 MHz, DMSO) δ: 1.33–1.43 (m, 2H, OCH_2_CH_2_ CH_2_CH_2_CH_2_N), 1.73 (quint, *J* = 6.1 Hz, 2H, OCH_2_ CH_2_CH_2_CH_2_CH_2_N), 1.88 (quint, *J* = 7.1 Hz, 2H, OCH_2_CH_2_CH_2_ CH_2_CH_2_N), 3.35 (s, 3H, OCH_3_), 4.03 (t, *J* = 6.4 Hz, 2H, OCH_2_CH_2_CH_2_CH_2_CH_2_N), 4.39 (t, *J* = 7.0 Hz, 2H, OCH_2_CH_2_CH_2_CH_2_ CH_2_N), 5.24 (s, 2H, CH_2_–Triazole), 6.26 (d, *J* = 9.3 Hz, 1H, Ar), 6.98 (dd, *J* = 8.7, 2.4 Hz, 1H, Ar), 7.13–7.17 (m, 2H, Ar), 7.23 (dd, *J* = 7.8, 1.8 Hz, 1H, Ar), 7.34 (dd, *J* = 7.5, 1.8 Hz, 1H, Ar), 7.60 (d, *J* = 8.1 Hz, 1H, Ar), 7.95 (d, *J* = 9.6 Hz, 1H, Ar), 8.29 (s, 1H, Triazole), 10.27 (s, 1H, CHO). ^**13**^ **C NMR** (75 MHz, DMSO) δ: 22.8, 29.2, 29.8, 49.8, 56.5, 62.2, 74.4, 102.0, 113.0, 113.1, 113.4, 118.7, 119.3, 124.7, 125.2, 129.8, 129.9, 142.3, 144.7, 151.6, 153.3, 155.7, 160.7, 161.6, 190.4. Anal. Calcd for C_25_H_25_N_3_O_6_: C, 64.79; H, 5.44; N, 9.07. Found: C, 64.96; H, 5.49; N, 9.00.

3-Ethoxy-4-((5-(4-(((2-oxo-2*H*-chromen-7-yl)­oxy)­methyl)-1*H*-1,2,3-triazol-1-yl)­pentyl)­oxy)­benzaldehyde **(29)**. Pale-yellow solid, mp 67–69 °C, 67% yield. ^**1**^ **H NMR** (300 MHz, DMSO) δ: 1.29 (t, *J* = 6.7 Hz, 3H, OCH_2_ CH_3_), 1.33–1.40 (m, 2H, OCH_2_CH_2_ CH_2_CH_2_CH_2_N), 1.76 (quint, *J* = 6.2 Hz, 2H, OCH_2_ CH_2_CH_2_CH_2_CH_2_N), 1.87 (quint, *J* = 7.1 Hz, 2H, OCH_2_CH_2_CH_2_ CH_2_CH_2_N), 4.01–4.08 (m, 4H, OCH_2_CH_2_CH_2_CH_2_CH_2_N and OCH_2_CH_3_), 4.39 (t, *J* = 7.0 Hz, 2H, OCH_2_CH_2_CH_2_CH_2_ CH_2_N), 5.24 (s, 2H, CH_2_–Triazole), 6.29 (d, *J* = 9.3 Hz, 1H, Ar), 6.98 (dd, *J* = 8.8, 2.4 Hz, 1H, Ar), 7.12–7.15 (m, 2H, Ar), 7.34 (d, *J* = 1.8 Hz, 1H, Ar), 7.50 (dd, *J* = 8.4, 1.8 Hz, 1H, Ar), 7.62 (d, *J* = 9.0 Hz, 1H, Ar), 7.98 (d, *J* = 9.3 Hz, 1H, Ar), 8.28 (s, 1H, Triazole), 9.80 (s, 1H, CHO). ^**13**^ **C NMR** (75 MHz, DMSO) δ: 14.7, 23.2, 28.2, 29.9, 50.3, 62.4, 64.5, 68.7, 102.1, 110.9, 111.8, 112.7, 113.0, 113.5, 122.8, 125.5, 128.9, 130.2, 143.0, 143.2, 149.2, 154.2, 161.0, 190.9. Anal. Calcd for C_26_H_27_N_3_O_6_: C, 65.40; H, 5.70; N, 8.80. Found: C, 65.20; H, 5.59; N, 8.93.

3-Methoxy-4-((6-(4-(((2-oxo-2*H*-chromen-7-yl)­oxy)­methyl)-1*H*-1,2,3-triazol-1-yl)­hexyl)­oxy)­benzaldehyde **(30)**. Colorless oil, 81% yield. ^**1**^ **H NMR** (300 MHz, DMSO) δ: 1.36–1.44 (m, 2H, OCH_2_CH_2_ CH_2_CH_2_CH_2_CH_2_N), 1.47–1.57 (m, 2H, OCH_2_CH_2_CH_2_ CH_2_CH_2_CH_2_N), 1.79–1.90 (m, 2H, OCH_2_ CH_2_CH_2_CH_2_CH_2_CH_2_N), 1.92–2.01 (m, 2H, OCH_2_CH_2_CH_2_CH_2_ CH_2_CH_2_N), 3.88 (s, 3H, OCH_3_), 4.05 (t, *J* = 6.5 Hz, 2H, NCH_2_), 4.37 (t, *J* = 7.1 Hz, 2H, OCH_2_), 5.21 (s, 2H, CH_2_–Triazole), 6.22 (d, *J* = 9.6 Hz, 1H, Ar), 6.88–6.93 (m, 3H, Ar), 7.34–7.41 (m, 3H, Ar), 7.60 (d, *J* = 9.9 Hz, 1H, Ar), 7.64 (s, 1H, Triazole), 9.80 (s, 1H, CHO). ^**13**^ **C NMR** (75 MHz, DMSO) δ: 25.3, 26.1, 28.6, 30.1, 50.3, 56.0, 62.3, 68.7, 102.1, 109.3, 111.4, 112.7, 112.9, 113.4, 122.8, 126.7, 128.9, 130.0, 142.9, 143.3, 149.8, 154.0, 155.7, 161.0, 190.8. Anal. Calcd for C_26_H_27_N_3_O_6_: C, 65.40; H, 5.70; N, 8.80. Found: C, 65.24; H, 5.62; N, 8.96.

4-Methoxy-3-((6-(4-(((2-oxo-2*H*-chromen-7-yl)­oxy)­methyl)-1*H*-1,2,3-triazol-1-yl)­hexyl)­oxy)­benzaldehyde **(31)**. White solid, mp 63–65 °C, 67% yield. ^**1**^ **H NMR** (300 MHz, DMSO) δ: 1.21–1.32 (m, 2H, CH_2_), 1.36–1.46 (m, 2H, CH_2_), 1.64–1.73 (m, 2H, CH_2_), 1.77–1.87 (m, 2H, CH_2_), 3.84 (s, 3H, OCH_3_), 3.97 (t, *J* = 6.4 Hz, 2H, NCH_2_), 4.36 (t, *J* = 7.0 Hz, 2H, OCH_2_), 5.23 (s, 2H, CH_2_–Triazole), 6.27 (d, *J* = 9.9 Hz, 1H, Ar), 6.98 (dd, *J* = 8.7, 2.4 Hz, 1H, Ar), 7.12–7.15 (m, 2H, Ar), 7.34 (d, *J* = 1.8 Hz, 1H, Ar), 7.53 (dd, *J* = 8.4, 1.8 Hz, 1H, Ar), 7.61 (d, *J* = 8.1 Hz, 1H, Ar), 7.96 (d, *J* = 9.3 Hz, 1H, Ar), 8.27 (s, 1H, Triazole), 9.80 (s, 1H, CHO). ^**13**^ **C NMR** (75 MHz, DMSO) δ: 25.3, 26.0, 28.8, 30.0, 49.8, 56.4, 62.2, 68.5, 102.0, 111.0, 111.9, 113.0, 113.1, 113.4, 125.2, 126.4, 130.0, 142.3, 144.7, 148.9, 155.7, 160.7, 161.6, 191.9. Anal. Calcd for C_26_H_27_N_3_O_6_: C, 65.40; H, 5.70; N, 8.80. Found: C, 65.65; H, 5.78; N, 8.63.

3-Methoxy-2-((6-(4-(((2-oxo-2*H*-chromen-7-yl)­oxy)­methyl)-1*H*-1,2,3-triazol-1-yl)­hexyl)­oxy)­benzaldehyde **(32)**. White solid, mp 99–101 °C, 62% yield. ^**1**^ **H NMR** (300 MHz, DMSO) δ: 1.21–1.31 (m, 2H, OCH_2_CH_2_ CH_2_CH_2_CH_2_CH_2_N), 1.37–1.47 (m, 2H, OCH_2_CH_2_CH_2_ CH_2_CH_2_CH_2_N), 1.67 (quint, *J* = 6.2 Hz, 2H, OCH_2_ CH_2_CH_2_CH_2_CH_2_CH_2_N), 1.83 (quint, *J* = 7.1 Hz, 2H, OCH_2_CH_2_CH_2_CH_2_ CH_2_CH_2_N), 3.34 (s, 3H, OCH_3_), 4.03 (t, *J* = 6.4 Hz, 2H, OCH_2_CH_2_CH_2_CH_2_CH_2_CH_2_N), 4.36 (t, *J* = 7.0 Hz, 2H, OCH_2_CH_2_CH_2_CH_2_CH_2_ CH_2_N), 5.23 (s, 2H, CH_2_–Triazole), 6.27 (d, *J* = 9.3 Hz, 1H, Ar), 7.00 (dd, *J* = 8.8, 2.4 Hz, 1H, Ar), 7.13–7.19 (m, 2H, Ar), 7.25 (dd, *J* = 7.8, 1.8 Hz, 1H, Ar), 7.34 (dd, *J* = 7.5, 1.8 Hz, 1H, Ar), 7.62 (d, *J* = 8.7 Hz, 1H, Ar), 7.97 (d, *J* = 9.3 Hz, 1H, Ar), 8.28 (s, 1H, Triazole), 10.28 (s, 1H, CHO). ^**13**^ **C NMR** (75 MHz, DMSO) δ: 25.2, 26.0, 29.7, 30.1, 49.8, 56.5, 62.2, 74.5, 102.0, 113.0, 113.1, 113.4, 118.7, 119.3, 124.6, 125.2, 129.8, 130.0, 142.3, 144.8, 151.6, 153.3, 155.7, 161.6, 190.5. Anal. Calcd for C_26_H_27_N_3_O_6_: C, 65.40; H, 5.70; N, 8.80. Found: C, 65.22; H, 5.59; N, 8.91.

3-Ethoxy-4-((6-(4-(((2-oxo-2*H*-chromen-7-yl)­oxy)­methyl)-1*H*-1,2,3-triazol-1-yl)­hexyl)­oxy)­benzaldehyde **(33)**. Colorless oil, 74% yield. ^**1**^ **H NMR** (300 MHz, DMSO) δ: 1.42–1.47 (m, 5H, OCH_2_CH_3_ and CH_2_), 1.50–1.60 (m, 2H, CH_2_), 1.81–1.90 (m, 2H, CH_2_), 1.92–2.02 (m, 2H, CH_2_), 4.07 (t, *J* = 6.4 Hz, 2H, NCH_2_), 4.14 (q, 2H, OCH_2_CH_3_), 4.39 (t, *J* = 7.0 Hz, 2H, OCH_2_), 5.24 (s, 2H, CH_2_–Triazole), 6.25 (d, *J* = 9.6 Hz, 1H, Ar), 6.91–6.95 (m, 3H, Ar), 7.36–7.42 (m, 3H, Ar), 7.61 (s, 1H, Triazole), 7.64 (d, *J* = 1.8, 1H, Ar) 9.81 (s, 1H, CHO). ^**13**^ **C NMR** (75 MHz, DMSO) δ: 14.7, 25.3, 26.1, 28.7, 30.1, 50.3, 62.4, 64.5, 68.7, 102.1, 111.0, 111.8, 112.7, 113.5, 122.8, 126.5, 128.9, 130.0, 143.2, 149.2, 161.0, 161.3, 190.9. Anal. Calcd for C_27_H_29_N_3_O_6_: C, 65.97; H, 5.95; N, 8.55. Found: C, 66.13; H, 6.03; N, 8.69.

### 4.2 CA Inhibition Assay

An Applied Photophysics stopped-flow instrument measured the CA-catalyzed CO_2_ hydration activity.^42^ The indicator used was phenol red (0.2 mM), with measurements taken at an absorbance maximum of 557 nm. HEPES (10 mM, pH 7.5) supplemented with 0.1 M Na_2_SO_4_ served as the reaction buffer, and the CA-catalyzed CO_2_ hydration reaction was monitored for 10–100 s. The CO_2_ concentrations varied from 1.7 to 17 mM. The uncatalyzed rates were also measured and subtracted. Stock solutions of inhibitors (10 mM) were provided in distilled–deionized water containing 10% DMSO, and dilutions of up to 0.001 μM were prepared with the reaction buffer. Inhibitor and enzyme solutions were preincubated together for 6 h at room temperature prior to the assay in order to allow for the formation of the E–I complex. The inhibition constants were obtained by nonlinear least-squares methods using PRISM 3 and the Cheng–Prusoff equation,^48^ representing the mean from at least three different determinations. The enzyme concentrations were in the range of 4–15 nM. All CA isoforms were recombinant ones obtained *in-house*, as reported earlier.^13,34^

### 4.3 In Silico Studies

#### 4.3.1 X-ray Structures

The crystal structures of hCA I (PDB code 1AZM), hCA II (PDB code 4E3H), hCA IX (PDB code 5FL4), and hCA XII (PDB code 1JCZ) were downloaded from the Protein Data Bank.^49^ Automated docking was carried out for the hydrolyzed form of compound **7**, whose structure was built using MolBook Pro software.^50^ All docking calculations were performed using the GOLD 5.1 program,^51,52^ using the PLP scoring function. The “allow early termination” command was deactivated, whereas the possibility for the ligand to flip ring corners was activated. The remaining GOLD default parameters were used for the calculations, in which the ligand was subjected to 100 genetic algorithm runs, and the corresponding docking results were clustered using a RMSD cutoff of 2.0 Å. For each docking calculation, the top-scoring pose of the best cluster of solutions was taken into account.

#### 4.3.2 Molecular Dynamics Simulations

MD simulations were carried out with AMBER, version 22. Each complex was subjected to an MD procedure based on already successfully applied protocols, using the ff14SB force field at 300 K.^53,54^ General Amber force field (GAFF) parameters were used for the ligand, whose partial charges were calculated with the AM1-BCC method, as implemented in the Antechamber suite of AMBER 22. Prior to MD simulations, each complex was located in a rectangular parallelepiped water box and solvated with a 15 Å water cap using the TIP3P explicit solvent model for water. Sodium ions were then added as counterions for the neutralization of the solvated systems. Each system was subjected to two stages of energy minimization, each composed of 5000 steps of steepest descent, followed by conjugate gradient until a convergence of 0.05 kcal/(mol·Å^2^) was reached. In the first stage, the whole protein was blocked with a position restraint of 500 kcal/(mol·Å^2^) to uniquely minimize the position of the water molecules, while in the second stage, the entire system was energy-minimized by applying a harmonic potential of 10 kcal/(mol·Å^2^) only to the protein α carbons. The minimized complexes were then used as the starting point for a total of 100 ns of the MD simulation. A 0.5 ns constant-volume simulation in which the temperature of the system was raised from 0 to 300 K was initially performed. In the second step, the system was equilibrated through a 3 ns constant-pressure simulation, maintaining the temperature at a constant value of 300 K with the use of a Langevin thermostat. An additional 96.5 ns of constant-pressure MD was then performed, for a total of 100 ns of simulation. In all three MD steps, a harmonic potential of 10 kcal/(mol·Å^2^) was applied to the protein α carbons. All simulations were performed using particle mesh Ewald (PME) electrostatics with a cutoff of 10 Å for nonbonded interactions and periodic boundary conditions. A simulation step of 2.0 fs was employed as all bonds involving hydrogen atoms were kept rigid using the SHAKE algorithm. Further 200 ns MD simulations were performed for **7**-CA XII and **7**-CA IX complexes, using the same parameters employed for the third constant-pressure MD step. This additional MD stage was performed using the hydrogen mass repartition (HMR) scheme^55^ and a time step of 4.0 fs, since this technique proved to be useful to reduce the simulation time while maintaining an unbiased MD protocol.^56,57^ The analysis of the MD trajectories was performed with the Cpptraj software included in the AMBER suite.

### 4.4 Cell-Based Assays

#### 4.4.1 Cell Cultures

Human bronchial epithelial (BEAS-2B; CRL-3588) and human lung carcinoma (A549; CCL-185) cell lines were purchased from ATCC. BEAS-2B and A549 cells were cultured in complete RPMI 1640 (Merck, Darmstadt, Germany) at 37 °C and 5% CO_2_. The medium was supplemented with 10% heat-inactivated fetal bovine serum (FBS), 1% penicillin–streptomycin, and 1% sodium pyruvate.

#### 4.4.2 Cell Treatment

Cells were seeded according to the different experimental techniques and left to adhere for 24 h. Next, the medium was removed, and cells were treated with selected compounds **7**–**11**, **26**, and **33**, which were dissolved in dimethyl sulfoxide (DMSO) to achieve a range of concentrations from 0 μM (untreated control = CTRL) to 100 μM. The percentage of DMSO was maintained below 0.3%. The viability of the cells was assessed after 24, 48, and 72 h.

#### 4.4.3 Cell Viability (MTT Assay)

BEAS-2B and A549 cells were seeded in 96-well culture-treated plates (Falcon, Corning Incorporated, Brooklyn, NY, USA) at 0.5 × 10^4^ cells/well. Untreated cells were set as the experimental control (100% of cell metabolic activity). Exposure times varied from 24 to 72 h. At the established time points (24, 48, and 72 h), exposure media were replaced with a fresh medium containing 3-(4,5-dimethylthiazol-2-yl)-2,5-diphenyltetrazolium bromide (MTT) 0.5 mg/mL (Merck, Darmstadt, Germany) and processed as elsewhere reported.^58^ The optical density in each well was measured by using a spectrophotometer (Thermo Fisher Scientific, Waltham, MA, USA) at a wavelength of 540 nm. Each experiment was performed twice in triplicate per experimental condition (*n* = 6).

#### 4.4.4 Cytotoxicity Assay (LDH Test)

To assess the cytotoxicity in A549 cells after treatments, the amount of lactate dehydrogenase released into the culture medium was measured using the CytoTox 96 nonradioactive cytotoxicity assay (Promega, Madison, WI, USA) according to the manufacturer’s instructions.^59^ Cell supernatants were harvested from the same cultures processed for the MTT assay after 24 h of treatment, and the measured optical density values were normalized to those obtained from the MTT assay.

#### 4.4.5 Cell Cycle Analysis

A549 cells were seeded in 12-well plates at 0.5 × 10^5^ cells/well and treated for 6 and 24 h, as previously described. At each time point, cells were detached using StemPro Accutase, collected by centrifugation, and then fixed overnight at 4 °C in 70% v/v cold ethanol. After fixation, cells were processed as described elsewhere.^60^ The PI fluorescence was detected by a flow cytometer equipped with a 488 nm laser (CytoFlex flow cytometer, Beckman Coulter, CA, USA) in the FL-3 channel. At least 10,000 events/sample were collected and analyzed with the CytExpert Software, version 2.3 (Beckman Coulter, CA, USA), and the percentages of cells in the G1, S, or G2 phase of the cell cycle were calculated using the ModFit LT Software, version 5.0 (Verity Software House, Topsham, ME, USA).

#### 4.4.6 Imaging and Analysis

Images were acquired using the high-content imaging microscope Operetta CLS (Revvity, Waltham, Massachusetts, U.S.), using a 20× water objective. Thirty-five fields were imaged for each replicate, and mean values were considered. Harmony Software was used for image processing and analysis. Briefly, cells were segmented to obtain the nuclei population (setting the channel on the DAPI and using method B) and the related cytoplasm localization (setting the channel on the Alexa 488 and using Method A). Then, the cell mean fluorescence intensity was calculated for each condition. Mean values for each well were then used for statistical analysis.

#### 4.4.7 Establishment of Pro-inflammatory Conditions

To establish an inflamed environment, differentiated macrophages were treated with LPS 0.5 μg/mL (lipopolysaccharide from *E. coli* purchased from Merck, Darmstadt, Germany, stock solution 1 mg/mL in water) for 24 h. BEAS-2B cells were treated with the medium collected from LPS-stimulated macrophage cultures and afterward exposed to treatments. Cells were fixed with 4% glutaraldehyde and stained with crystal violet. Images were acquired and analyzed by the Leica Application Suite LAS EZ version 3.4 (Leica, Wetzlar, Germany).

#### 4.4.8 Statistical Analysis

Statistics were performed using a two-way analysis of variance (ANOVA) followed by Dunnett and Tukey’s multiple comparison tests by means of the Prism 8.0 software (GraphPad, San Diego, CA, USA). Results are presented as mean values ± standard deviations. Values of *p* ≤ 0.05 were considered statistically significant.

## Supplementary Material





## Notes

Figure S1: minimized structure of compound **7** in complex with hCAs I, II, IX, and XII; Figure S2: ligand RMSD analysis during the initial MD simulations; Figure S3: ligand RMSD analysis during the extended MD simulations; Figure S4: predicted hCA IX/XII-**7** complexes superimposed to hCA I/II structures; and Figures S5–S58: ^1^H and ^13^C NMR of the newly synthesized compounds (PDF)
Molecular formula strings (CSV)

## Abbreviations

AAZ: acetazolamide
ACN: acetonitrile
ALK: anaplastic lymphoma kinase
AM1-BCC: Austin Model 1-bond charge correction
AMBER: assisted model building with energy refinement
ANOVA: analysis of variance
ATCC: American Type Culture Collection
A549: human lung adenocarcinoma cell line
BEAS-2B: human bronchial epithelial cell line
CA: carbonic anhydrase
CAMK4: calcium/calmodulin-dependent protein kinase IV
CK2: casein kinase 2
CTRL: control
CuAAC: copper-catalyzed azide–alkyne cycloaddition
DAPI: 4′,6-diamidino-2-phenylindole
DMF: *N*,*N*-dimethylformamide
DMSO: dimethyl sulfoxide
EGFR: epidermal growth factor receptor
EMT: epithelial-to-mesenchymal transition
EpCAM: epithelial cell adhesion molecule
FBS: fetal bovine serum
ff14SB: force field 14SB
GAFF: general AMBER force field
Hepes: 4-(2-hydroxyethyl)-1-piperazineethanesulfonic acid
h: human
HMR: hydrogen mass repartition
IL: interleukin
KI: inhibition constant
LDH: lactate dehydrogenase
LPS: lipopolysaccharide
MTA: pemetrexed
MARK4: microtubule affinity-regulating kinase 4
MD: molecular dynamics
MTT: 3-(4,5-dimethylthiazol-2-yl)-2,5-diphenyltetrazolium bromide
NF-κB: nuclear factor kappa-light-chain-enhancer of activated B cells
NMR: nuclear magnetic resonance
NSCLC: nonsmall cell lung cancer
PDB: Protein Data Bank
PI: propidium iodide
Pgp: P-glycoprotein
PLP: piecewise linear potential
PME: particle mesh Ewald
RMSD: root-mean-square deviation
ROS1: c-ros oncogene 1 receptor tyrosine kinase
SAR: structure–activity relationship
TIP3P: transferable intermolecular potential with 3 points
TLC: thin-layer chromatography
TNF-α: tumor necrosis factor alpha
TRPV1: transient receptor potential vanilloid type 1
UMB: umbelliferon
Van: vanillin.
