# Supplementary material for: Hybridization Approach Applied to Umbelliferon and Vanilloids toward New Inhibitors of Carbonic Anhydrases IX and XII with In Vitro Antiproliferative and Anti-inflammatory Activities
Source: J Med Chem. 2026 Jan 8;69(2):1454–72. doi: 10.1021/acs.jmedchem.5c02930 (PMC12833850; doi:10.1021/acs.jmedchem.5c02930)
Supplement: Supplementary file 1 [file jm5c02930_si_001.pdf]

Supporting Information

Hybridization Approach Applied to Umbelliferon and Vanilloids Towards New Inhibitors of Carbonic Anhydrases IX and XII with *in vitro* Antiproliferative and Anti-inflammatory Activities

Francesco Melfi[a,‖], Noemi Mencarelli[a,‖], Simone Carradori[a,e,*], Marialucia Gallorini[a,*], Andrea Angeli[b], Giulio Poli[c], Amelia Cataldi[a,e], Ilaria D'Agostino[c], Andrea Di Credico[d,e], Angela Di Baldassarre[d,e], Tiziano Tuccinardi[c], Claudiu T. Supuran[b]

[a]Department of Pharmacy, "G. d'Annunzio" University of Chieti-Pescara, via dei Vestini 31, 66100 Chieti, Italy

[b]Department NEUROFARBA, Pharmaceutical and Nutraceutical Section, University of Firenze, via Ugo Schiff 6, 50019 Sesto Fiorentino, Firenze, Italy

[c]Department of Pharmacy, University of Pisa, Via Bonanno 6, 56126 Pisa, Italy

[d]Department of Medicine and Aging Sciences, "G. d'Annunzio", University of Chieti-Pescara, 66100, Chieti, Italy

[e]UdA-TechLab, "G. d'Annunzio", University of Chieti-Pescara, 66100, Chieti, Italy

*Corresponding authors: Prof. Simone Carradori, simone.carradori@unich.it, and Prof. Marialucia Gallorini, marialucia.gallorini@unich.it

‖These authors equally contributed

Table of Contents

Figure S1. Minimized structure of compound **7** in complex with hCAs I, II, IX, and XII **Page S2**

Figure S2. Ligand RMSD analysis during the initial MD simulations **Page S3**

Figure S3. Ligand RMSD analysis during the extended MD simulations **Page S3**

Figure S4. Predicted hCA IX/XII-**7** complexes superimposed to hCA I/II structures **Page S4**

Figures S5-S58. NMR spectra of the newly synthesized compounds **Pages S5-S31**

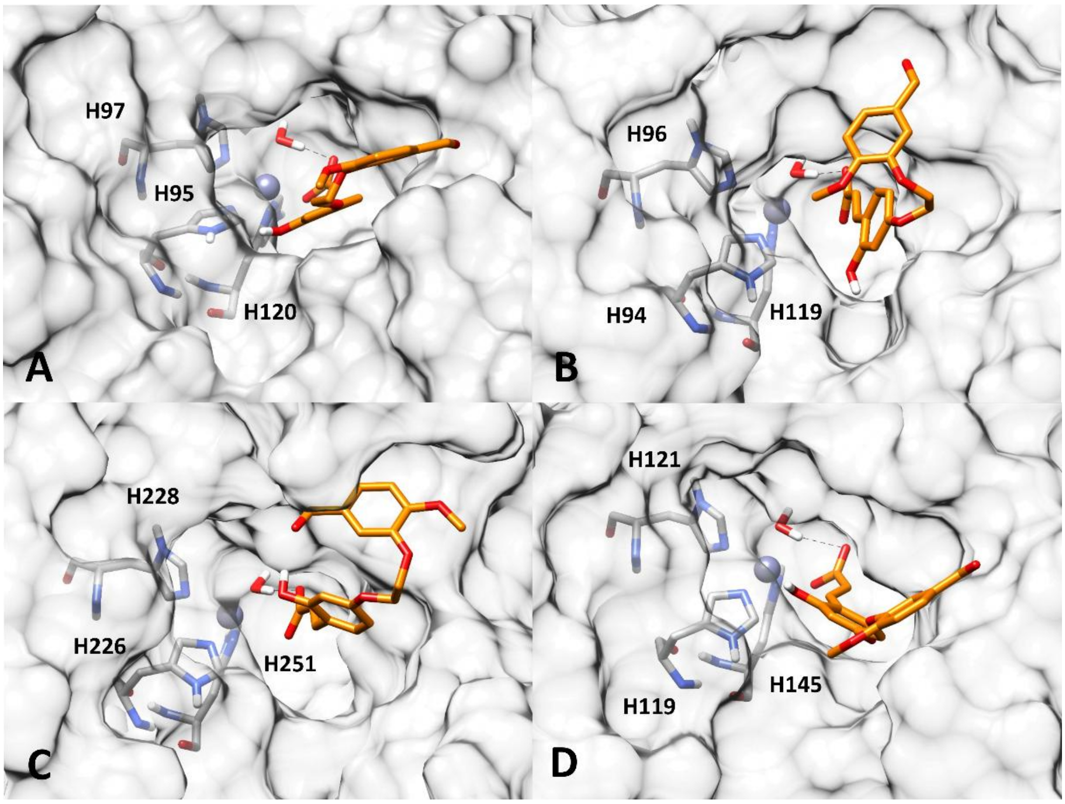

Figure S1. Energy-minimized structure of compound **7** in its hydrolyzed form (orange) in complex with hCA I (**A**), hCA II (**B**), hCA IX (**C**), and hCA XII (**D**). In all panels, the surface of the protein binding site is shown in transparency.

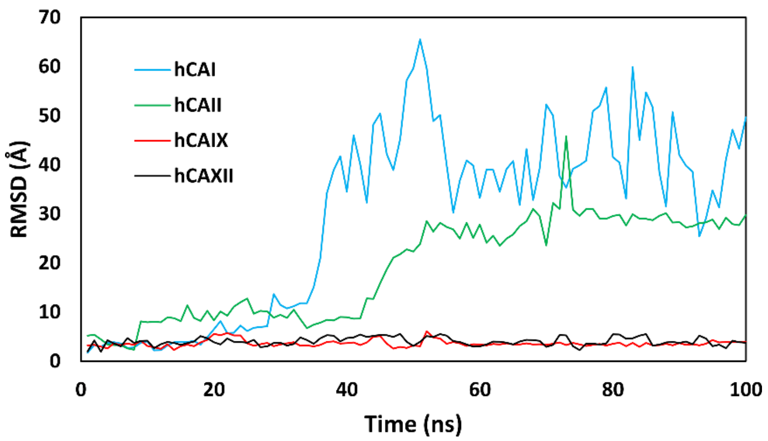

Figure S2. RMSD analysis of the ligand disposition, with respect to its initial coordinates, into the binding site of hCA I, II, IX, and XII during the initial MD simulations.

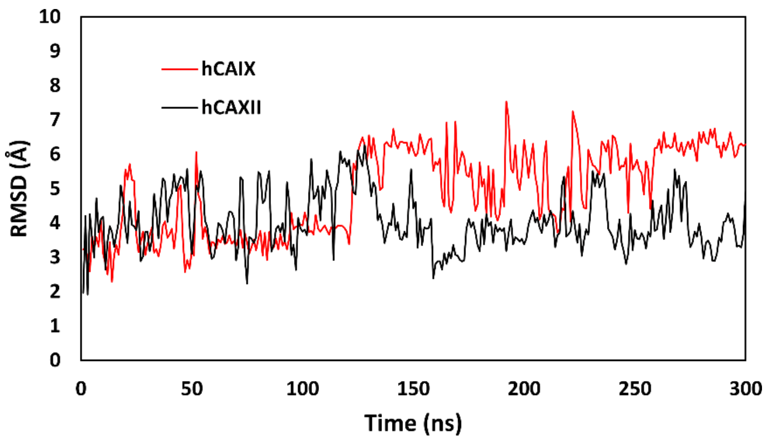

Figure S3. RMSD analysis of the ligand disposition, with respect to its initial coordinates, into the binding site of hCA IX, and XII during the extended MD simulations.

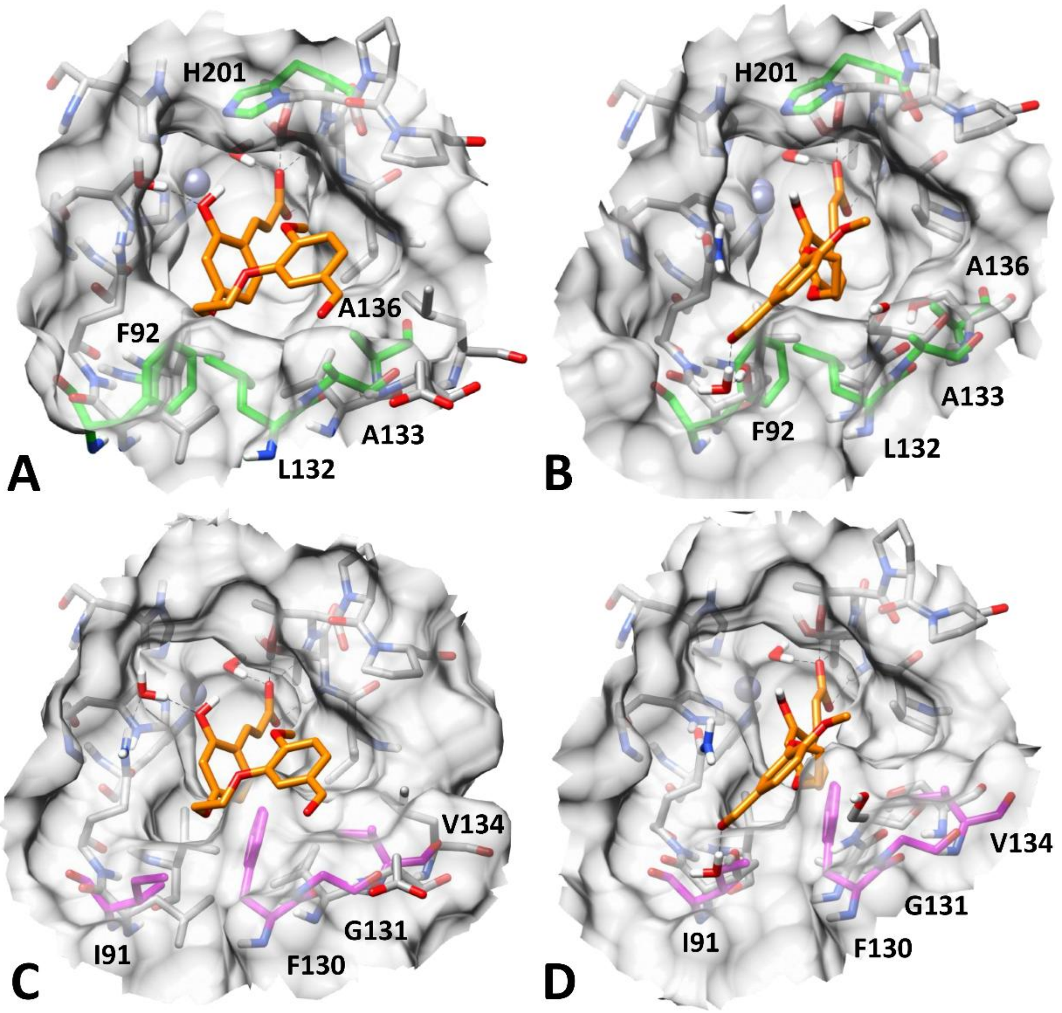

Figure S4. Minimized average structure of compound **7** in its hydrolyzed form (orange) in complex with hCA IX (**A**, **C**) and hCA XII (**B**, **D**), superimposed to the equilibrated structures of hCA I (**A**, **B**) and hCA II (**B**, **C**). The binding site residues of hCA IX and XII are shown in gray. Only non-conserved residues of hCA I and II are shown (in green and purple, respectively) and labelled. The binding site surfaces of hCA I and II are also shown in transparency.

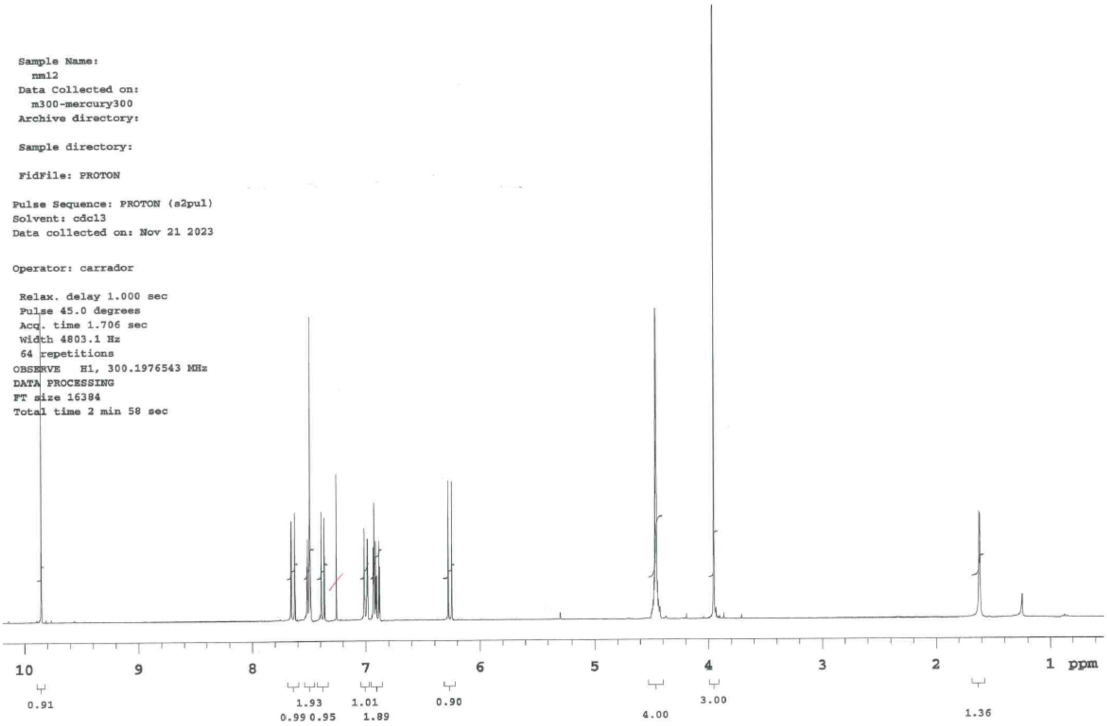

Figure S5. ^{1}H NMR of compound **7**.

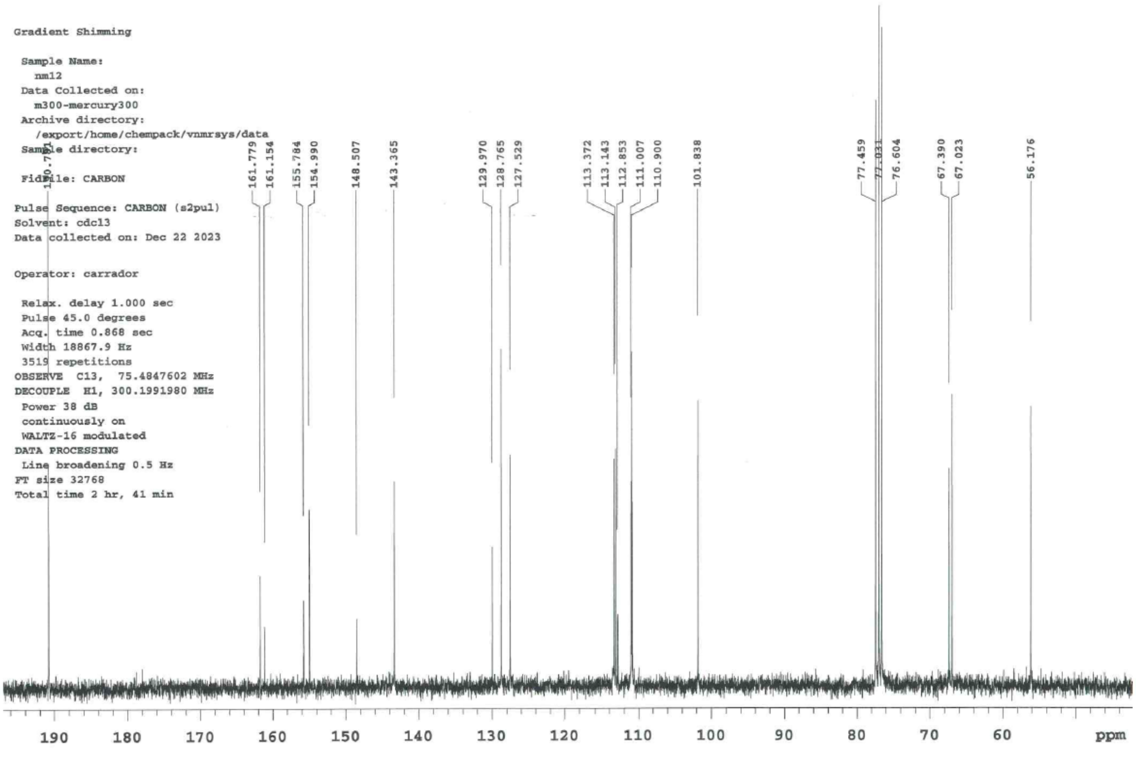

Figure S6. ^{13}C NMR of compound **7**.

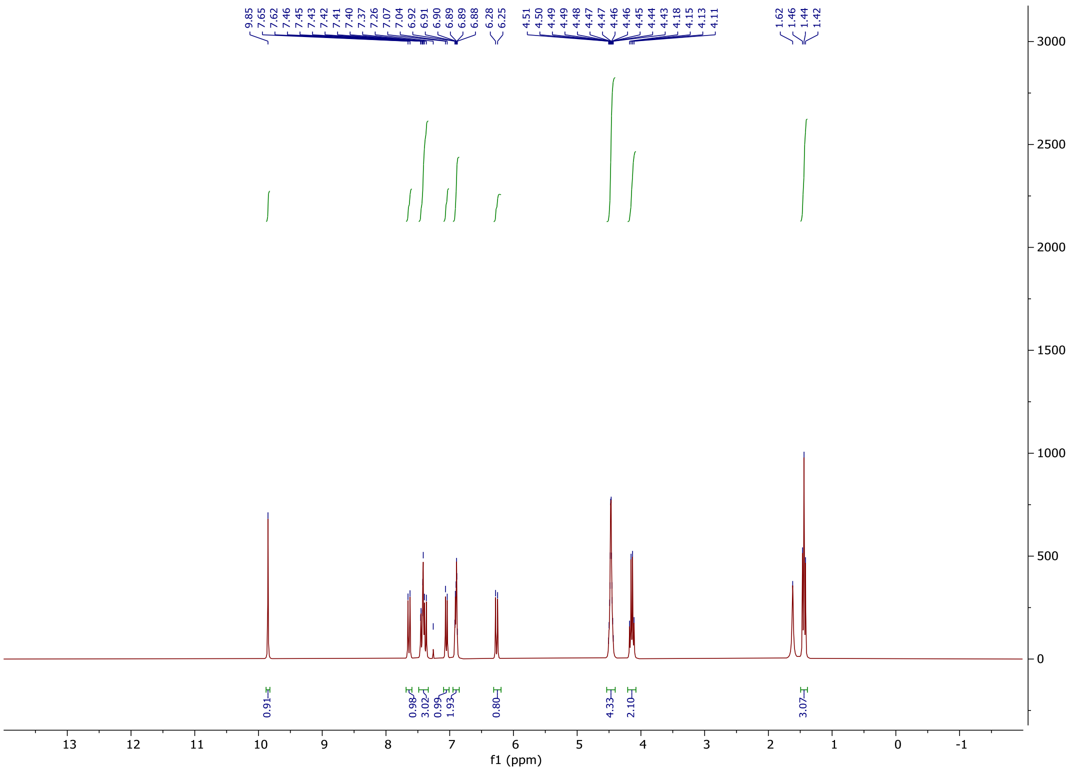

Figure S7. ^{1}H NMR of compound **8**.

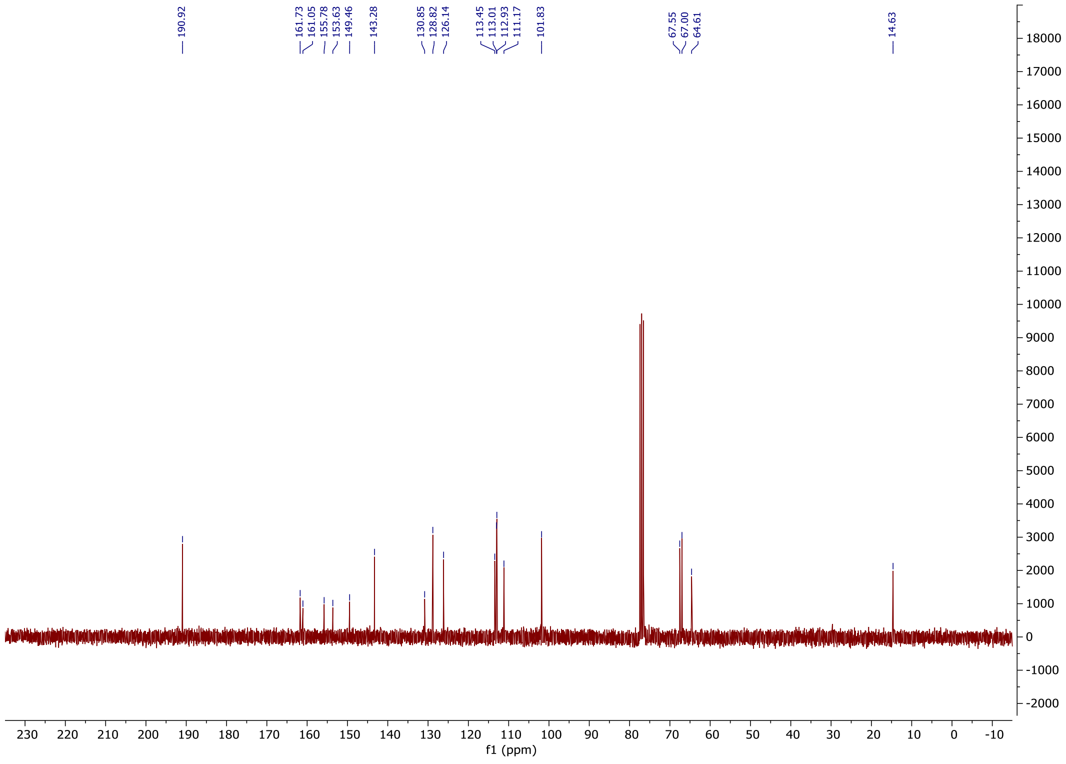

Figure S8. ^{13}C NMR of compound **8**.

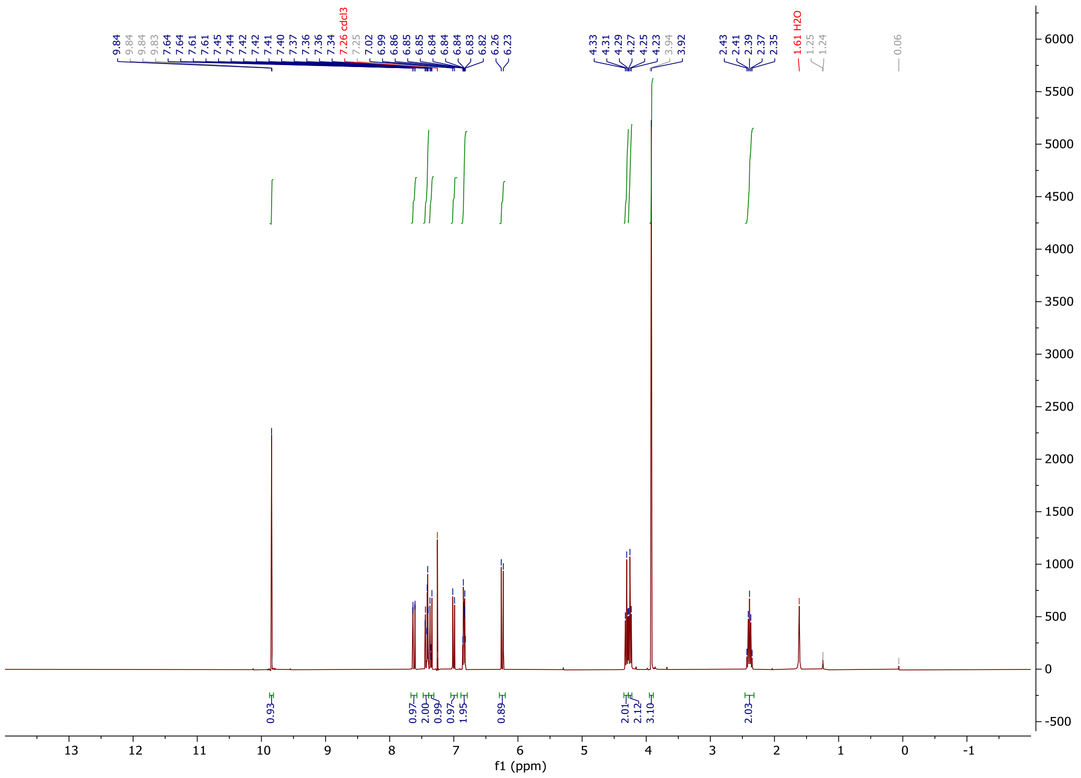

Figure S9. ^{1}H NMR of compound **9**.

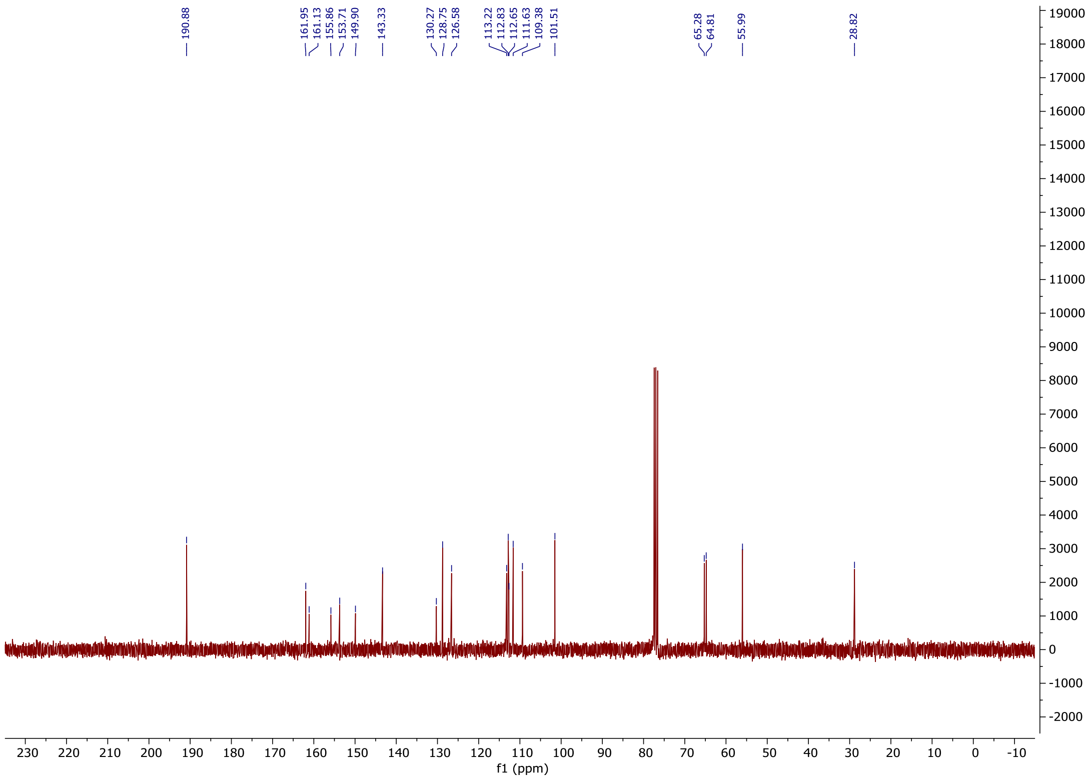

Figure S10. ^{13}C NMR of compound **9**.

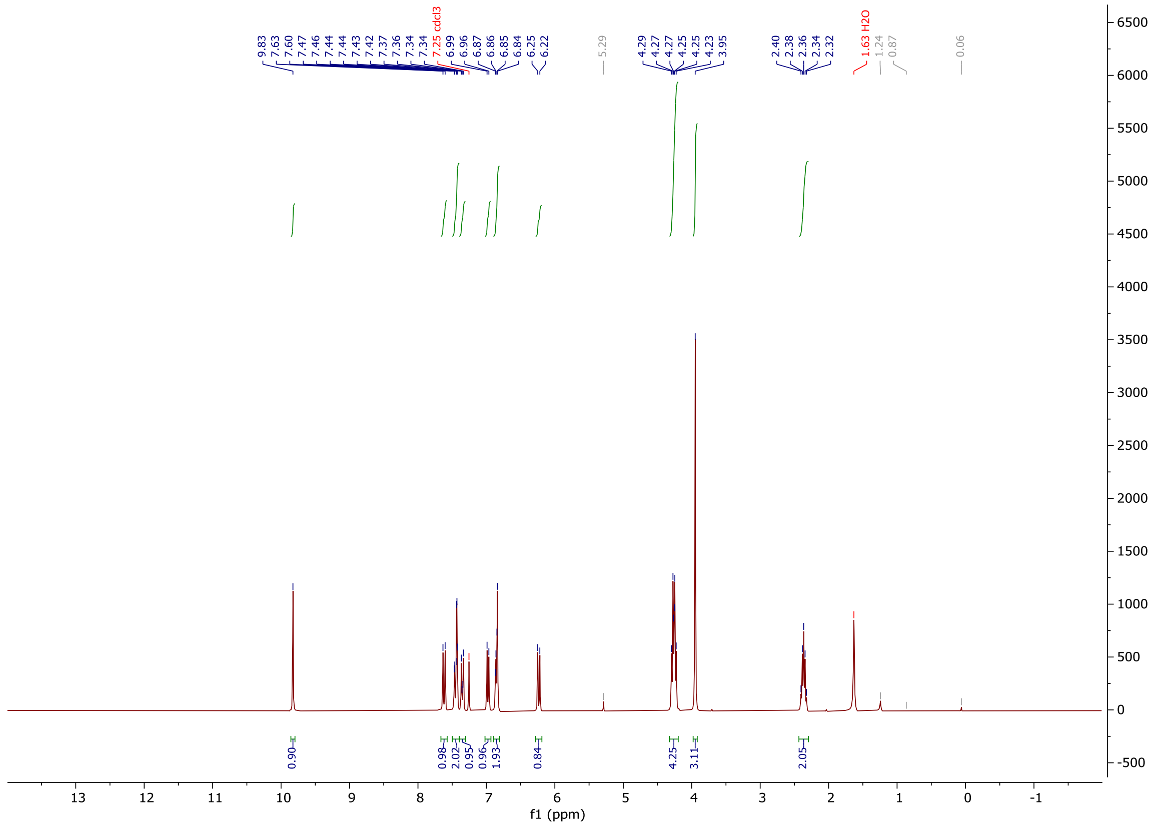

Figure S11. ^{1}H NMR of compound **10**.

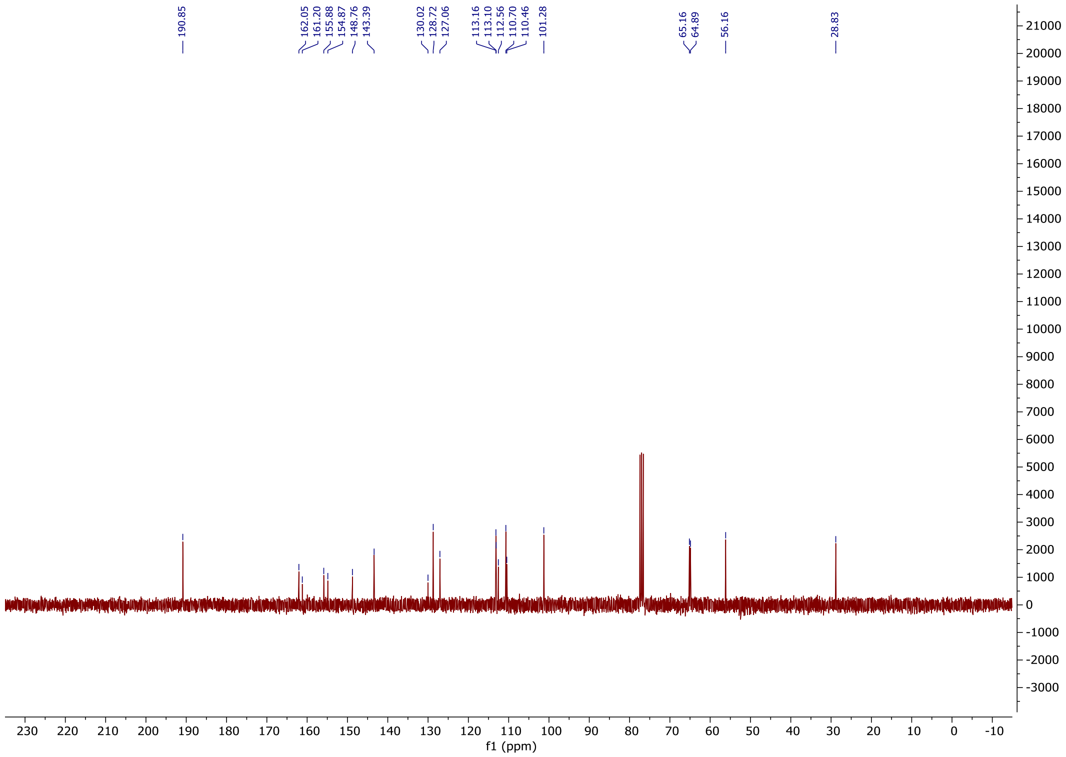

Figure S12. ^{13}C NMR of compound **10**.

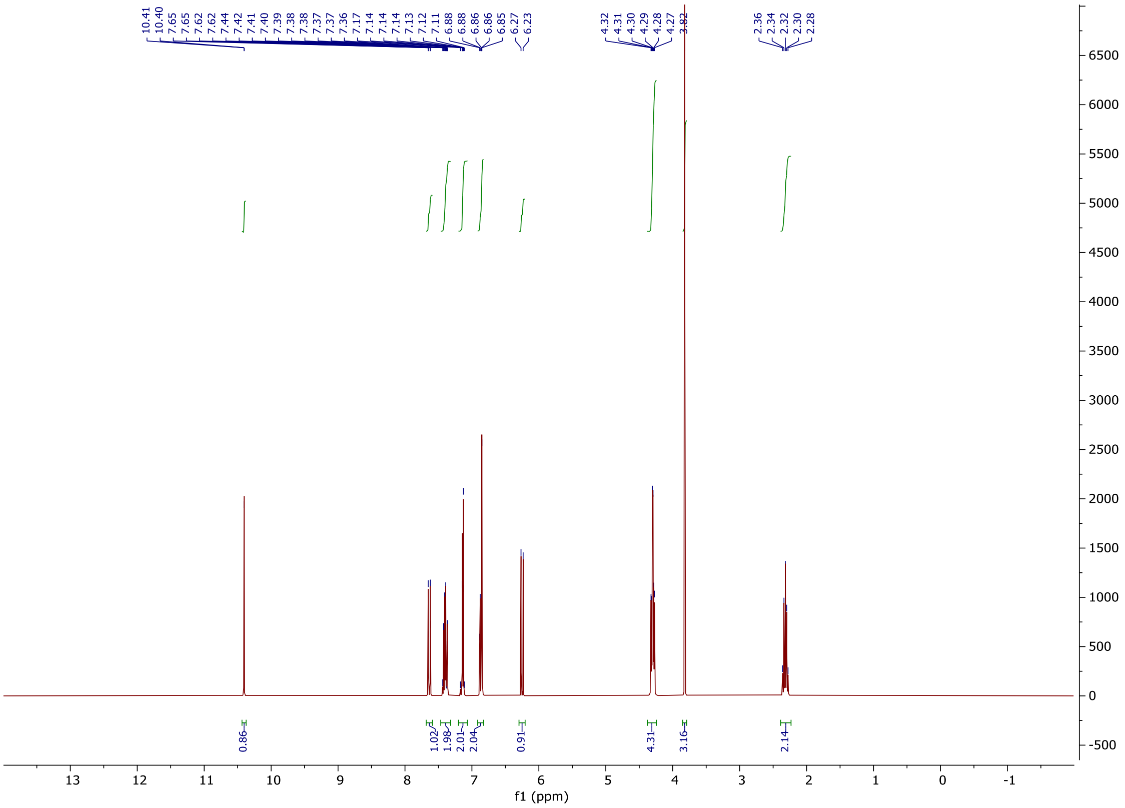

Figure S13. ^{1}H NMR of compound **11**.

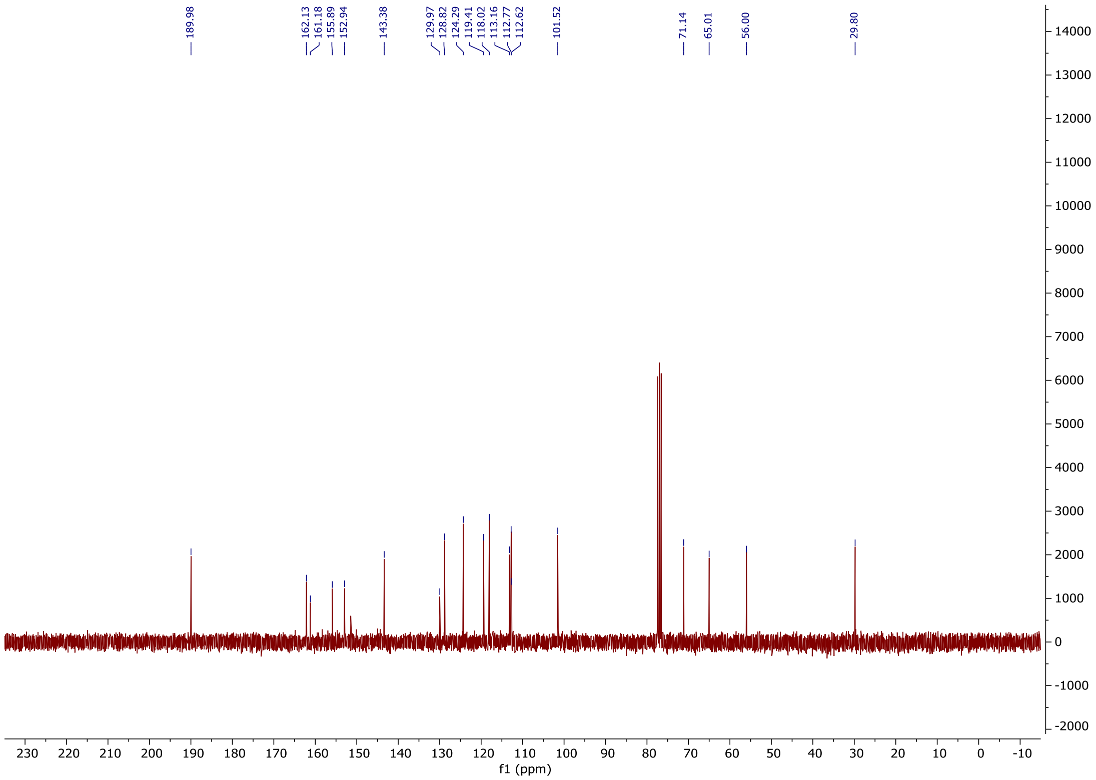

Figure S14. ^{13}C NMR of compound **11**.

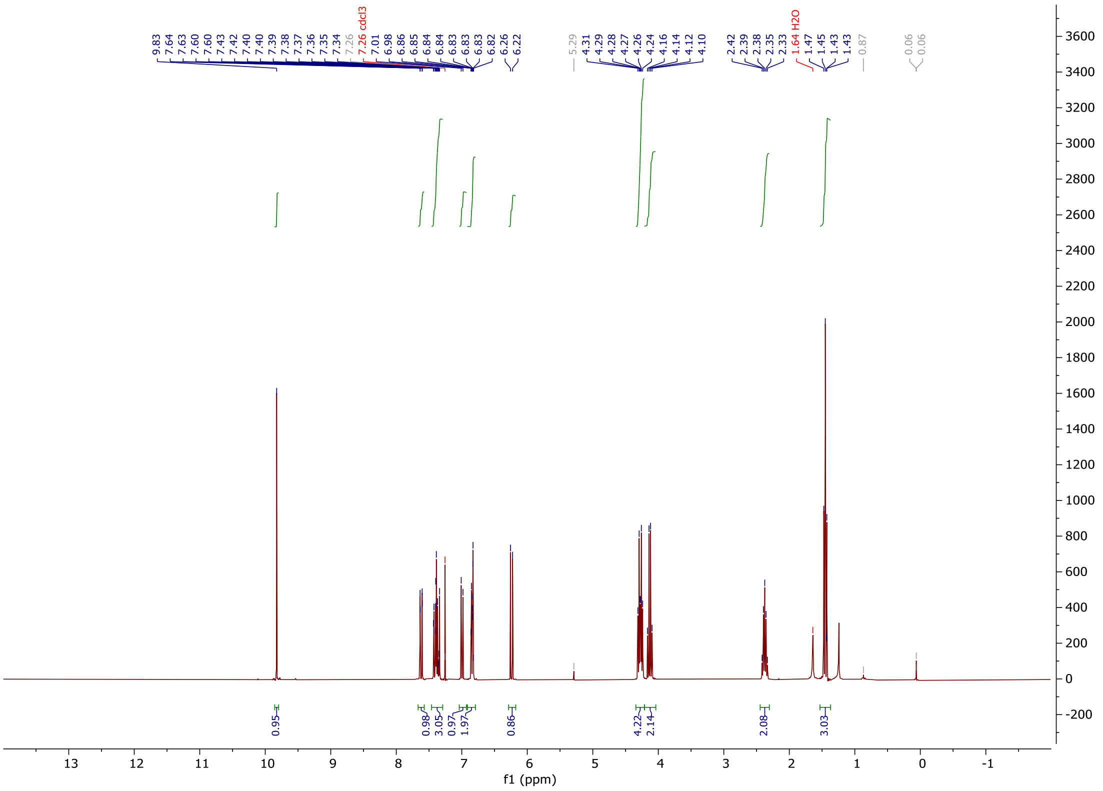

Figure S15. ^{1}H NMR of compound **12**.

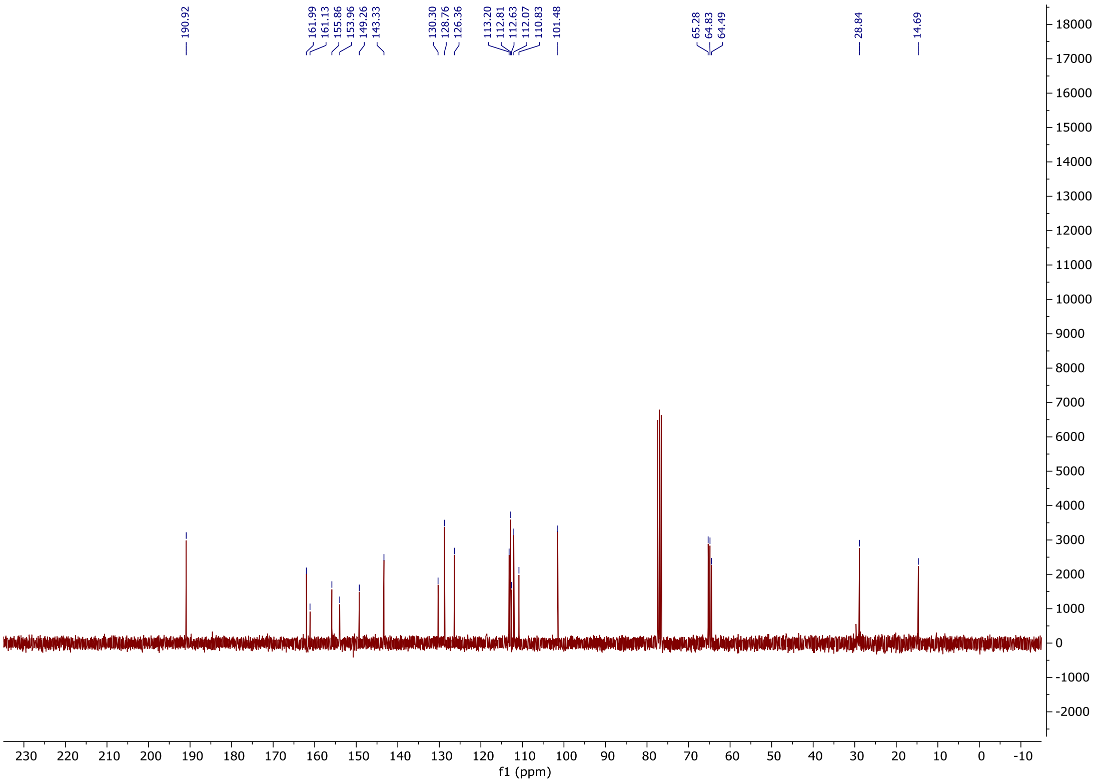

Figure S16. ^{13}C NMR of compound **12**.

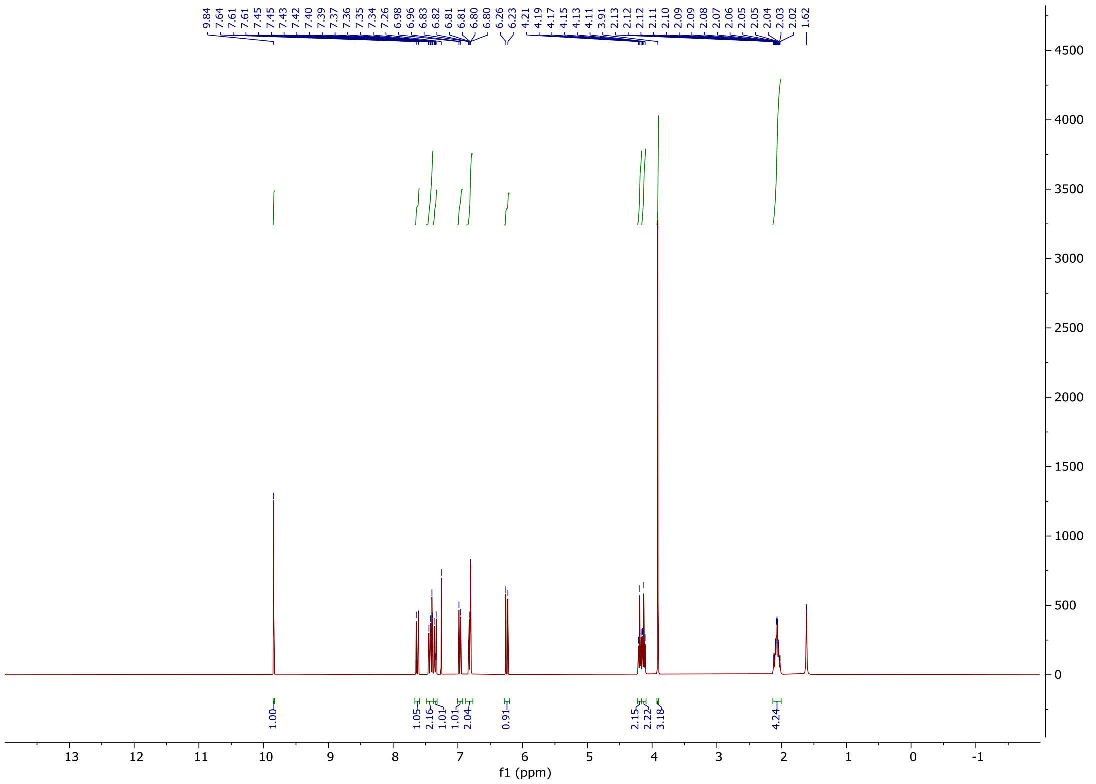

Figure S17. ^{1}H NMR of compound **13**.

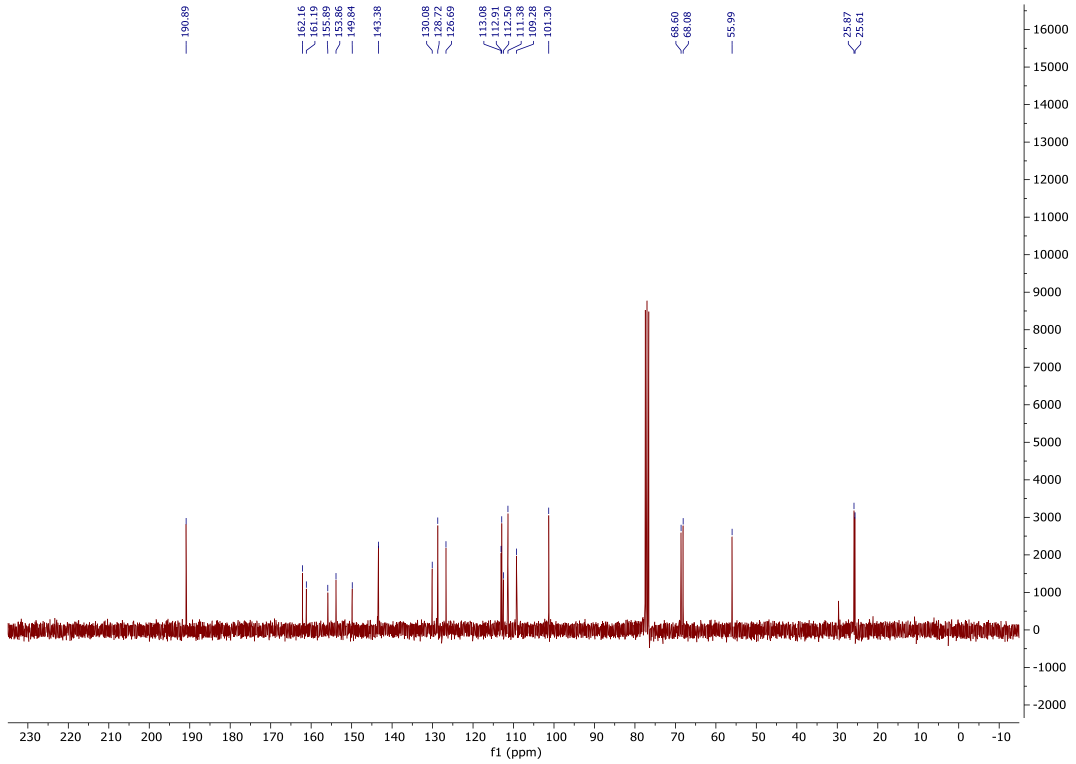

Figure S18. ^{13}C NMR of compound **13**.

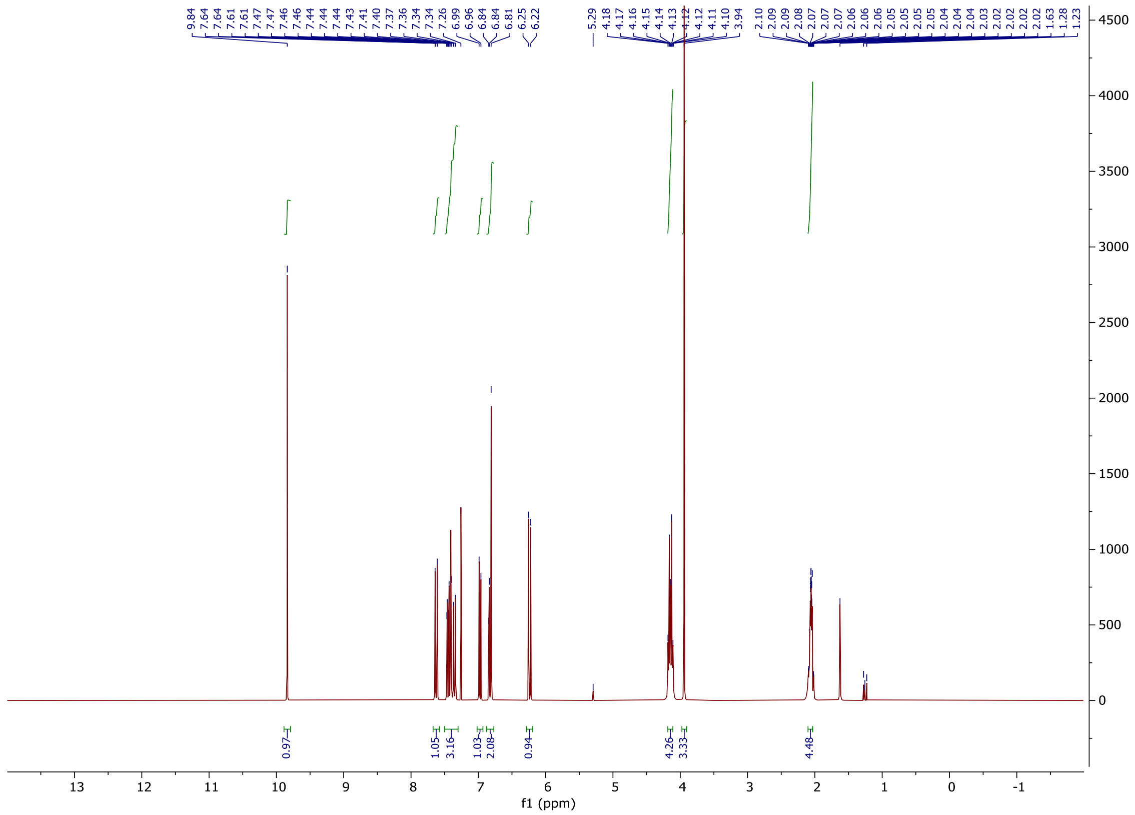

Figure S19. ^{1}H NMR of compound **14**.

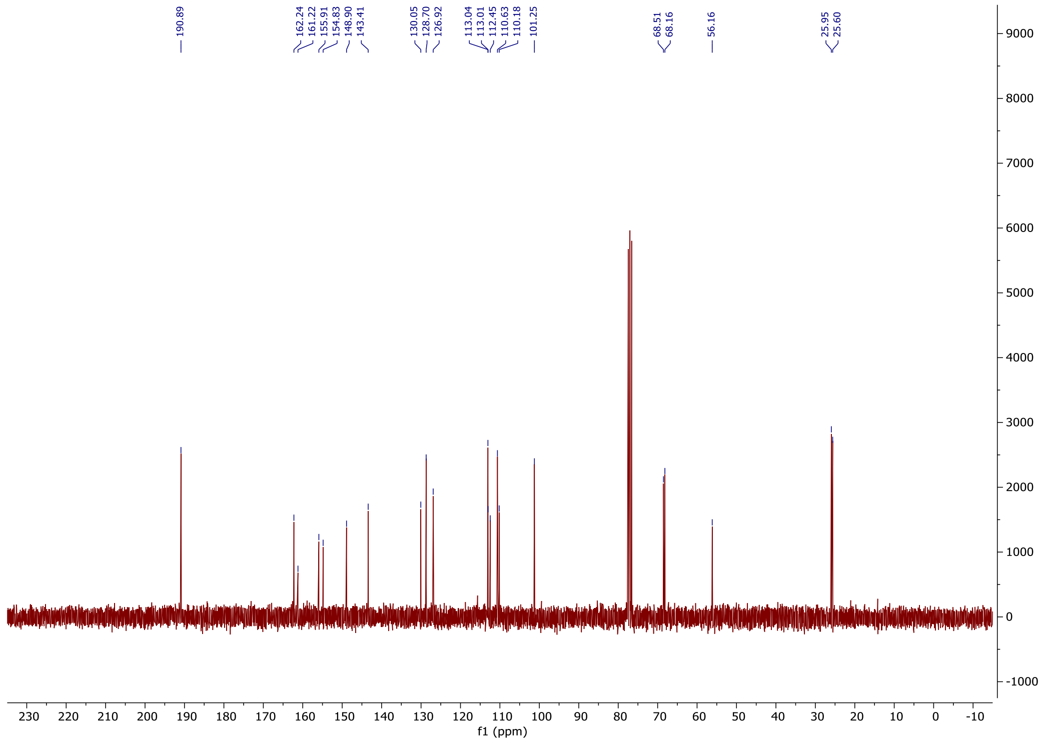

Figure S20. ^{13}C NMR of compound **14**.

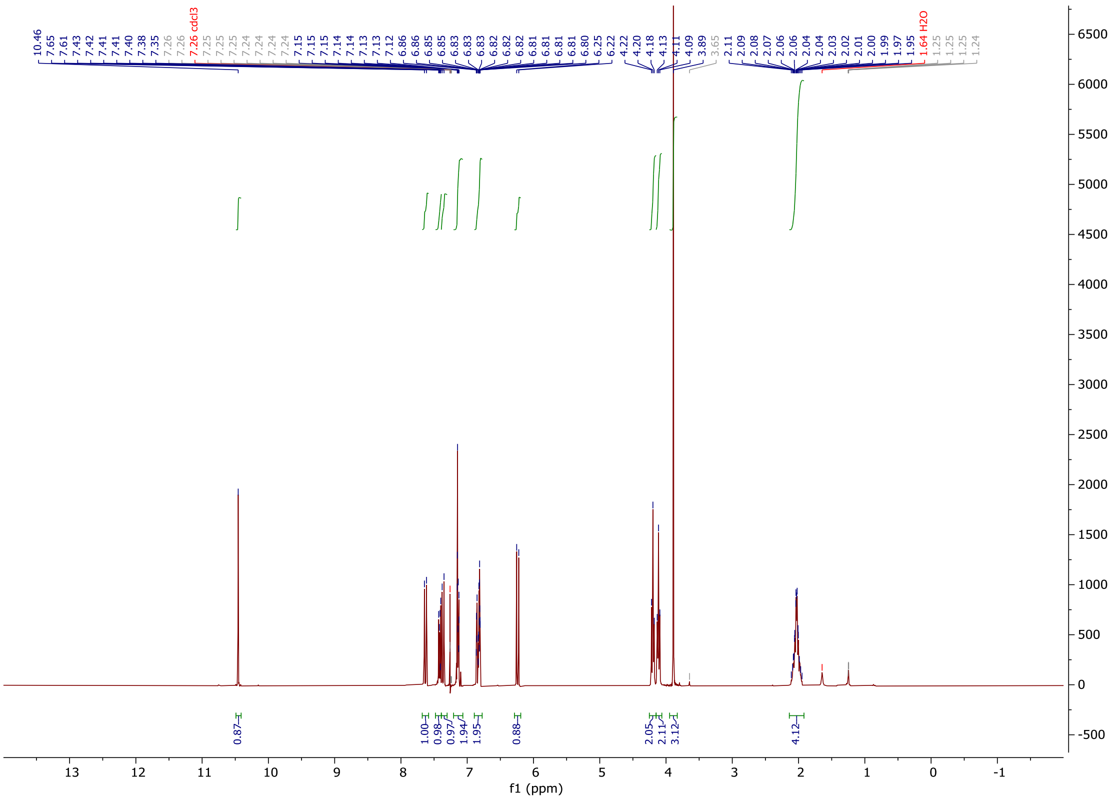

Figure S21. ^{1}H NMR of compound **15**.

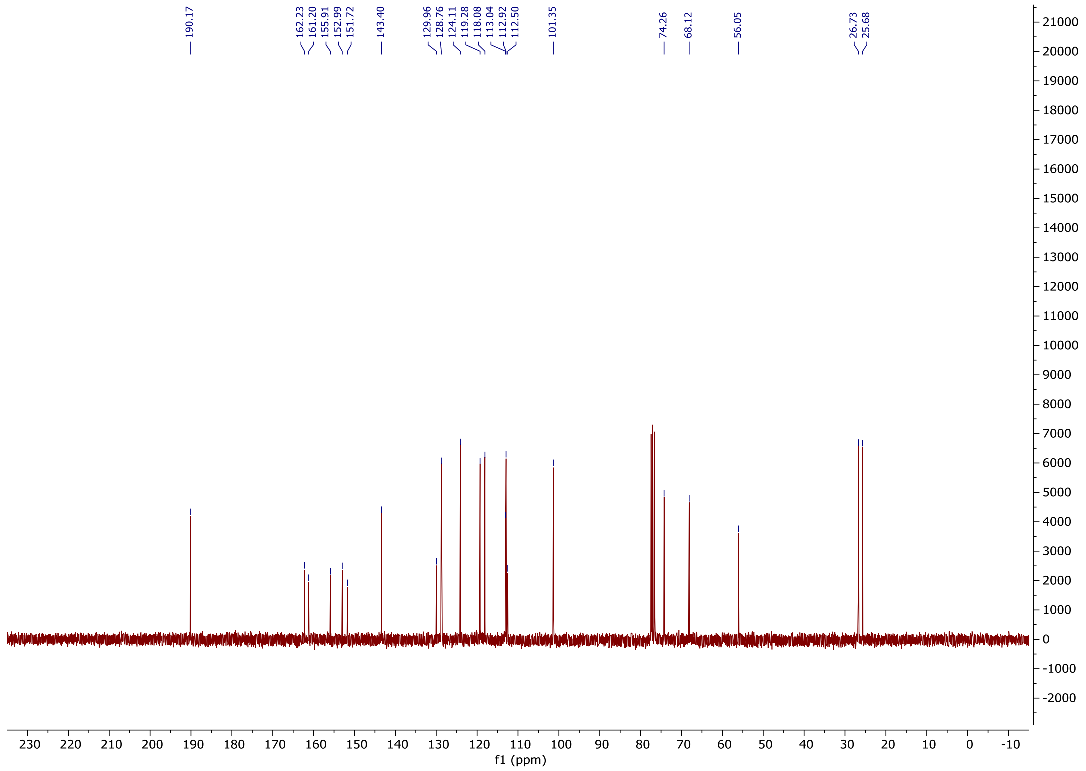

Figure S22. ^{13}C NMR of compound **15**.

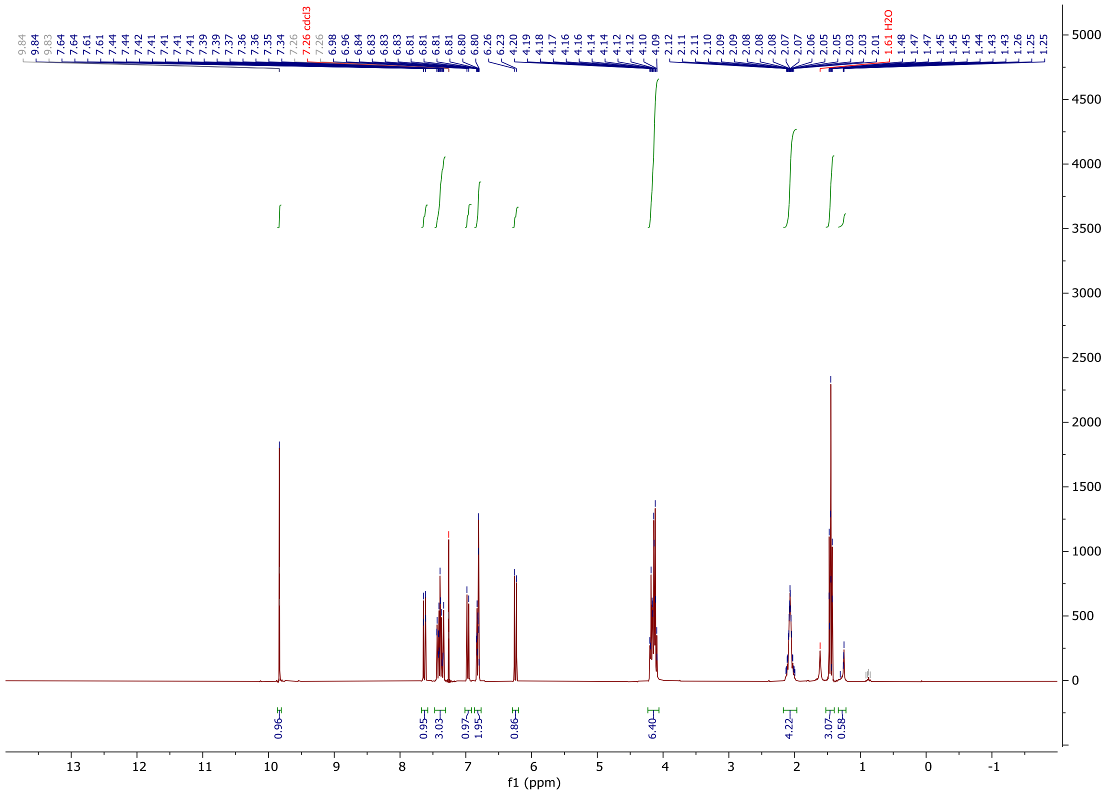

Figure S23. ^{1}H NMR of compound **16**.

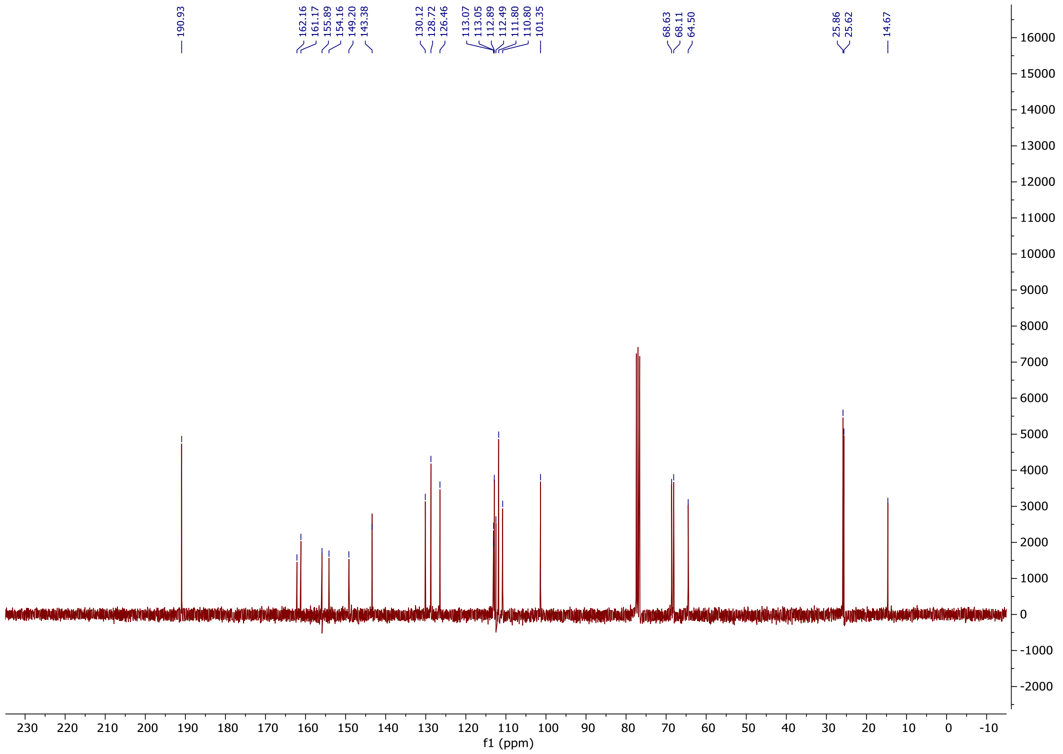

Figure S24. ^{13}C NMR of compound **16**.

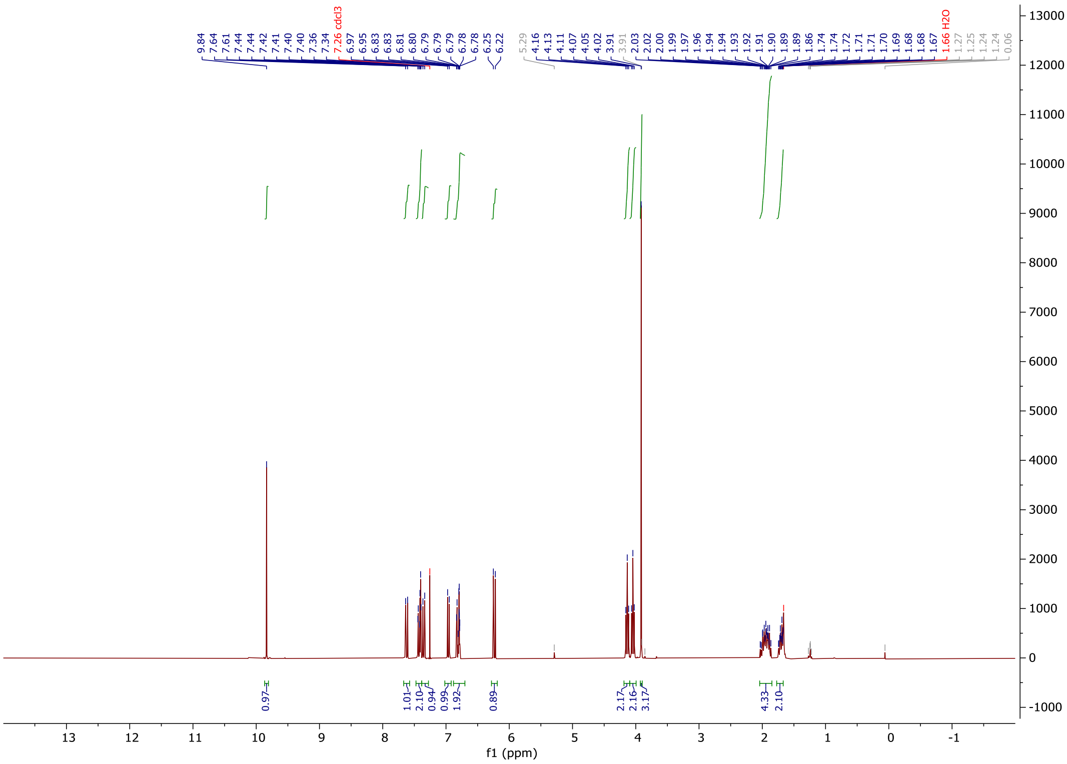

Figure S25. ^{1}H NMR of compound **17**.

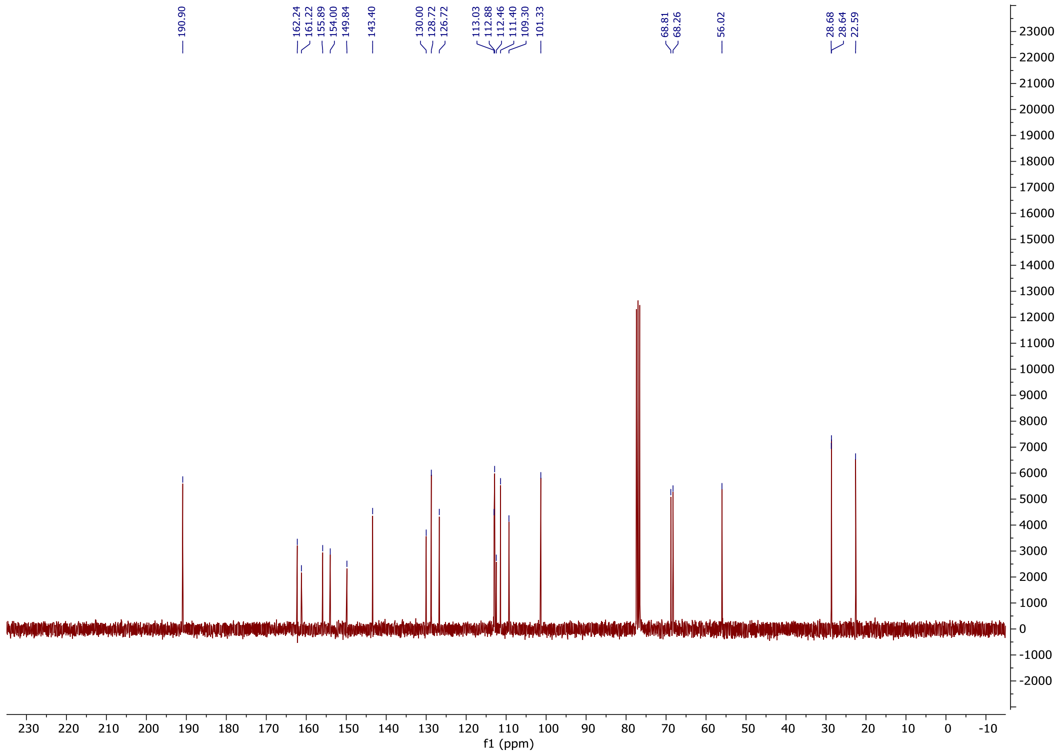

Figure S26. ^{13}C NMR of compound **17**.

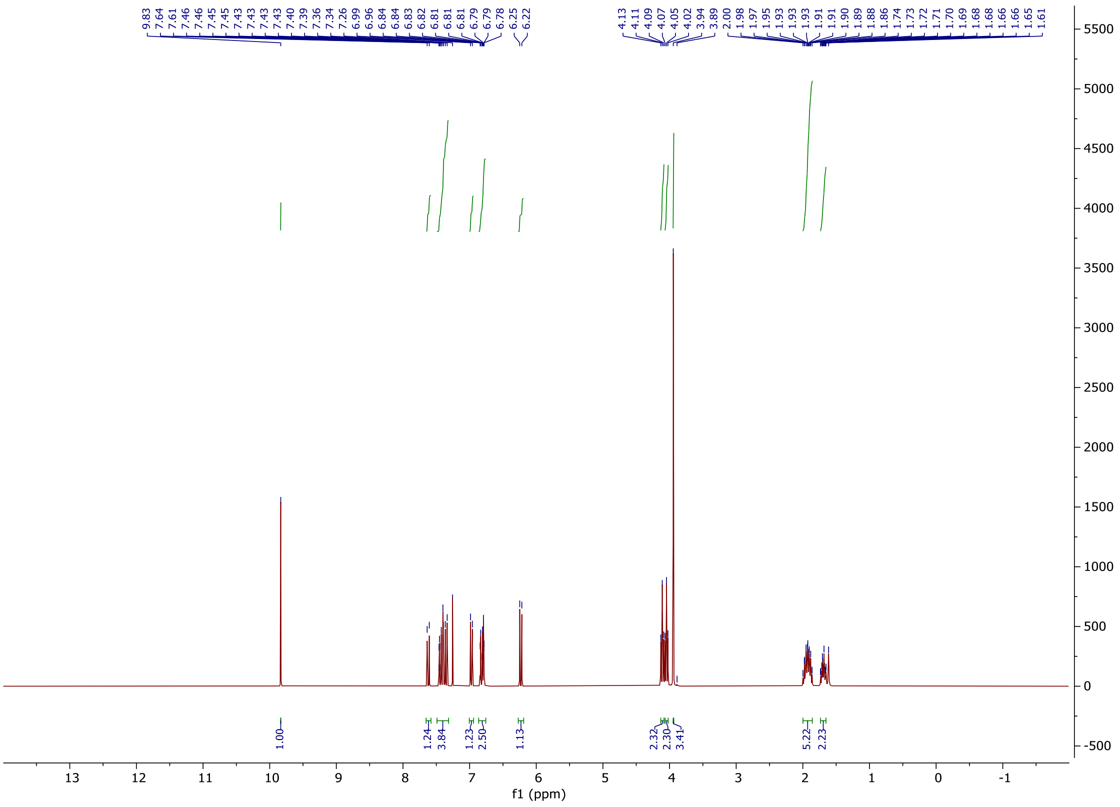

Figure S27. ^{1}H NMR of compound **18**.

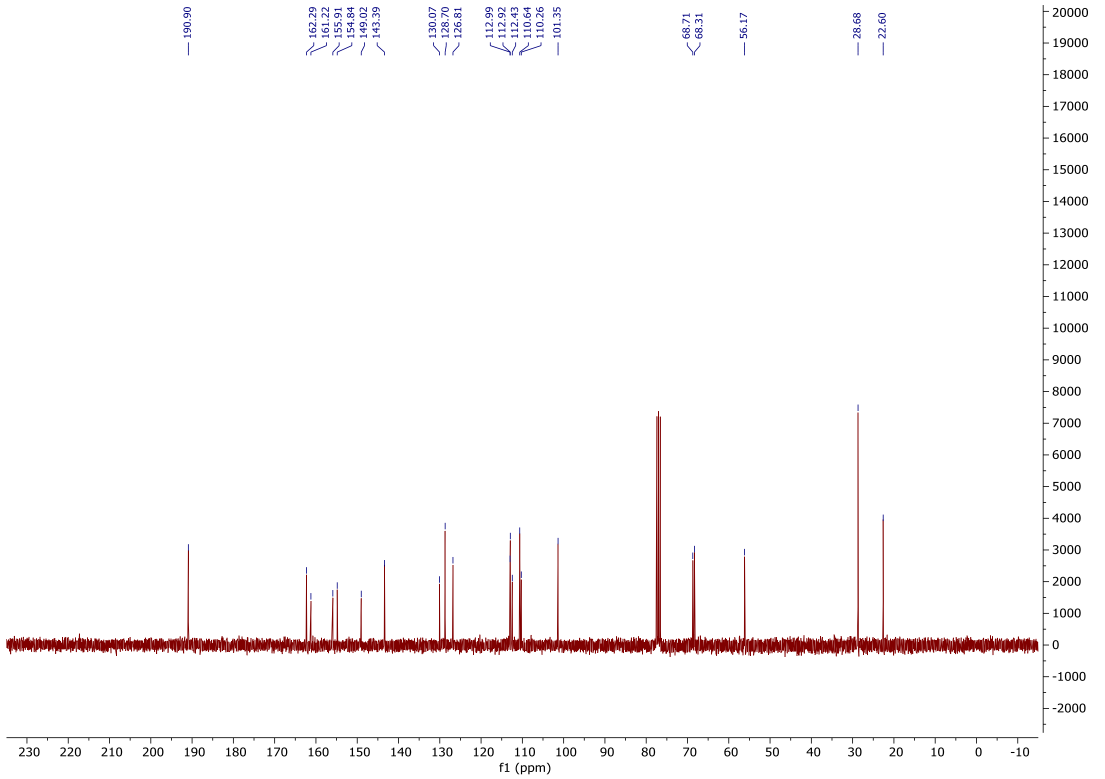

Figure S28. ^{13}C NMR of compound **18**.

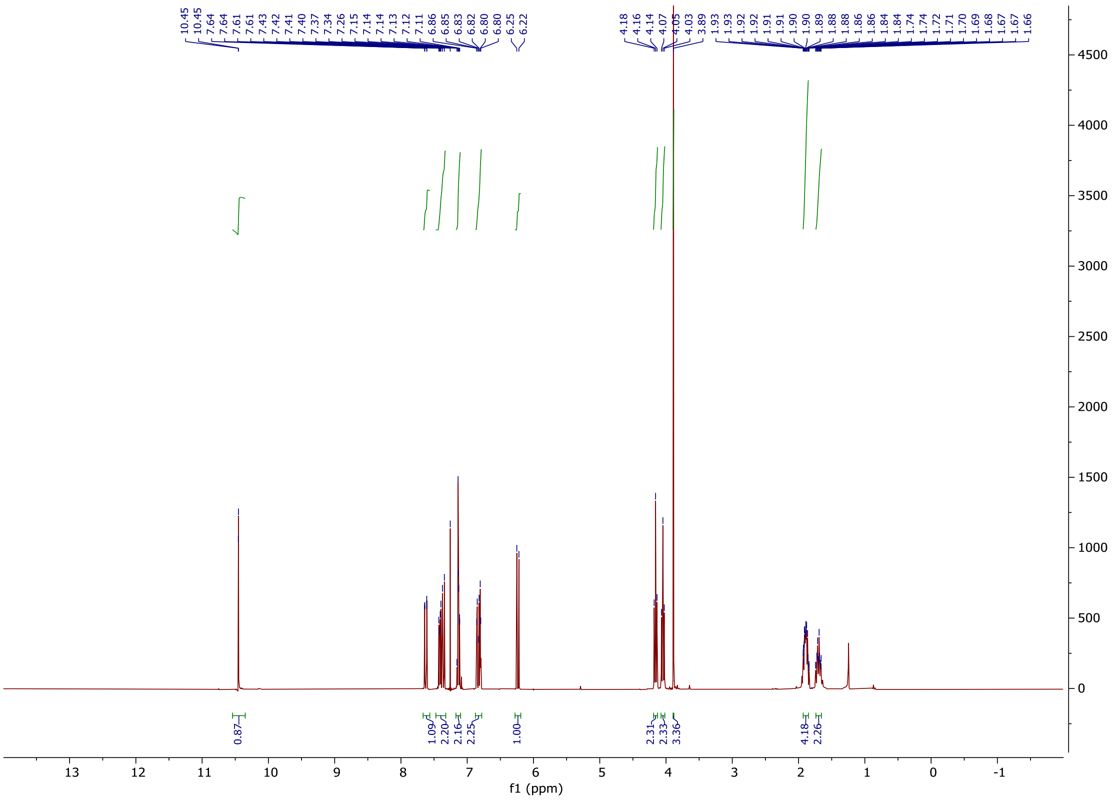

Figure S29. ^{1}H NMR of compound **19**.

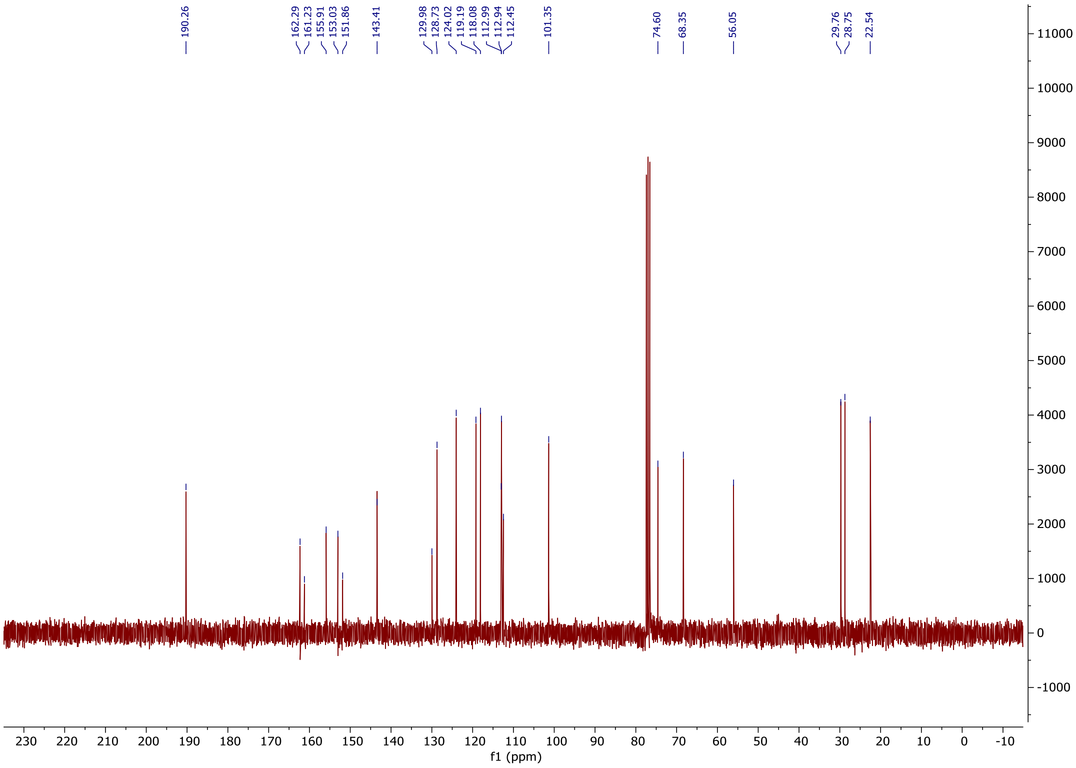

Figure S30. ^{13}C NMR of compound **19**.

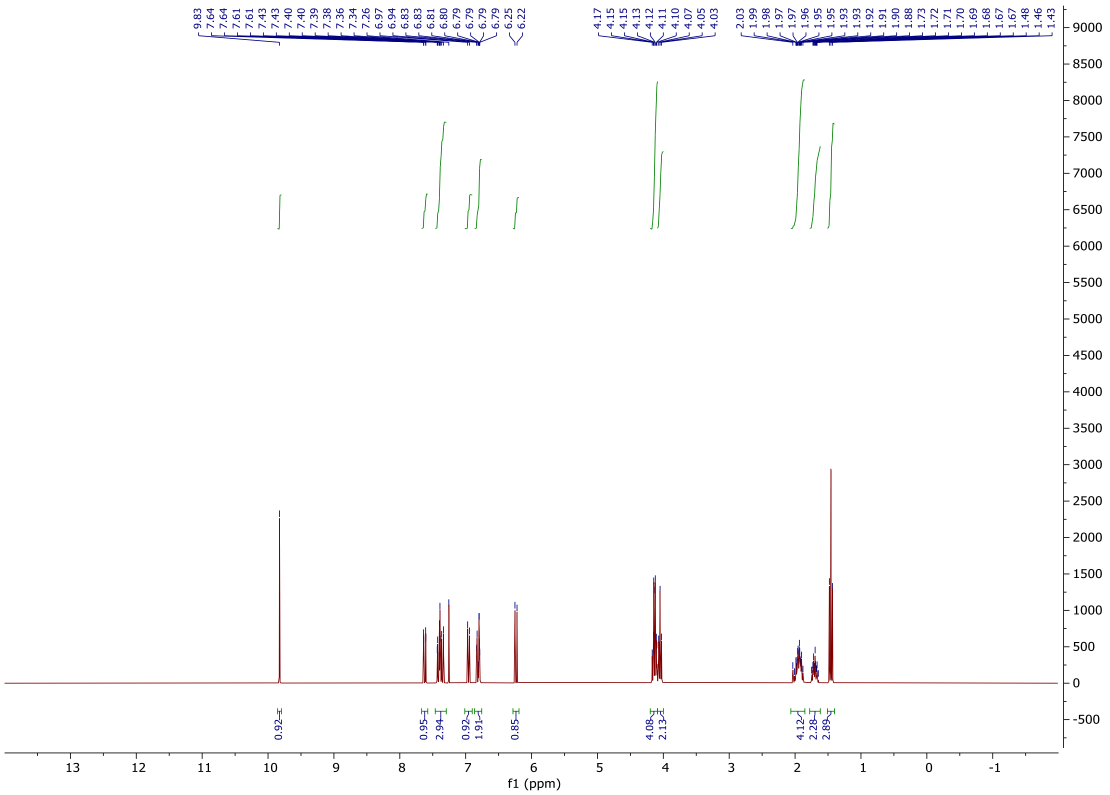

Figure S31. ^{1}H NMR of compound **20**.

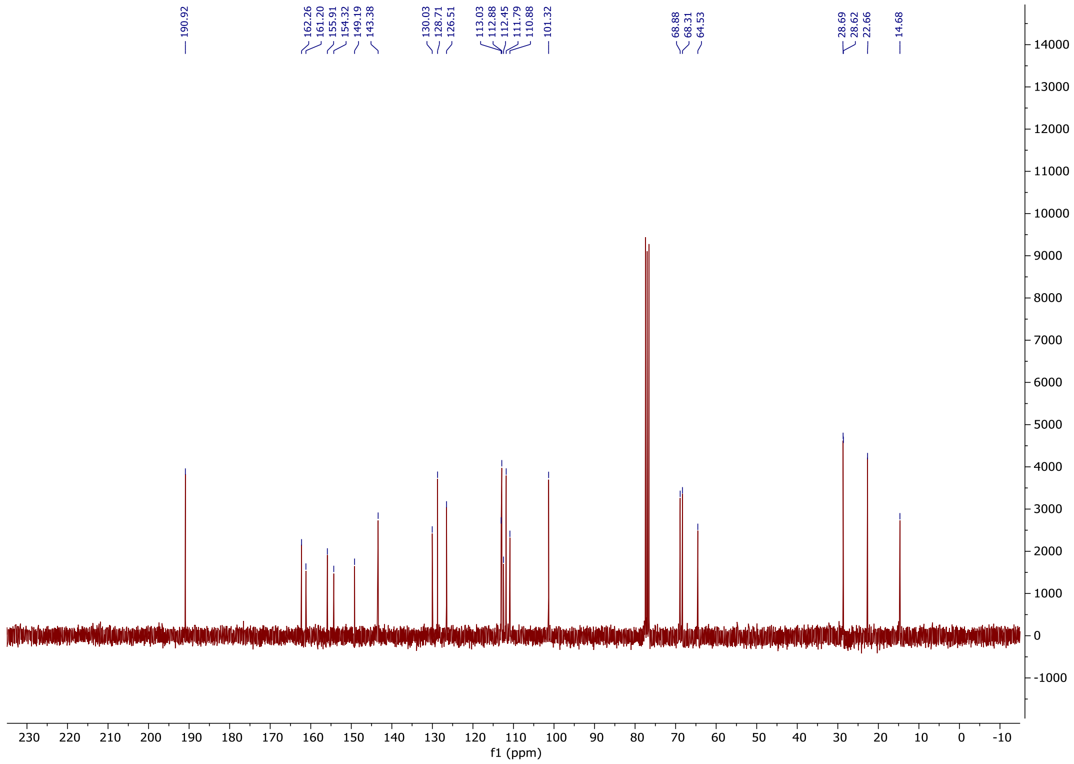

Figure S32. ^{13}C NMR of compound **20**.

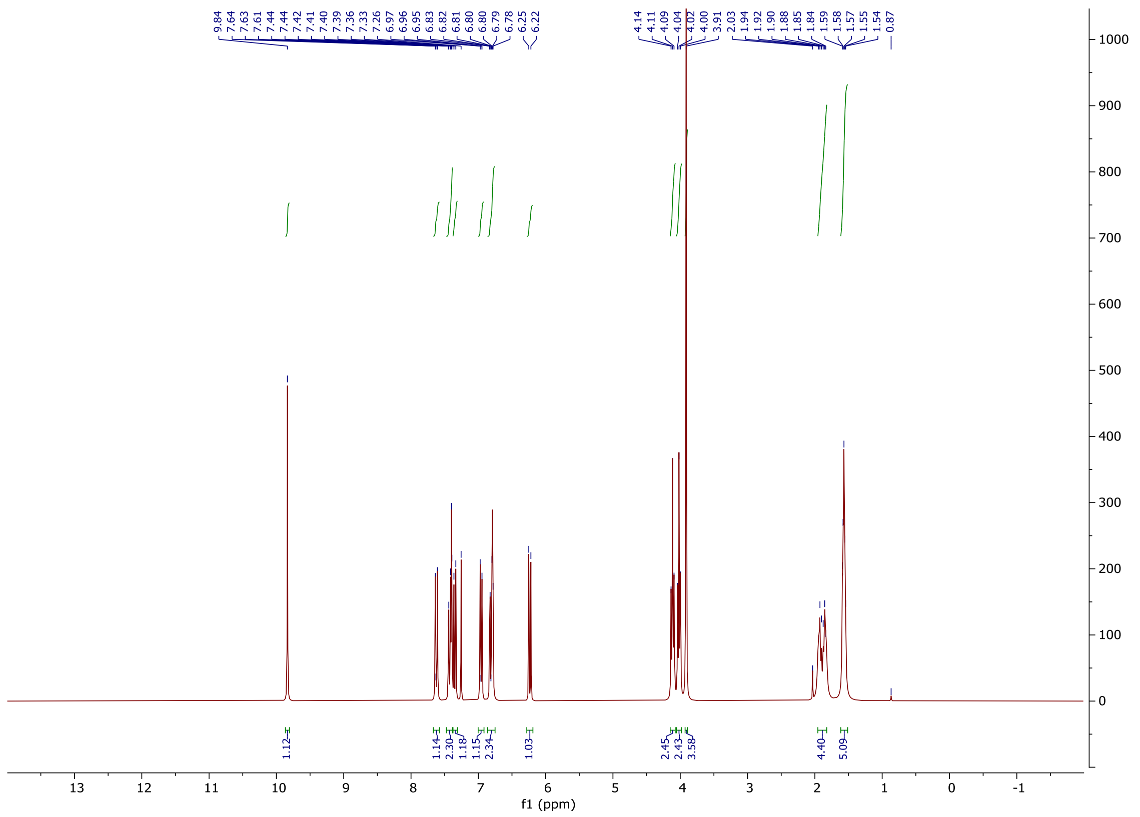

Figure S33. ^{1}H NMR of compound **21**.

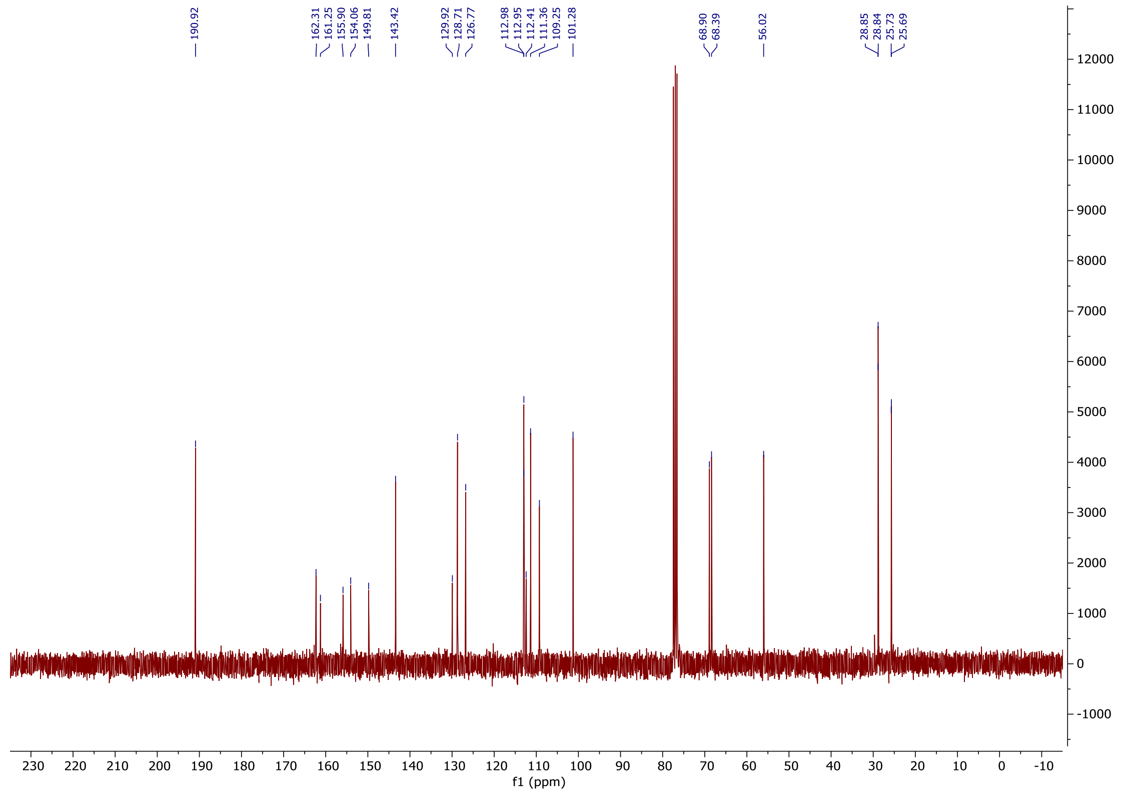

Figure S34. ^{13}C NMR of compound **21**.

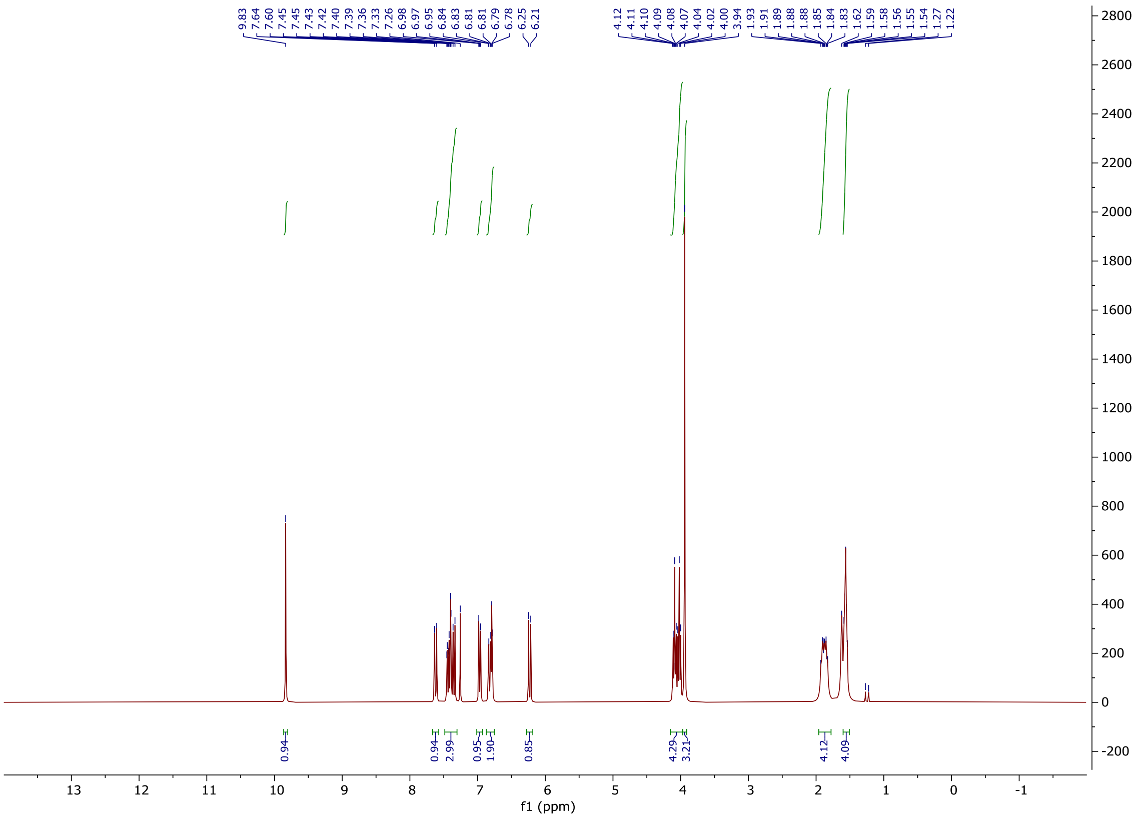

Figure S35. ^{1}H NMR of compound **22**.

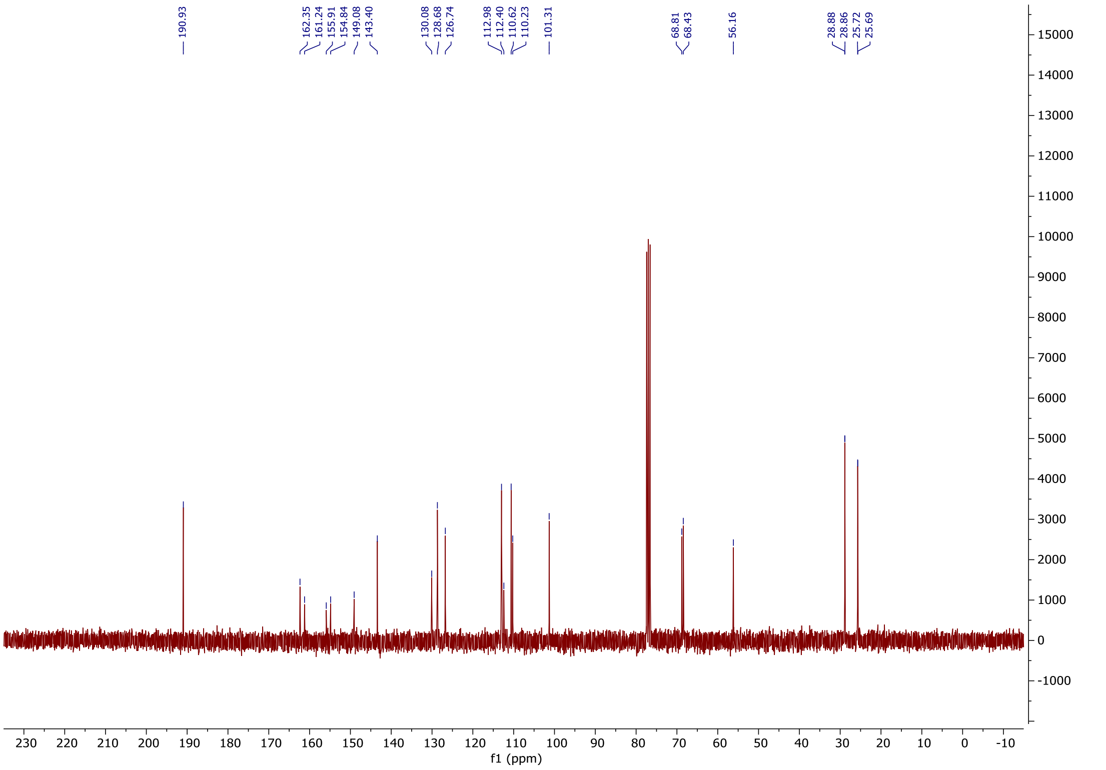

Figure S36. ^{13}C NMR of compound **22**.

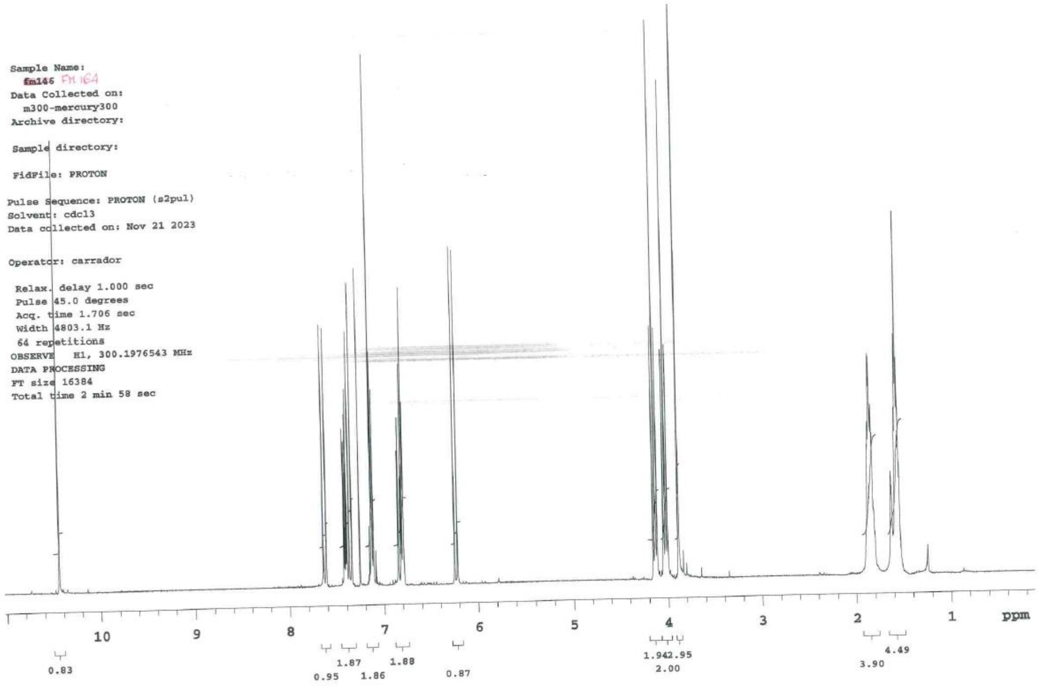

Figure S37. ^{1}H NMR of compound **23**.

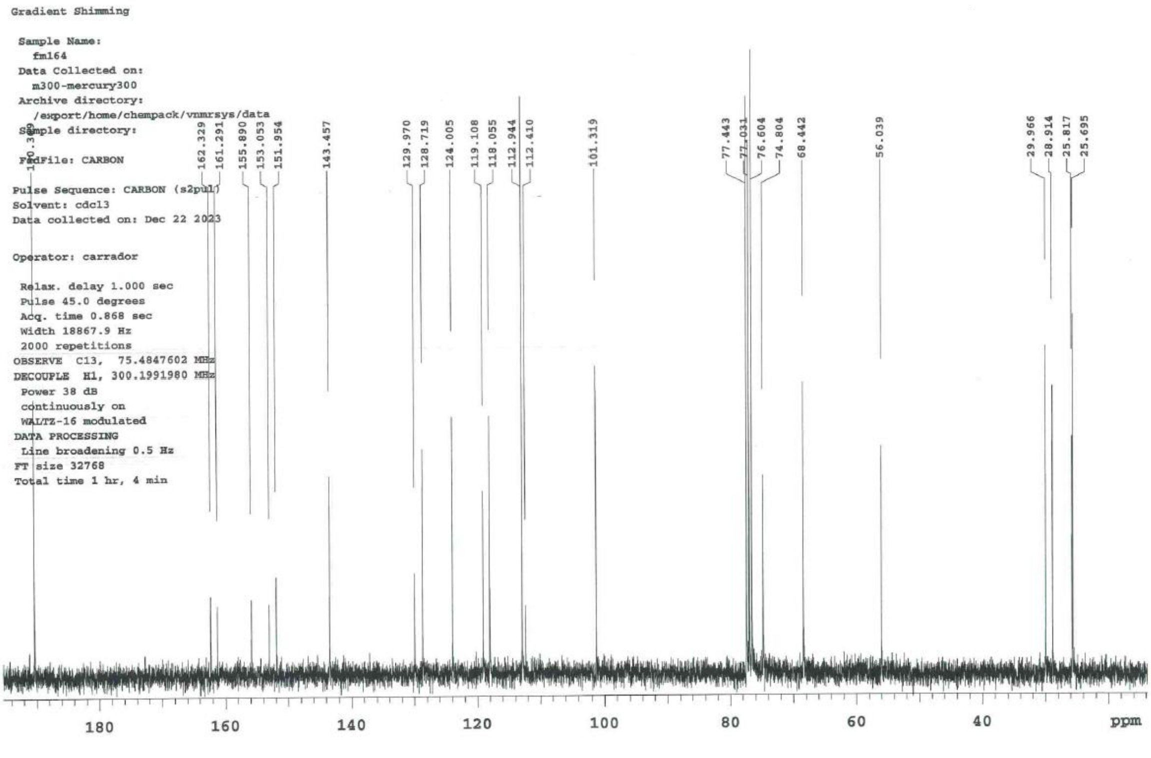

Figure S38. ^{13}C NMR of compound **23**.

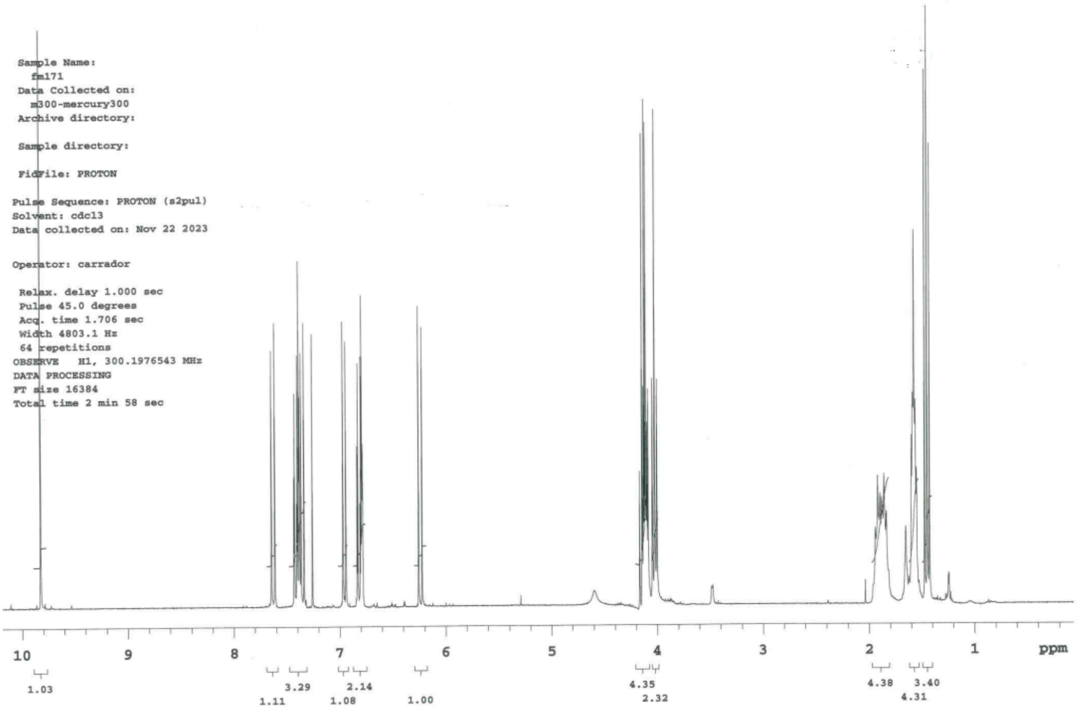

Figure S39. ^{1}H NMR of compound **24**.

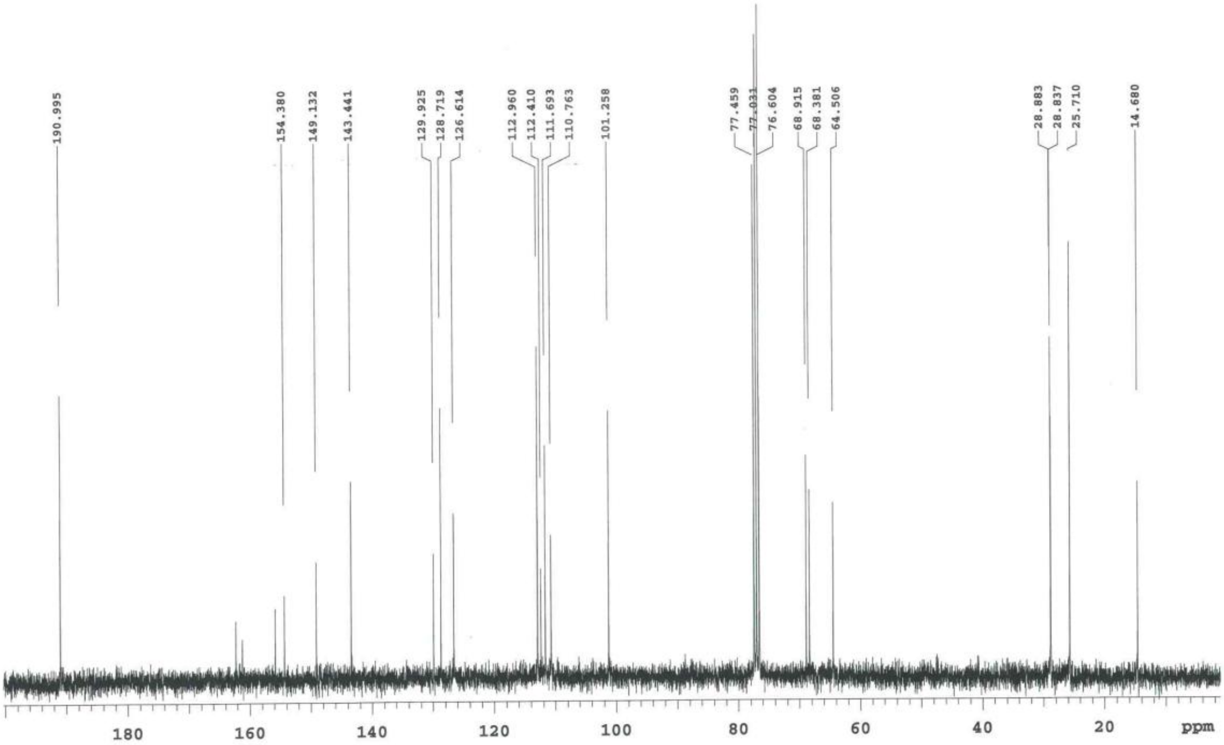

Figure S40. ^{13}C NMR of compound **24**.

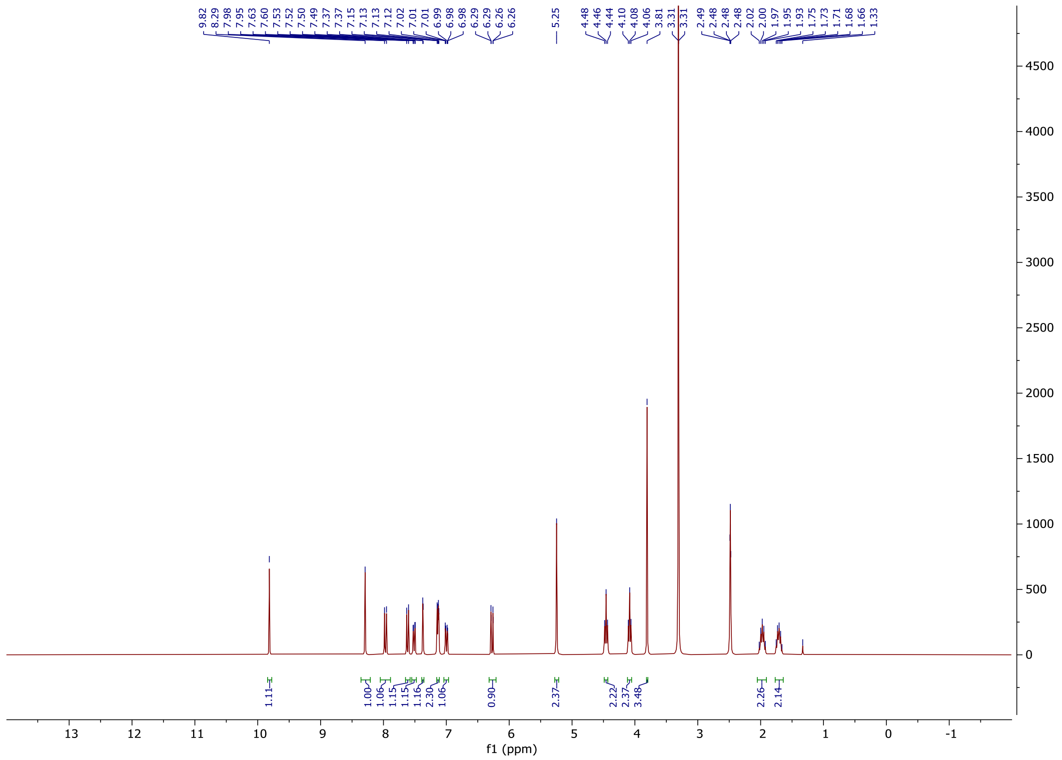

Figure S41. ^{1}H NMR of compound **25**.

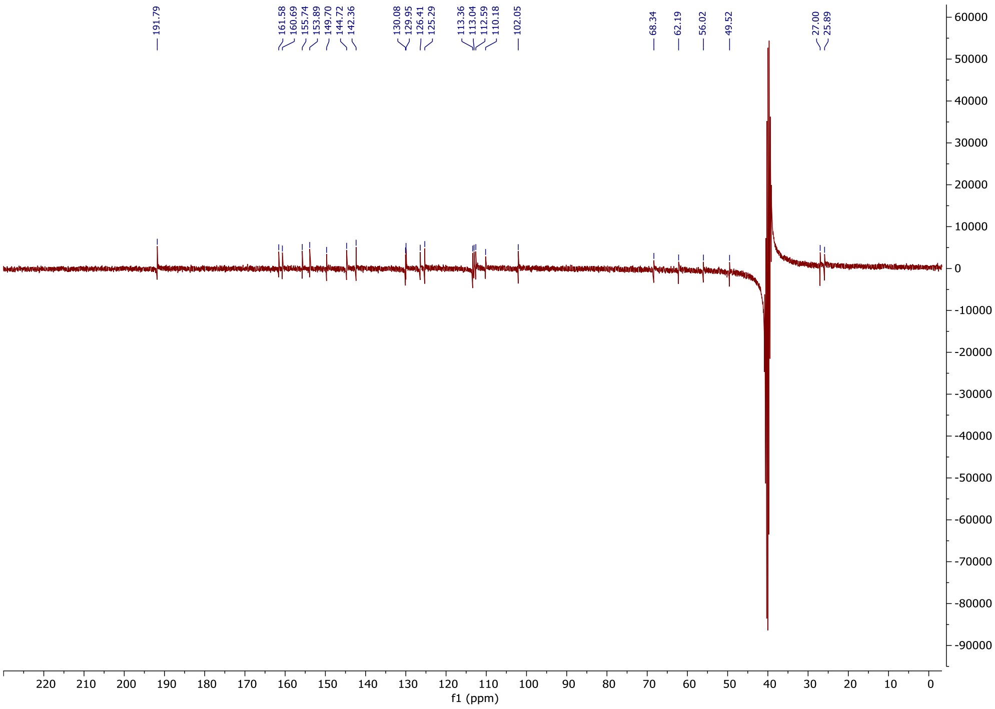

Figure S42. ^{13}C NMR of compound **25**.

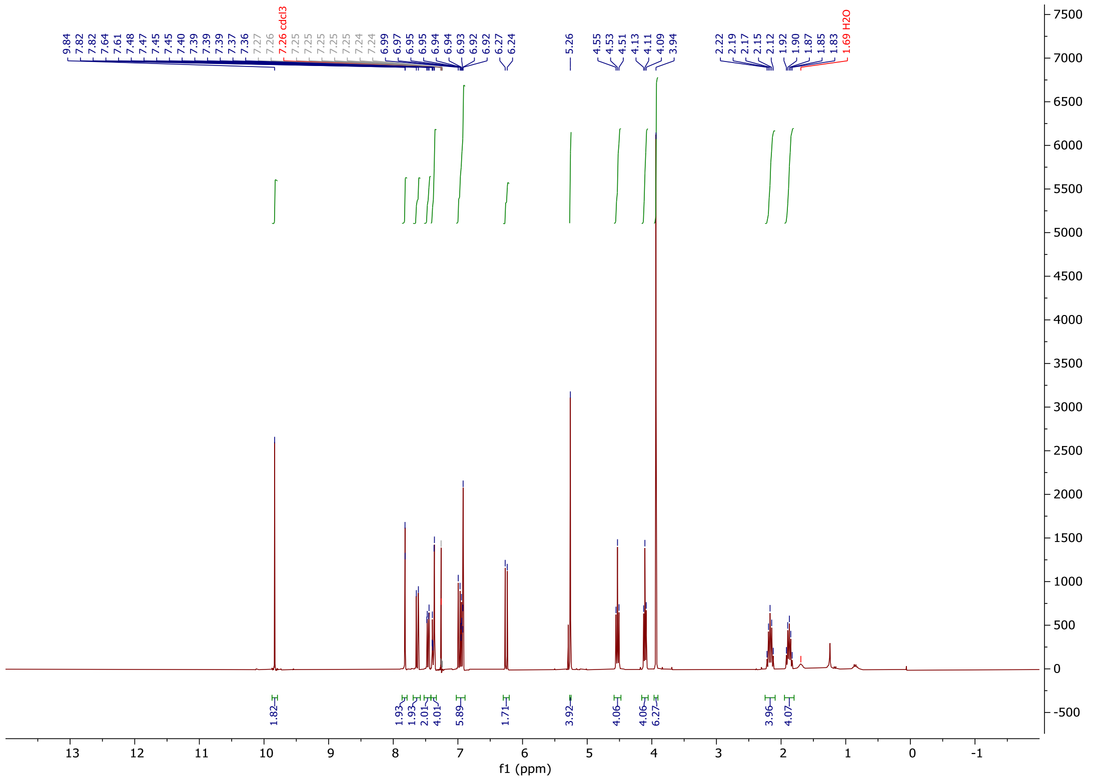

Figure S43. ^{1}H NMR of compound **26**.

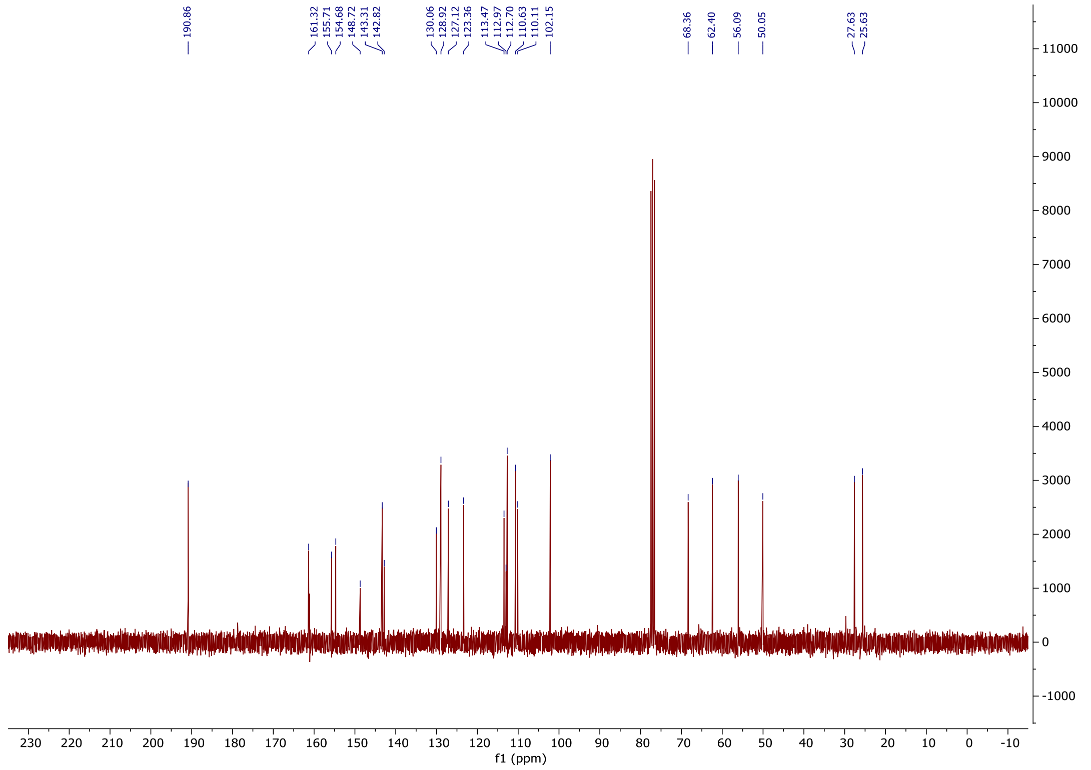

Figure S44. ^{13}C NMR of compound **26**.

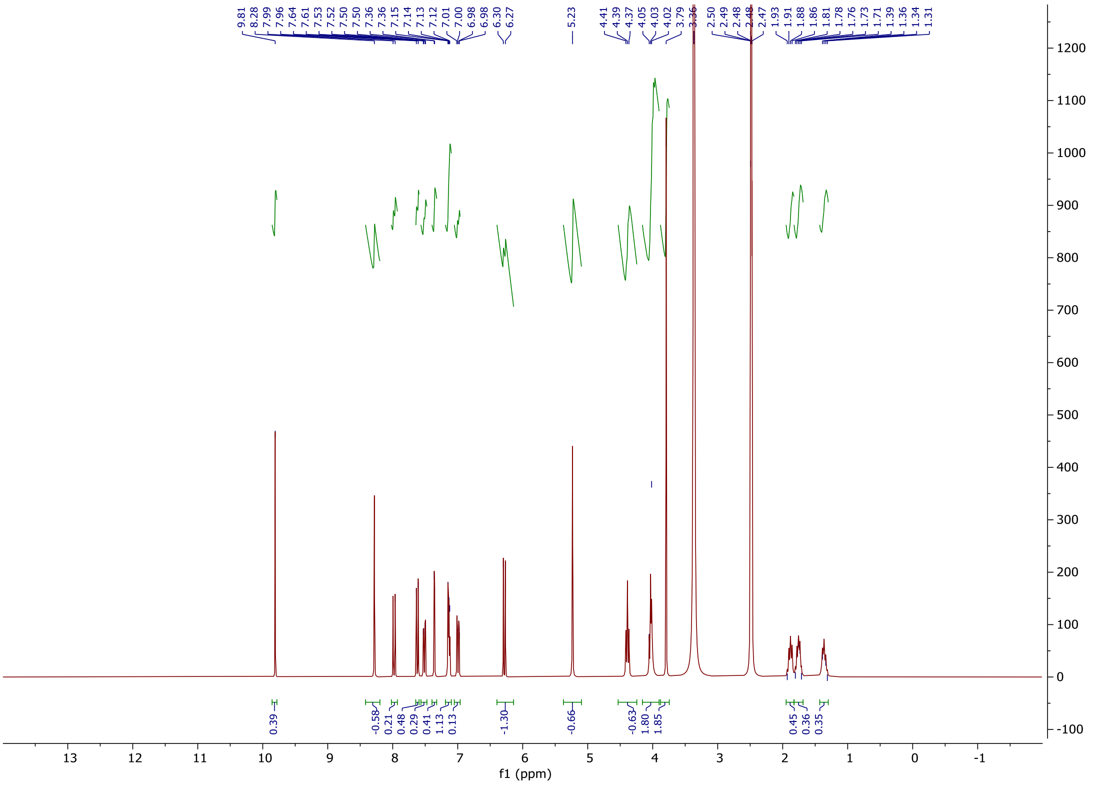

Figure S45. ^{1}H NMR of compound **27**.

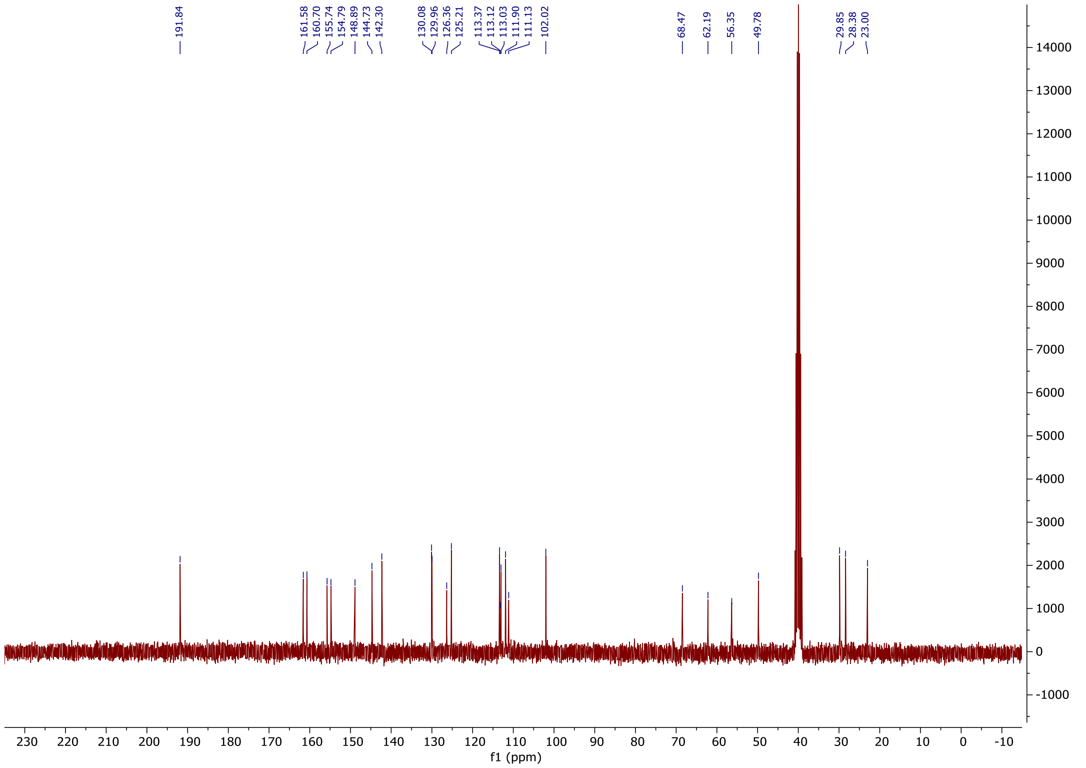

Figure S46. ^{13}C NMR of compound **27**.

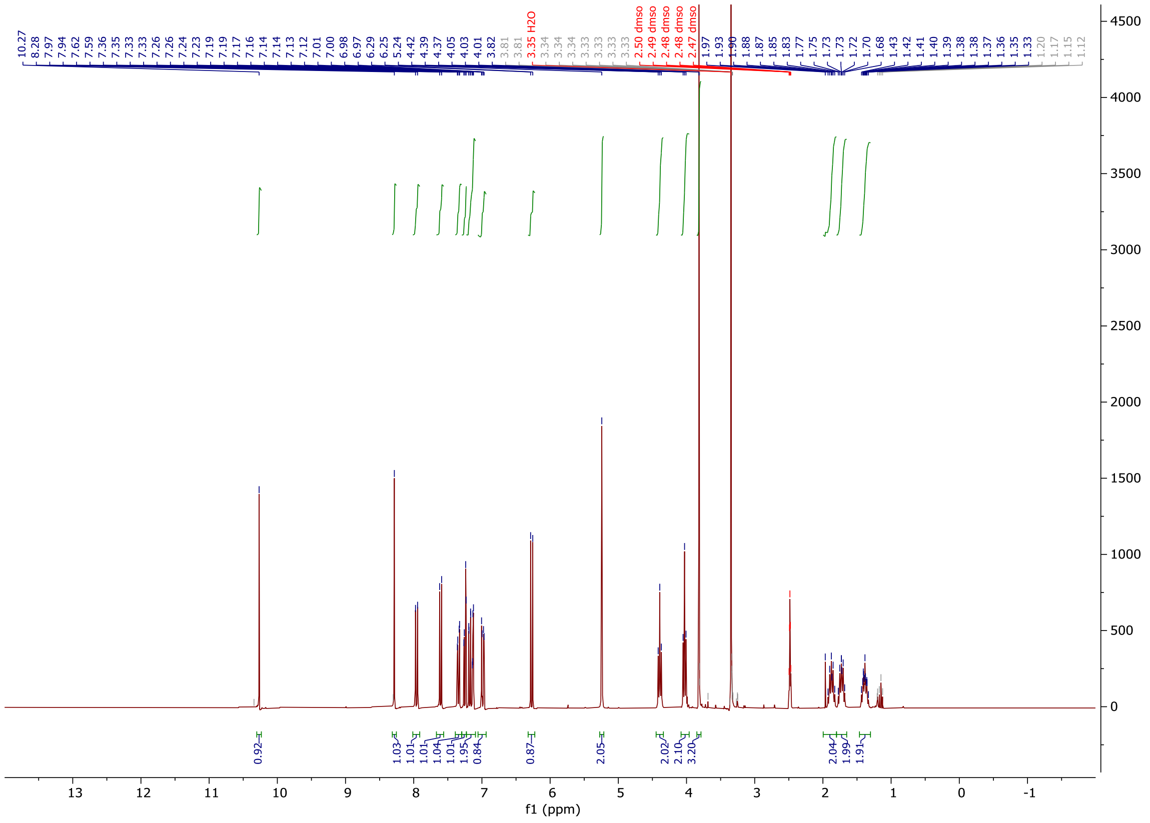

Figure S47. ^{1}H NMR of compound **28**.

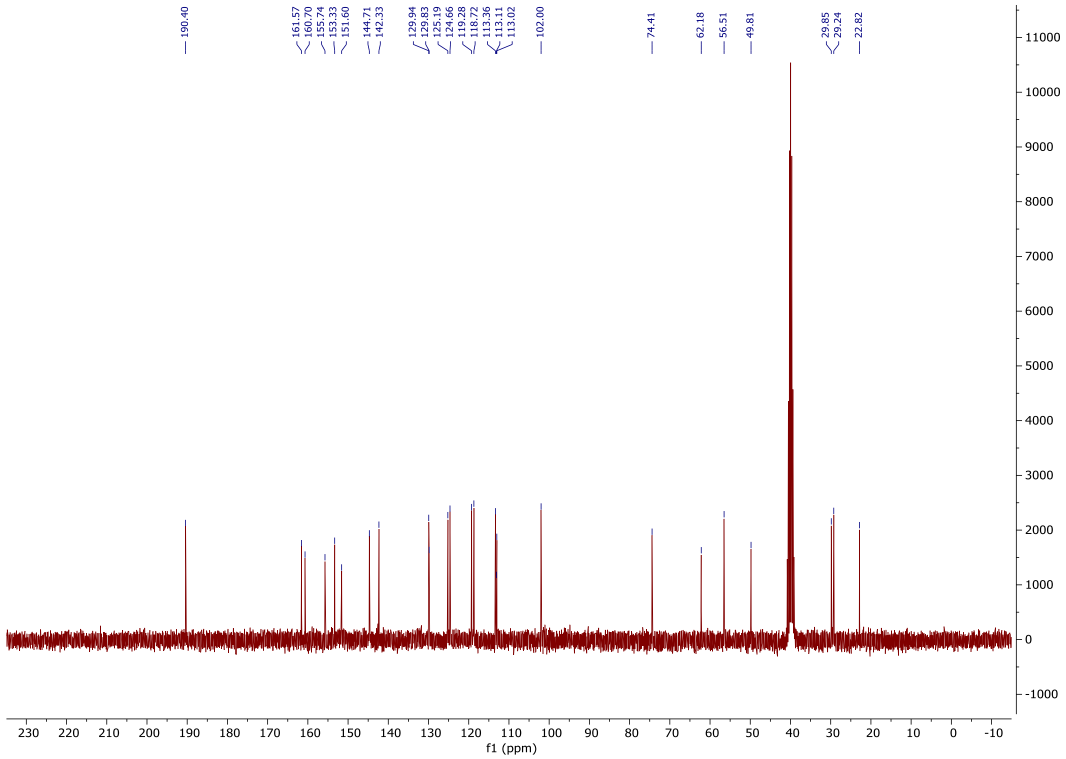

Figure S48. ^{13}C NMR of compound **28**.

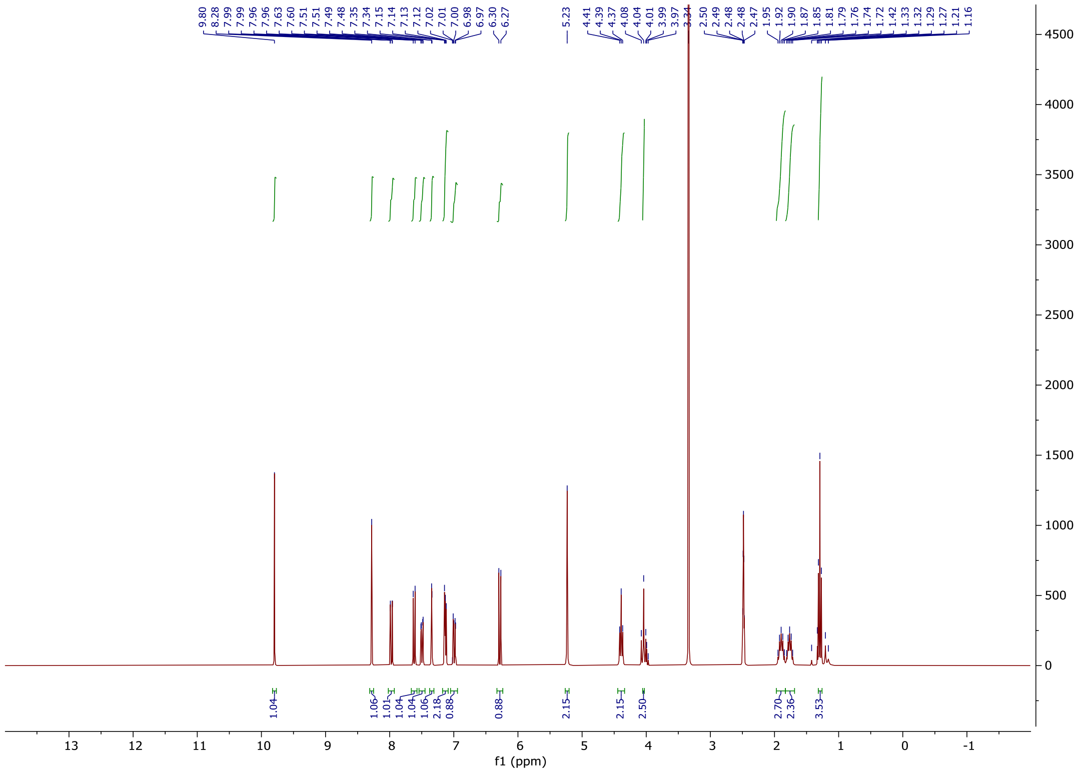

Figure S49. ^{1}H NMR of compound **29**.

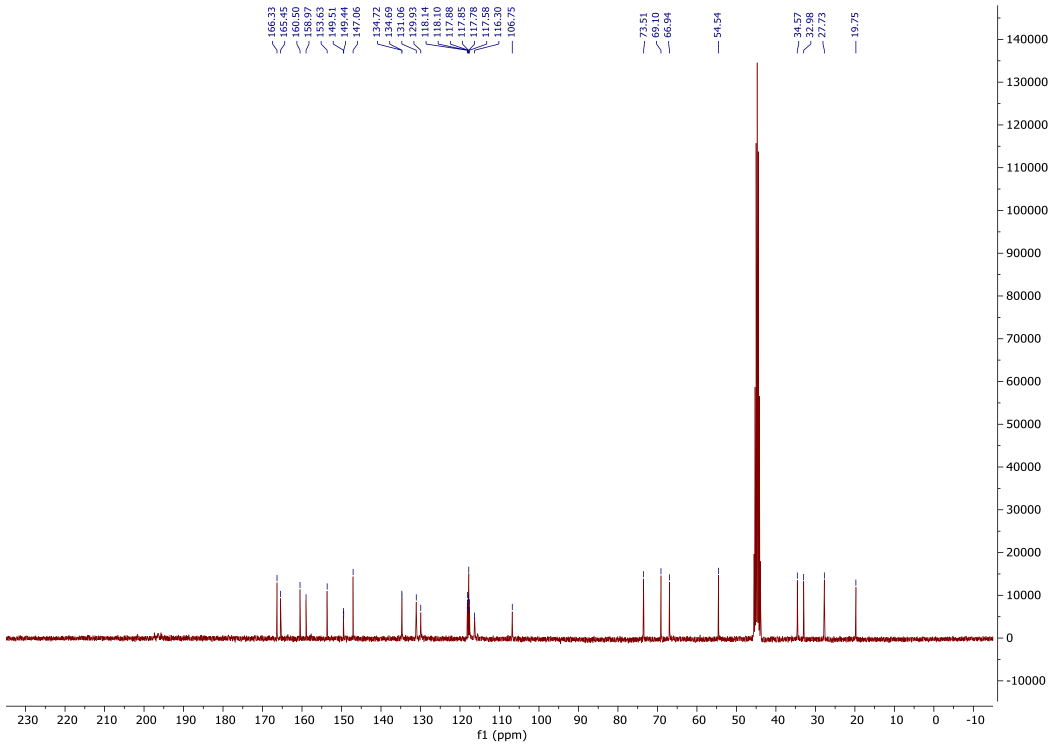

Figure S50. ^{13}C NMR of compound **29**.

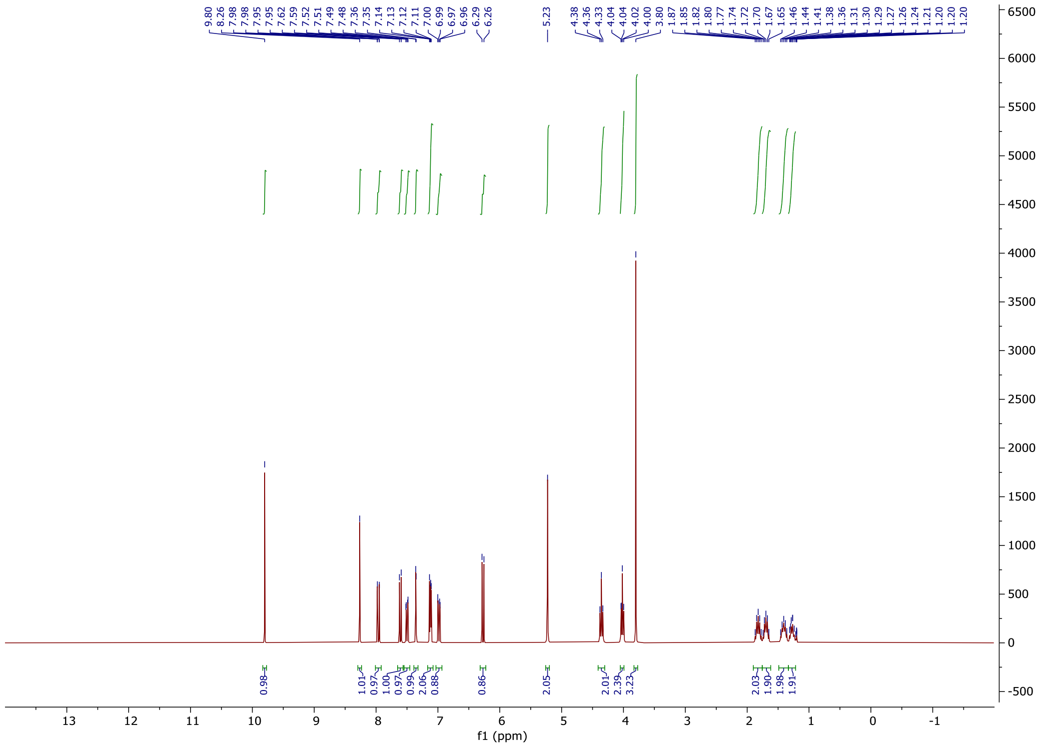

Figure S51. ^{1}H NMR of compound **30**.

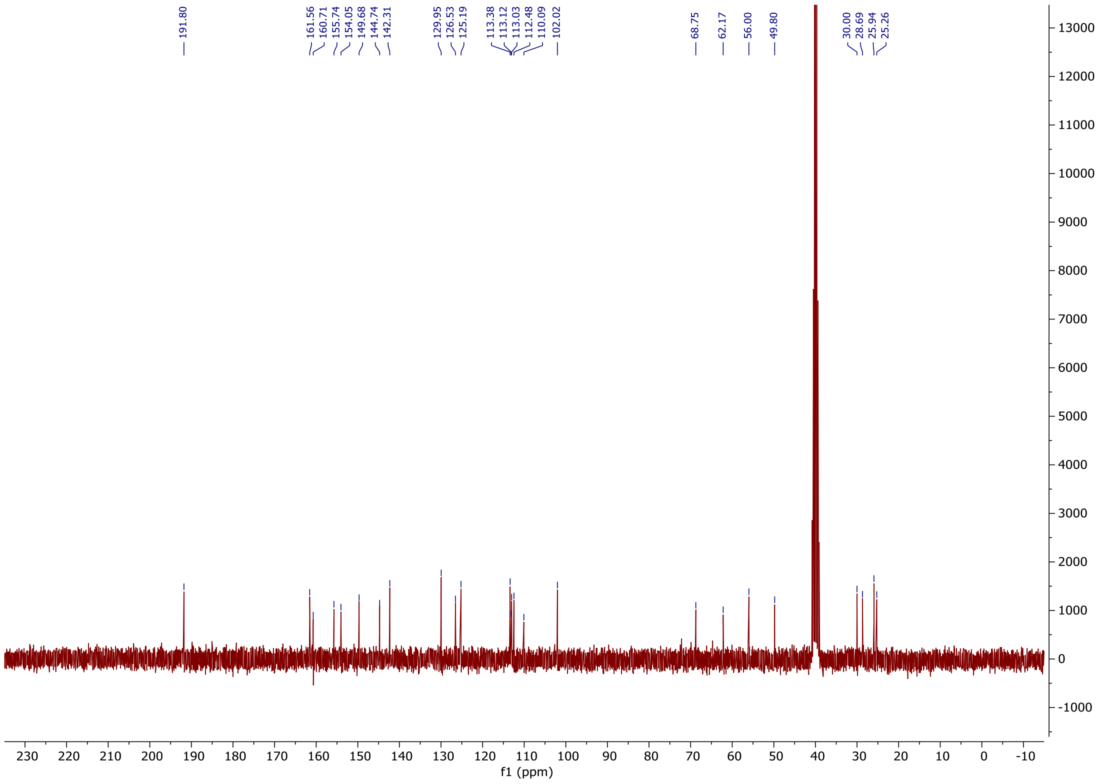

Figure S52. ^{13}C NMR of compound **30**.

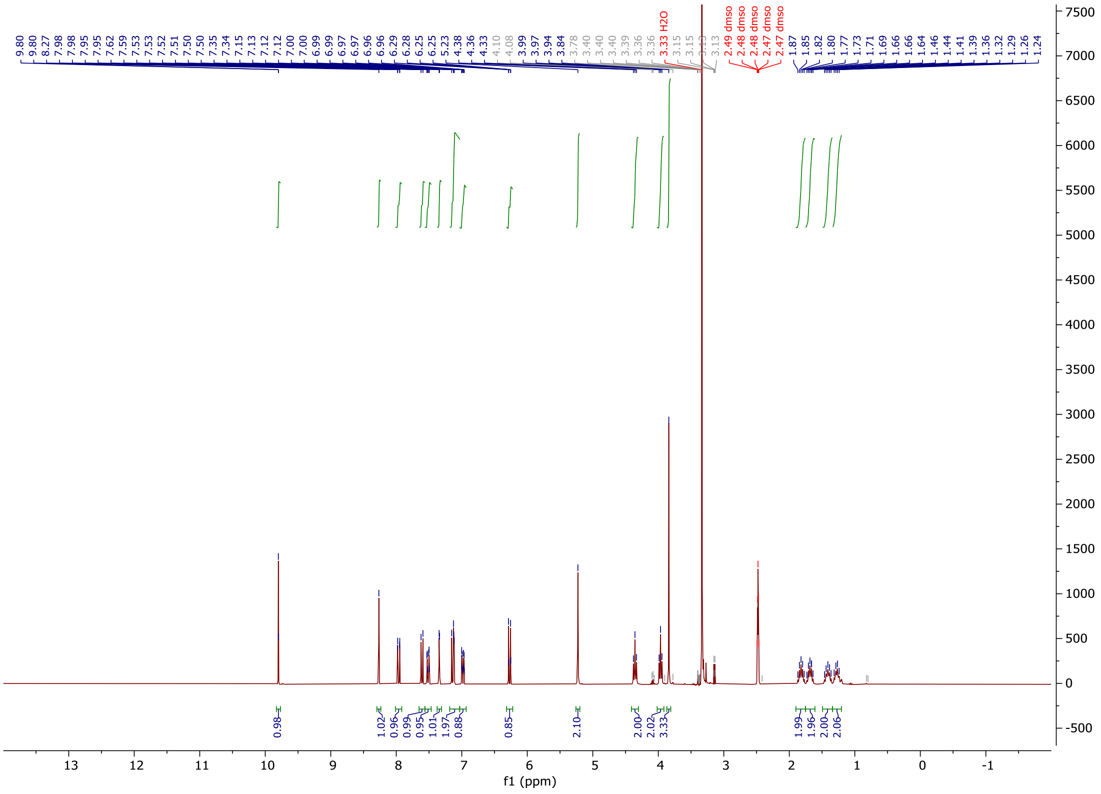

Figure S53. ^{1}H NMR of compound **31**.

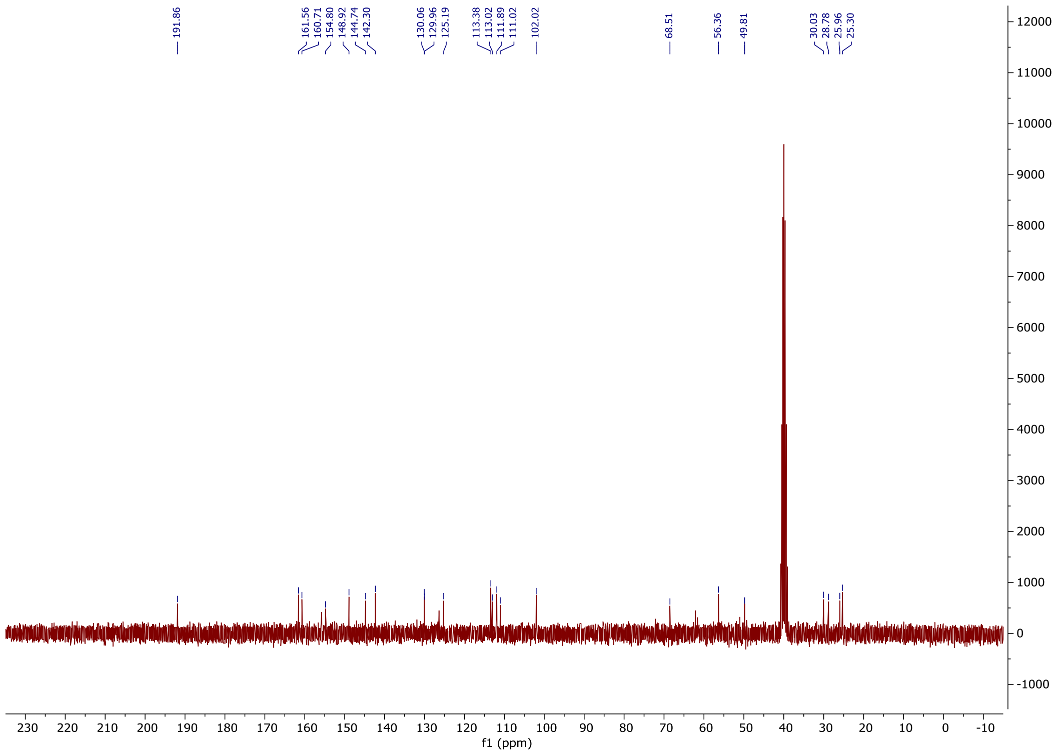

Figure S54. ^{13}C NMR of compound **31**.

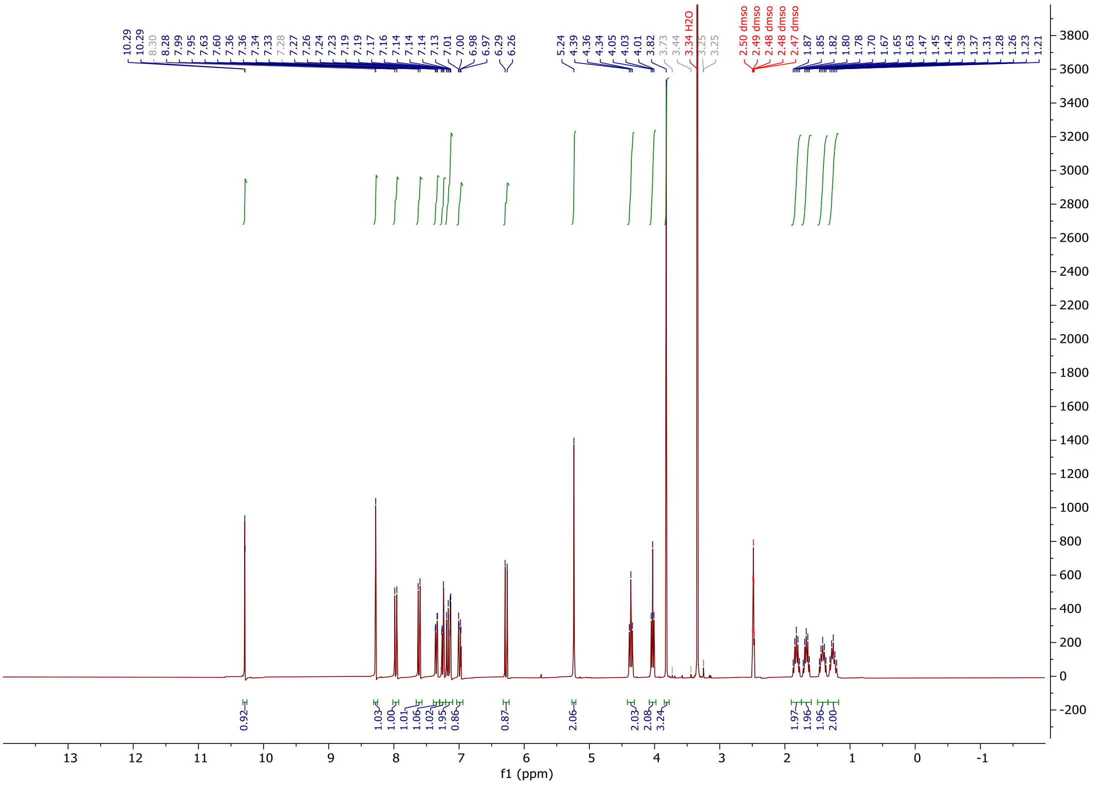

Figure S55. ^{1}H NMR of compound **32**.

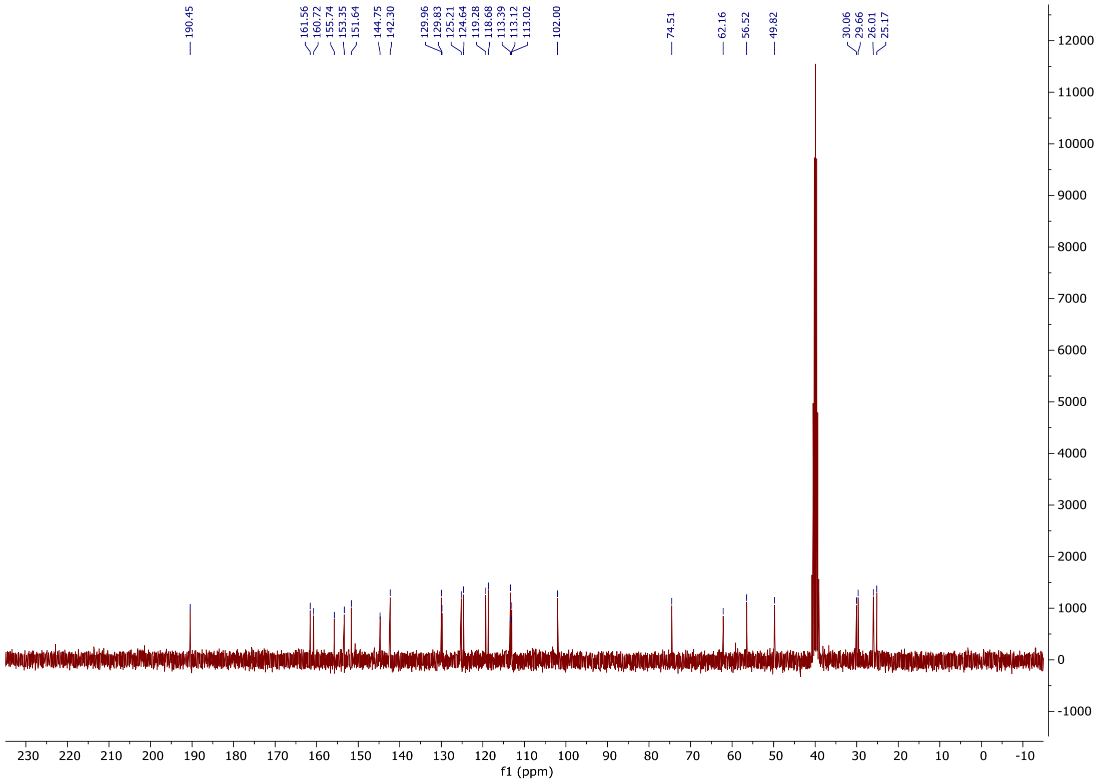

Figure S56. ^{13}C NMR of compound **32**.

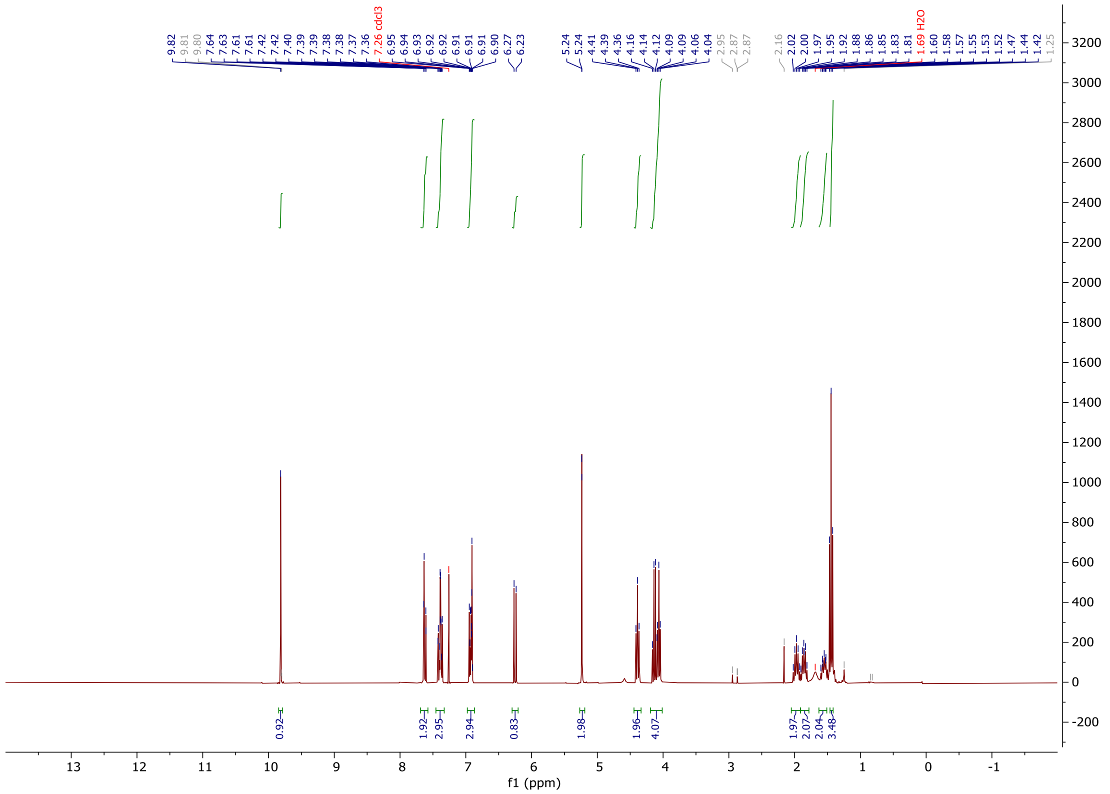

Figure S57. ^{1}H NMR of compound **33**.

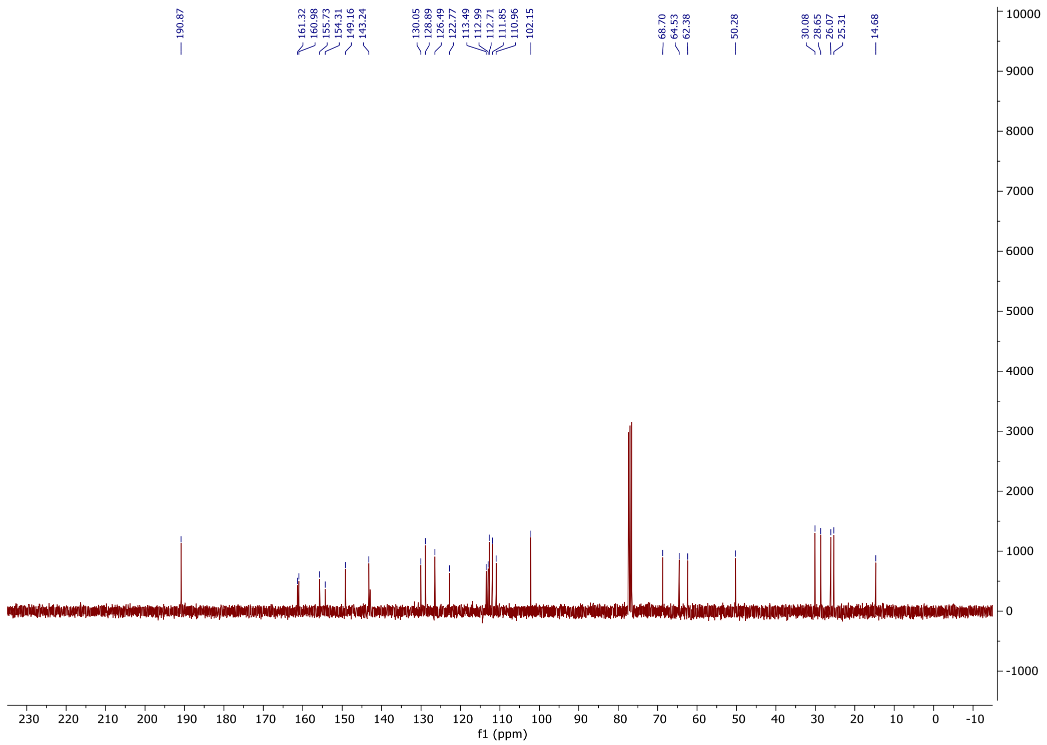

Figure S58. ^{13}C NMR of compound **33**.
